# Is moderate resistance training adequate for older adults with sarcopenia? A systematic review and network meta-analysis of RCTs

**DOI:** 10.1186/s11556-023-00333-4

**Published:** 2023-11-29

**Authors:** Yu Chang Chen, Wang-Chun Chen, Chia-Wei Liu, Wei-Yu Huang, ICheng Lu, Chi Wei Lin, Ru Yi Huang, Jung Sheng Chen, Chi Hsien Huang

**Affiliations:** 1 Department of Family Medicine and Community Medicine, E-Da Hospital, I-Shou University, No. 1, Yida Road, Jiaosu Village, Yanchao District, Kaohsiung City, 82445 Taiwan; 2 Department of Pharmacy, E-Da Hospital, I-Shou University, No. 1, Yida Rd., Jiaosu Village, Yanchao District, Kaohsiung City, 82445 Taiwan; 3 Department of Chemical Engineering and Institute of Biotechnology and Chemical Engineering, I-Shou University, No.8, Yida Rd., Jiaosu Village, Yanchao District, Kaohsiung City, 82445 Taiwan; 4 School of Medicine for International Students, College of Medicine, I-Shou University, No. 8, Yida Rd., Jiaosu Village, Yanchao District, Kaohsiung City, 82445 Taiwan; 5 Data Science Degree Program, National Taiwan University and Academia sinica, No.1, Section 4, Roosevelt Rd, Da’an District, Taipei City, 10617 Taiwan (R.O.C.); 6 Department of Medical Research, E-Da Hospital, I-Shou University, No. 1, Yida Rd., Jiaosu Village, Yanchao District, Kaohsiung City, 82445 Taiwan

**Keywords:** Sarcopenia, Network meta-analysis, Resistance training intensity

## Abstract

**Background:**

Resistance training (RT) and nutritional supplementation are recommended for the management of sarcopenia in older adults. However, optimal RT intensity for the treatment of sarcopenia has not been well investigated.

**Methods:**

This network meta-analysis aims to determine the comparative effectiveness of interventions for sarcopenia, taking RT intensity into consideration. RT intensity was classified into light-to-moderate intensity RT(LMRT), moderate intensity RT(MRT), and moderate-to-vigorous intensity RT(MVRT) based on percentage of one repetition maximum (%1RM) and/or rating of perceived exertion.

**Results:**

A total of 50 RCTs (*N* = 4,085) were included after screening 3,485 articles. The results confirmed that RT with or without nutrition was positively associated with improved measures of muscle strength and physical performance. Regarding RT intensity, LMRT only demonstrated positive effects on hand grip (aerobic training + LMRT + nutrition: mean difference [MD] = 2.88; 95% credential intervals [CrI] = 0.43,5.32). MRT provided benefits on improvement in the 30-s chair stand test (repetitions) (MRT: MD = 2.98, 95% CrI = 0.35,5.59), timed up and go test (MRT: MD = -1.74, 95% CrI: = -3.34,-0.56), hand grip (MRT: MD = 2.44; 95% CrI = 0.03,5.70), and leg press (MRT: MD = 8.36; 95% CrI = 1.87,13.4). MVRT also improved chair stand test repetitions (MVRT: MD = 5.64, 95% CrI = 0.14,11.4), gait speed (MVRT + nutrition: MD = 0.21, 95% CrI = 0.003,0.48), appendicular skeletal muscle index (MVRT + nutrition: MD = 0.25, 95% CrI = 0.01,0.5), and leg press (MVRT: MD = 14.7, 95% CrI: 5.96,22.4; MVRT + nutrition: MD = 17.8, 95% CrI: 7.55,28.6).

**Conclusion:**

MVRT had greater benefits on muscle mass, lower extremity strength, and physical performance compared to MRT. Increasing RT intensity may be recommended for sarcopenic older adults.

**Supplementary Information:**

The online version contains supplementary material available at 10.1186/s11556-023-00333-4.

## Introduction

Sarcopenia, an age-related condition characterized by progressive decrease in muscle mass, strength, and function, currently affects an estimated 10–40% of community-dwelling older adults [1, 2]. Additionally, sarcopenia is associated with increased risk of falls by 60%, increased fractures by 84%, and adverse health outcomes such as functional decline, decreased quality of life, mortality, and increased healthcare costs [1, 3, 4].

Modifiable risk factors including low physical activity and protein intake have been targeted for the prevention and treatment of sarcopenia [5]. In 2018, the International Conference on Sarcopenia and Frailty Research (ICSFR) guideline for the management of sarcopenia recommended progressive resistance training (RT) and a protein-rich diet or protein supplementation [6]. Recent systematic reviews and meta-analyses have demonstrated desirable effects of various forms of exercise with or without nutrition interventions on muscle strength and physical performance, as measured by gait speed (GS) or short physical performance battery (SPPB) [7–9]. However, the evidence for increasing muscle mass is less consistent. One meta-analysis focusing on sarcopenic older adults found no improvement after exercise, nutrition, and mixed exercise (aerobic training (AT) plus RT) and nutrition [8], while another meta-analysis published in the same year determined that mixed exercise with nutrition resulted in significantly increased muscle mass among people with sarcopenia [10]. Discrepancies in study results may be due to varied inclusion criteria, different definitions of sarcopenia used, and inconsistent exercise protocols in exercise type, frequency, intensity, and duration.

More importantly, exercise intensity, especially for RT, has not been fully taken into consideration in previous systematic reviews and meta-analyses. ACSM guidelines suggest moderate-to-vigorous RT intensity (60–80% one-repetition maximum, 60–80%1RM) of resistance exercise for older adults [11]. Recent systematic reviews and meta-analyses suggest that progressive RT may reduce mortality and produce greater gains in muscle strength in a linear fashion among older adults in general [12–14]. On the other hand, one meta-analysis focusing on older adults reported that high-load RT only produced marginal gains in muscle mass and insignificant improvements in muscle strength [15]. According to Csapo et al. because muscle hypertrophy plateaus above a certain point in high intensity training, high frequency low intensity training may be required to continue increasing muscle mass [15]. Additionally, high-intensity exercise might decrease adherence and lead to decline in total exercise [16]. Thus, clarification of the effects of RT intensity on muscle mass, strength, and physical performance is needed to make precise exercise prescriptions for older adults with sarcopenia.

The objective of this study is to compare the effectiveness of interventions for sarcopenia, with a particular focus on determining the optimal intensity of RT for older adults with sarcopenia. We conducted a network meta-analysis of randomized controlled trials (RCTs) in older adults with sarcopenia and pooled data of intervention effects on muscle mass (appendicular skeletal muscle, leg muscle mass, and skeletal muscle mass), muscle strength (handgrip strength (HG), chest press, and leg press), and physical function (5 times sit to stand (5TSTS), number of repetitions done in the 30-s chair stand test, timed up and go test (TUG), SPPB, GS, and 6-min walk test).

## Methods

This network meta-analysis was performed according to the standards described in the Preferred Reporting Items for Systematic Reviews and Meta-analysis (PRISMA) statement [17]. The study was registered in PROSPERO under the ID CRD42021287114.

### Search strategy and selection process

Using Pubmed, Embase, Central Register of Controlled Trials (CENTRAL), and ClinicalTrials (Clinicaltrials.gov), we identified RCTs on sarcopenia from database inception until October 20, 2022. The keywords used for the search were “sarcopenia” or “sarcopeni*” and “randomized controlled trial.” To identify pertinent studies, we utilized the search terms: “train*”, “physical activity”, “exercise”, “diet”, “nutr*”, and “drug therapy”. Additionally, we incorporated the associated MeSH terms: “sarcopenia”, “exercise”, “diet, food, and nutrition”, “nutrition therapy”, and “drug therapy”. "We integrated the search terms using the Boolean operators “AND” and “OR”. The complete search terms and search string can be found in Supplementary S1. To ensure comprehensive inclusion of potentially relevant articles, we refrained from applying filters related to publication type, age, or language. Additional studies were identified by reviewing the reference lists of papers found through the database search. Study protocol paper and conference abstracts were not included. The inclusion criteria for studies were: (1) community-dwelling adults aged over 18 years, (2) participants diagnosed with either sarcopenia (characterized by low muscle mass and low muscle strength, and/or reduced physical performance) or dynapenia (manifested as low muscle strength and/or reduced physical performance but with normal muscle mass) [18, 19], and (3) RCT. Since severe illness such as cancer, liver cirrhosis, or end stage renal failure could induce cachexia and decrease physical function, studies involving patients with these comorbidities were excluded.

**Fig. 1:**
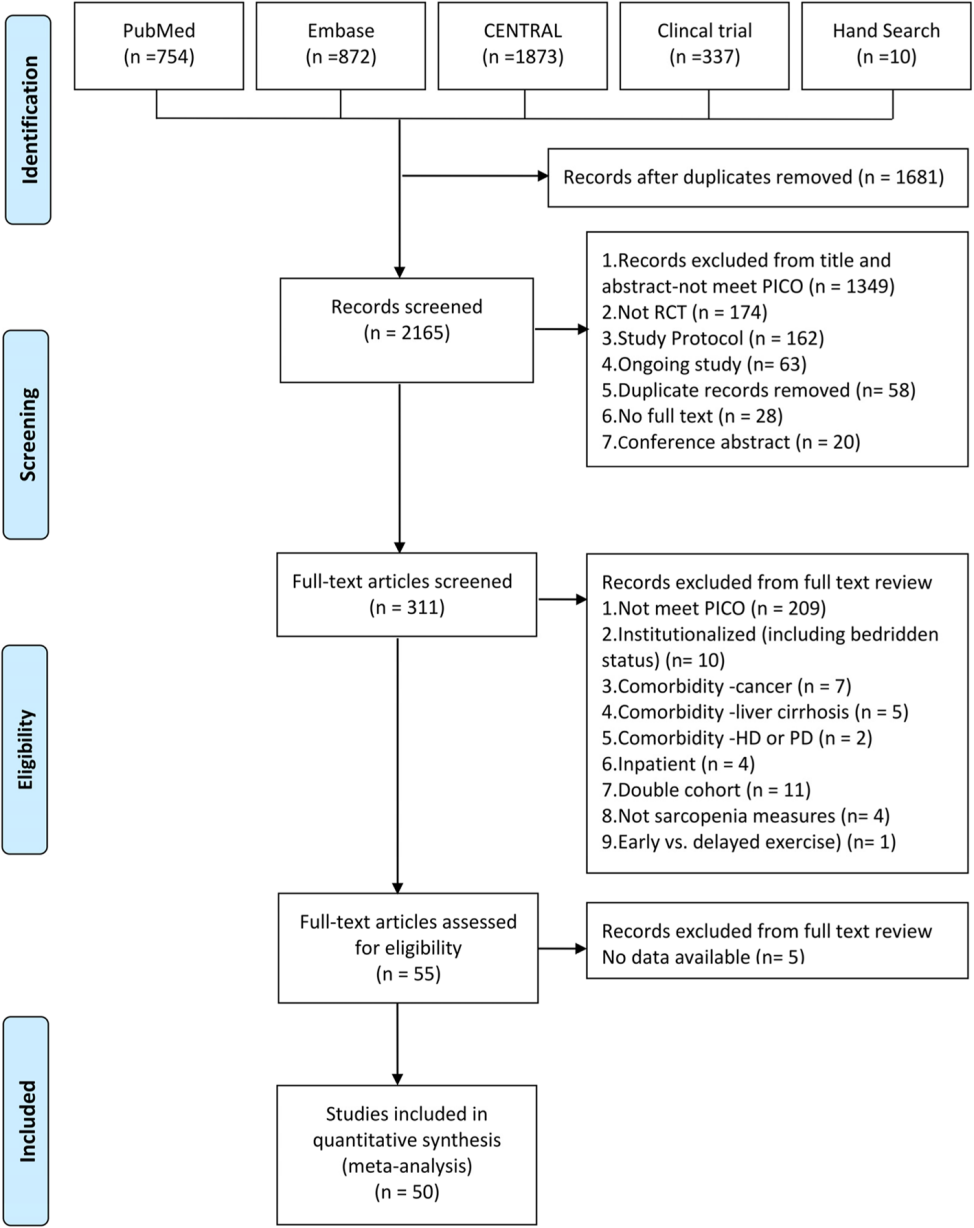
PRISMA flow diagram

During the initial selection process, two independent authors reviewed the title, abstract, and full text of each reference to determine its suitability for inclusion. In cases of uncertainty regarding the study’s relevance, a third author was consulted to achieve consensus. When multiple studies on the same population were conducted by the same research group and reported identical outcomes of interest, we only chose the results from the study with the longest follow-up duration. The process of selection is detailed in Fig. 1. Finally, the included studies underwent a comprehensive assessment of bias risk using the Cochrane Risk of Bias Tool 2.0 (RoB 2.0) [20], accessible at https://methods.cochrane.org/risk-bias-2. This tool evaluates each study’s susceptibility to potential bias across multiple domains, including randomization procedures, adherence to intended interventions, handling of missing outcome data, measurement of outcome variables, and selection bias. We categorized the overall risk of bias in each domain as “Low risk of bias,” “Some concerns,” or “High risk of bias.”

### Data extraction

RCTs with at least one intervention (e.g., nutrition, exercise, whole body electrical muscle stimulation [WB-EMS], whole body vibration [WBV], electrical puncture, Taichi, global sensorimotor training, focused vibrational therapy, and drug treatment [bimagrumab, MK-0773, perindopril, oxytocin]) were included. One researcher entered the following data for each paper into a standardized table: authors, publication year, location of study, number of participants, baseline characteristics of participants, inclusion criteria, exclusion criteria, intervention(s), comparison group, duration of intervention, intensity of resistance exercise, and outcomes of interest. Outcome measures included 5TSTS, number of repetitions done in the 30-s chair stand test, TUG, SPPB, GS, 6-min walk test, appendicular skeletal muscle index (ASMI), leg muscle mass, skeletal muscle mass, HG, chest press, leg press. Since the quality of life (QOL), as measured by either the Short Form 36 or Short Form 12, is divided into physical and mental components, the combined QOL is represented using overall, physical, and psychological scores.

### Grading of exercise intensity

Exercise was initially classified as either AT or RT. According to ACSM guidelines, the intensity of RT was categorized into five levels: very light, light, moderate, vigorous, and near-maximal to maximal intensity, based on repetition maximum (RM) and/or rating of perceived exertion (RPE) [11]. The term “1RM” refers to the maximum weight an individual can lift for a single repetition. Relative intensity, indicated as percentage of 1RM (%1RM), was calculated by converting from the repetition numbers implemented in the RT program [21]. The Borg RPE is a subjective scale and reliable measure of RT intensity [22]. However, the sarcopenia management guidelines advocate for RT of at least moderate intensity [23]. Accordingly, we stratified RT intensity into 3 distinct levels: light-to-moderate (LMRT), moderate (MRT), and moderate-to-vigorous intensity RT (MVRT). Specifically, LMRT corresponds to scores of 6–11 on the Borg RPE scale (whose full range is 6–20), 0–4 on the Modified Borg’s scale (with a complete range of 0–10), or less than 49% of 1RM [11]. MRT is represented by ratings of 12–13 on the Borg RPE scale, 5–6 on the Modified Borg’s scale, or 50% ~ 69% of 1RM [11]. MVRT is characterized by scores of 14–17 on the Borg RPE scale, 7–8 on the Modified Borg’s scale, or 70% ~ 84% of 1RM [11]. To ensure accuracy, both a sports medicine physician and a geriatrician meticulously reviewed all included studies. They then determined the RT intensity through mutual consensus.

AT primarily focuses on augmenting cardiovascular endurance and efficiency. Nonetheless, it also leads to discernible enhancements in muscular strength and endurance [24]. Given that 50% of 1RM is roughly equivalent to an average of 26 repetitions [25], AT, which typically involves over 100 repetitive movements, can be categorized as LMRT. Thus, the effects of AT on muscular strength and endurance might be more subtle compared to those elicited by RT. Consequently, we opted not to further classify AT.

### Statistical analysis

Network meta-analysis was performed using changes in mean and standard deviation (SD) from baseline. 95% two-tailed credible intervals (CrI) were calculated, with *p* < 0.05 indicating statistical significance. When studies only reported 25% and 75% percentile of outcome values, we estimated SD based on interquartile range (IQR = 1.349*SD) [26]. If changes in SD were not available, it was estimated using the following equation: [SDpre^2^ + SDpost^2^-2 × CC × SDpre × SDpost]^0.5^ [9, 27]. SDpre represented the SD at baseline and SDpost was the SD after the intervention. CC was the correlation coefficient between baseline and post-intervention values for the same individual. If the correlation was not reported, CC was designated as 0.5. Network plots visually represented the number of study participants according to the size of nodes and the number of trials conducted according to the thickness of connecting lines. Forest plots depicted the intervention effects compared to the control group. Effectiveness of the interventions were ordered by rank probability and determined using the surface under the cumulative ranking curve (SUCRA), where larger surface areas equaled greater treatment effects [28].

We used the web‐based software MetaInsight V4.0.0 powered by Rshiny for network meta-analysis combining direct and indirect comparisons and figure plotting [29, 30]. All Bayesian statistical calculations were performed using R package gemtc [28]. Random effects model by heterogeneity consideration was employed because results under random effects model in all analyses demonstrated better fitting with lower deviance information criterion values when compared to fixed effects model [31]. Bayesian rank probabilities were visualized with (cumulative) Rank-O-Grams. Publication bias was examined by Egger’s regression [32]. The consistency of network meta-analysis was assessed using the node-splitting models to compare the results between direct and indirect comparisons [33]. Several sensitivity and subgroup analyses were conducted to ensure consistency and stability of results. Analyses were repeatedly performed by (1) sequentially excluding each trial and (2) omitting studies with a high risk of bias.

## Results

### Study selection

The PRISMA flow diagram (Fig. 1) shows the study selection process and provides reasons for study exclusion. A total of 3,846 publications were identified from 4 databases and hand search, of which 1,681 duplicate records were removed. After screening titles and abstracts based on the pre-specified criteria, the full text of 311 records were assessed for eligibility. After full-text review, 55 records were retained. Five more articles were removed due to lack of data. Therefore, a final total of 50 RCTs were included in quantitative network meta-analysis. The results of risk of bias assessments using ROB2 are shown in Table S1.

### Study characteristics

Table 1 shows characteristics and details of the included studies. The 50 studies included (*N* = 4,085, mean age range:55.0 ± 9.6 to 89.5 ± 4.4 years) were published between 2012–2022 and were conducted around the globe, mostly in Asia (7 studies in Taiwan, 4 in South Korea, 3 in China, and 2 in Japan). Three studies were conducted cross-nationally in Europe and North America. There were 14 studies involving RT, 5 studies involving AT + RT, 7 studies involving nutritional intervention, 1 study involving AT + nutritional intervention, 9 studies involving RT + nutritional intervention, and 4 studies involving AT + RT + nutritional intervention, and 6 studies involving WB-EMS and WBV. Adherence rates to the exercise intervention ranged from 74 to 100%, with no apparent correlation to exercise intensity. Details of the included trials are shown in Table S2.

**Table 1:** Descriptive characteristics of included studies

**Study and Year**	Age (years)	M/F	Intervention	Intervention type	Intensity of resistance exercise	Comparison Group	Duration	Sarcopenia Outcomes
**Alemán-Mateo et al. (2012) **[34]	76 ± 5.4	17/23	Dietary protein supplement	Nutrition	–	Habitual diet and exercise	3 months	Muscle mass, handgrip strength
**Bellomo et al. (2013) **[35]	70.9 ± 5.2	40/0	Global sensorimotor training, resistance training, focused vibrational therapy	RT	Vigorous	Usual daily habits concerning diet, social relations and physical activity	12 weeks	Maximal force contraction of the lower limbs; static and dynamic balance confidence
**Papanicolaou et al. (2013) **[36]	75.9 ± 6.9	0/170	Selective androgen receptor modulator (MK-0773) with protein and vitamin D supplement	Drug	–	Placebo tablet with protein and vitamin D supplement	6 months	Gait speed, SPPB, muscle mass
**Liu et al. (2014) **[37]	77.5 ± 4.2	8/25	Mixed aerobic, strength, balance, and flexibility training	AT, RT	Moderate	Health education	12–18 months	Gait speed, SPPB, sarcopenia status
**Bauer et al. (2015) **[38]	Intervention: 77.3 ± 6.7 Control: 78.1 ± 7.0	131/249	Whey protein and vitamin D supplement	Nutrition	–	Iso-caloric control product without protein	13 weeks	Muscle mass, handgrip strength, gait speed, SPPB, chair-stand test
**Zdzieblik et al. (2015) **[39]	72.2 ± 4.68	53/0	Collagen protein supplement and resistance exercise	Nutrition, RT	Vigorous	Placebo supplement and resistance exercise	3 months	Muscle mass, muscle strength
**Cramer et al. (2016) **[40]	77 (71, 81)^a^	125/205	Oral nutritional supplement with 20 g protein	Nutrition	–	Oral nutritional supplement with 14 g protein	6 months	Leg strength, body weight, muscle mass, handgrip strength, gait speed
**Kemmler et al. (2016) **[41]	Electromyostimulation: 77.3 ± 4.9 Electromyostimulationand protein: 76.4 ± 2.9 Control: 77.4 ± 4.9	0/75	Whole-body electromyostimulation and protein supplement	Electromyo-stimulation, nutrition	–	No treatment	26 weeks	ASMI, gait speed, grip strength
**Kim et al. (2016) **[42]	Exercise and Nutrition: 80.9 ± 4.2 Exercise: 81.4 ± 4.3 Nutrition: 81.2 ± 4.9 Control: 81.1 ± 5.1	0/139	Amino acid supplement, tea, and mixed aerobic and resistance exercises	Nutrition, AT, RT	Mild	Health education	3 months	Muscle mass, knee extension strength, handgrip strength, walking speed
**Maltais et al. (2016) **[43]	Control: 64 ± 4.5 EAA supplements: 64 ± 7.8 EAA milk: 68 ± 5.6	26/0	Protein supplement and resistance exercise	Nutrition, RT	Vigorous	Placebo oral supplement and resistance exercise	4 months	Muscle mass, muscle strength, TUG, chair stand test
**Maltais et al. (2016) **[44]	65 ± 5	26/0	Protein supplement and resistance exercise	Nutrition, RT	Vigorous	Placebo oral supplement and resistance exercise	4 months	Body composition
**Maruya et al. (2016) **[45]	Intervention group: 69.2 ± 5.6 Control group: 68.5 ± 6.2	23/29	A 6-month home exercise programs, combining walking with lower limb resistance exercises	AT, RT	Mild	Usual care	6 months	Body mass index, skeletal mass index, body fat percentage, handgrip strength, single-leg standing, walking speed (comfortable and maximal), and knee extension strength, quality of life (EQ-5D)
**Rondanelli et al. (2016) **[46]	Dietary supplement group: 80.77 ± 6.29 Placebo group: 80.21 ± 8.54	53/77	Nutritional supplementation with whey protein (22 g), essential amino acids (10.9 g, including 4 g leucine), and vitamin D [2.5 m g (100 IU)] concurrent with physical activity (RT + balance and gait training)	Nutrition, RT	Moderate	Physical activity (RT + balance and gait training)	12 weeks	Body composition, muscle strength, blood biochemical indexes of nutritional and health status, and global nutritional status, physical function, quality of life (SF-36)
**Vasconcelos et al. (2016) **[47]	Exercise: 72 ± 4.6 No exercise: 72 ± 3.6	0/28	Resistance exercise	RT	Moderate to vigorous	No exercise intervention	10 weeks	Knee extensor strength, SPPB, gait speed, quality of life (SF-36)
**Chen et al. (2017) **[48]	Aerobic: 68.9 ± 4.4 Resistance: 69.3 ± 3.0 Combination: 68.5 ± 2.7 Control: 68.6 ± 3.1	10/50	Aerobic exercise, resistance exercise, or combined aerobic and resistance exercise	AT, RT	Moderate	No exercise intervention	2 months	Body composition, handgrip strength, knee extensor strength
**Huang et al. (2017) **[49]	Exercise: 68.89 ± 4.91 Control: 69.53 ± 5.09	0/35	Resistance exercise	RT	Moderate	Health education	3 months	Body composition
**Kemmler et al. (2017) **[50]	Electromyostimulation and protein: 77.1 ± 4.3 Protein: 78.1 ± 5.1 Control: 76.9 ± 5.1	100/0	Whole-body electromyostimulation and protein	Electromyo-stimulation, nutrition	–	No treatment	4 months	Body composition, ASM/BMI, handgrip strength
**Liao et al. (2017) **[51]	CG: 68.42 ± 5.86 EG: 66.39 ± 4.49	0/46	Elastic band resistance training	RT	Moderate	–	3 months	Fat-free mass, leg lean mass, absolute total fat mass, and body fat %, handgrip strength, muscle quality, single-leg stance, gait speed, TUG, and timed chair rise
**Park et al. (2017) **[52]	74.1 ± 6.1	0/50	Mixed aerobic and resistance exercise	AT, RT	Moderate to vigorous	Health education	6 months	ASM/weight, handgrip strength, 30-s chair stand test, maximum walking speed
**Sammarco et al. (2017) **[53]	55.0 ± 9.6	0/18	Low-calorie high-protein diet (1.2–1.4 g/kg body weight reference/ day obtained with the addition of 15 g daily of protein supplement)	Nutrition	–	Low-calorie diet plus placebo	4 months	Anthropometric measurements, body composition, resting energy expenditure, handgrip test, SPPB, quality of life (SF-36)
**Wei et al. (2017) **[54]	Low-frequency group: 78 ± 4 Medium- frequency group:75 ± 6 High-frequency group:74 ± 5 Control group:76 ± 6	24/56	Whole-body vibration (WBV) training	Vibration training	–	No training	Mid-intervention (week 6), post-intervention (week 12), mid-follow up (week18) and follow-up (week 24)	Muscle size, isometric and isokinetic knee extension strength measurements
**Wei et al. (2017) **[55]	Low-frequency group: 78 ± 4, medium- frequency group:75 ± 6, high-frequency group:74 ± 5, control group:76 ± 6	24/56	Whole-body vibration (WBV) training	Vibration training	–	No training	Mid-intervention (week6), post-intervention (week 12), mid-follow up (week18) and follow up (week24)	Five-repetition sit-to-stand, 10-m walking test with self-preferred speed and TUG
**Berens et al. (2018) **[56]	77.5 ± 5.4	80/69	Mixed aerobic and resistance activity program with protein and vitamin D nutritional supplement	Nutrition, AT, RT	Moderate	Physical activity program only	6 months	400 m walk capacity, quality of life (SF-36)
**Chen et al. (2018) **[57]	Exercise: 66.7 ± 5.3 No exercise: 68.3 ± 2.8	0/33	Kettleball resistance exercises	RT	Moderate to vigorous	No exercise intervention	2 months	Body composition, ASM, handgrip strength
**Kemmler et al. (2017) **[58]	Electromyostimulation and protein: 77.1 ± 4.3 Control: 76.9 ± 5.1	67/0	Whole-body electromyostimulation and protein	Electromyo-stimulation, nutrition	–	No treatment	4 months	Body composition, leg extension strength, gait speed
**Liao et al. (2018) **[59]	67.3 ± 5.1	0/56	Resistance exercise	RT	Moderate	–	3 months	Body composition, ASMI, handgrip strength, gait speed, TUG, 30-s chair stand test, SF-36 (physical functioning and physical component summary)
**Piastra et al. (2018) **[60]	Reinforcement training: 69.9 ± 2.7 Postural training: 70.0 ± 2.8	0/66	Muscle reinforcement training	RT	Mild to moderate	Postural training	9 months	Body composition, muscle mass, SMI, and handgrip strength
**Tsekoura et al. (2018) **[61]	72.87 ± 7.02	7/47	Muscle strengthening and balance exercises	RT	Moderate	Health education	3 months	Body composition, ASMI, handgrip strength, leg extension strength, quality of life (sarcopenia and Quality Of Life [SarQoL])
**Zhou et al. (2018) **[62]	Amino acid supplement: 68.80 ± 5.08 Electrical acupuncture and amino acid supplement: 70.35 ± 5.36	48/0	Essential amino acid supplementation with electrical acupuncture	Nutrition, acupuncture	–	Amino acid supplementation alone	3 months	Body composition, ASMI
**Amasene et al. (2019) **[63]	Exercise: 81.7 ± 6.45 Exercise and protein: 82.9 ± 5.59	14/28	Resistance exercise with protein supplement	Nutrition, RT	Moderate to vigorous	Resistance exercise with placebo supplement	3 months	Body composition, SPPB, handgrip strength
**Bo et al. (2019) **[64]	Protein supplement: 73.23 ± 6.52 Control: 74.83 ± 5.94	27/33	Protein, vitamin D, and vitamin E supplement	Nutrition	–	Iso-caloric control product without protein or vitamin D and E supplement	6 months	Muscle mass, handgrip strength, gait speed, chair stand test, TUG
**Mafi et al. (2019) **[65]	68.63 ± 2.86	62/0	Resistance exercise, epicatechin	Nutrition, RT	Moderate to vigorous	Placebo capsules and no exercise	2 months	ASMI, leg extension strength, TUG
**Nabuco et al. (2019) **[66]	Protein: 68.0 ± 4.2 Placebo: 70.1 ± 3.9	0/26	Whey protein and resistance exercise	Nutrition, RT	Moderate	Resistance exercise	3 months	Body composition, gait speed, 5 time chair stand, leg strength
**Vikberg et al. (2019) **[67]	70.9 ± 0.03	32/38	Resistance training	RT	Moderate to vigorous	No exercise intervention	10 weeks	Body composition, SPPB, TUG, chair sit-stand time
**Yamada et al. (2019) **[68]	84.2 ± 5.5	39/73	The combined resistance exercise and nutritional supplementation group, the exercise alone group, the nutritional supplementation alone group	Nutrition, AT, RT	Mild	Usual care	12 weeks	Measurement of echo intensity in thigh muscle, knee extension torque, skeletal muscle mass and phase angle, physical function
**Zhu et al. (2019a) **[69]	Control: 72.2 ± 6.6 Exercise: 74.5 ± 7.1 Exercise and nutrition: 74.8 ± 6.9	26/87	Mixed aerobic and resistance exercise, nutrition supplement	Nutrition, AT, RT	Mild	Maintain usual diet and activities	3 months	Body composition, gait speed, quality of life (SF-12 physical component score)
**Zhu et al. (2019b) **[70]	Taichi: 88.8 ± 3.7 Whole body vibration: 89.5 ± 4.4 Control: 87.5 ± 3.0	90/0	Taichi, whole body vibration	Taichi, vibration	–	Maintain usual diet and activities	2 months	Handgrip strength, lower limb strength, 6-m gait speed test, TUG, 5 s sit to stand test
**Bagheri et al. (2020) **[71]	E + R: 64.1 ± 3.3 R + E: 63.8 ± 3.6 C: 65 ± 3.9	30/0	Resistance training, endurance training, Resistance training plus endurance training	AT, RT	Vigorous	Control	8 weeks	Upper body strength, lower body strength
**Björkman et al. (2020) **[72]	Protein: 83.6 ± 4.7 Iso-caloric placebo: 84.0 ± 3.9 Control: 83.7 ± 5.1	70/148	Protein and vitamin D supplement, home based aerobic exercise	Nutrition, AT	–	Home based aerobic exercise	12 months	Handgrip strength, SPPB
**Chang et al. (2020) **[73]	79.3 ± 5.1	0/40	Elastic band resistance training	RT	Mild	No resistance exercise	3 months	SMI, handgrip strength, leg strength, gait speed
**Liao et al. (2020) **[74]	70.9 ± 7.3	0/40	Elastic band resistance training	RT	Moderate to vigorous	Health education	3 months	Body composition, gait speed, 30-s chair stand test
**Oh et al. (2020) **[75]	Antigravity treadmill: 76.94 ± 9.43 Conventional rehab: 81.15 ± 4.90	12/26	Antigravity treadmill and standard rehabilitation exercises	Rehabilitation exercise	–	Standard rehabilitation exercises	10 days	Handgrip strength, quality of life (EQ-5D)
**Rooks et al. (2020) **[76]	Bimagrumab: 79.5 ± 5.46 Placebo: 78.3 ± 5.03	71/109	Bimagrumab	Drug	–	Placebo	7 months	Body composition, SPPB, handgrip strength, 6-min walk distance, gait speed
**Espinoza et al. (2021) **[77]	67.5 ± 5.4	6/15	Intranasal oxytocin	Drug	–	Intranasal saline	2 months	Body composition, SPPB, TUG
**Lee et al. (2021) **[78]	Exercise: 70.13 ± 4.41 Control: 71.82 ± 5.23	0/27	Resistance exercise	RT	Moderate	Health education	3 months	Body composition, ASMI, handgrip strength, gait speed, TUG, 30-s chair stand test
**Li et al. (2021) **[79]	Nutrition: 70.04 ± 3.98 Exercise: 73.73 ± 5.69 Nutrition + Exercise: 71.52 ± 5.28 Control: 72.91 ± 6.29	70/99	Protein, vitamin D, and fish oil supplementation, mixed aerobic and resistance exercise	Nutrition, AT, RT	Vigorous	Routine consultation	3 months	Body composition, handgrip strength
**Nasimi et al. (2021) **[80]	Intervention: 71.0 (69.0, 73.5)^a^ Control: 69.0 (66.0, 75.5)^a^	50/16	Yogurt fortified with beta-Hydroxy beta- Methyl Butyrate (HMB) and vitamins D and C	Nutrition	–	Plain yogurt	3 months	Body composition, handgrip strength, gait speed
**Osuka et al. (2021) **[81]	72.1 ± 4.3	0/156	β-hydroxy-β-methylbutyrate supplementation and resistance exercise	Nutrition, RT	Moderate to vigorous	Health education and placebo supplement	3 months	Body composition, handgrip strength, gait speed, TUG, 5-repetition sit-to-stand time
**Seo et al. (2021) **[82]	Exercise: 70.3 ± 5.38 Control: 72.9 ± 4.75	0/22	Resistance exercise	RT	Moderate to vigorous	No exercise intervention	4 months	Body composition, handgrip strength, gait speed
**Lace study group et al. (2022) **[83]	Perindopril/leucine:78.1 ± 5.5 Perindopril:79.5 ± 6.6 Leucine:78.6 ± 6.5 Placebo: 79 ± 5.7	67/78	Perindopril 4 mg, or oral leucine powder 2.5 g thrice daily	Drug	–	Placebo	12 months	SPPB, maximum grip strength, maximum quadriceps strength, six-minute walk distance, four metre walk speed, five times sit to stand test, instrumental activities of daily living, ASMI, quality of life (EQ5D-3 level version)

*ASM* Appendicular skeletal muscle mass, *ASMI* Appendicular muscle mass index, *SMI* Total skeletal muscle mass index, *SPPB* Short Physical Performance Battery, *TUG* Timed up and go test, *EQ-5D* EuroQol five-dimensional questionnaire, *SF-36* 36-item Short-Form Survey, *SF-12* 12-item Short Form Survey

^a^Median (25th interquartile range, 75th interquartile range)

Regarding sarcopenia outcome measures, 23 studies examined muscle mass, 26 studies examined handgrip strength (including one study on upper body strength), 16 studies examined lower leg strength, 23 studies examined gait speed, and 25 studies examined physical performance, including TUG, SPPB, single leg stance, and chair stand tests. Network plots of trials with each outcome are shown in Fig S1a-1 m.

## Results of network meta-analysis

### Physical performance

#### 5 times sit to stand

Studies measuring this outcome included a total of 108 subjects receiving RT, 40 subjects receiving AT + RT, 314 subjects receiving nutritional interventions, 139 subjects receiving RT and nutritional interventions, 36 subjects receiving AT + RT and nutritional interventions, 48 subjects receiving whole body EMS, 24 subjects practicing Taichi, 115 subjects receiving Bimagrumab, and 522 subjects in the control group (Table 1). The results showed that the time taken to complete 5TSTS decreased significantly in the RT group (mean difference[MD]: -1.59 s; 95% CrI: -2.78, -0.44; SUCRA = 68.7%), RT + nutritional intervention group (MD:-1.57 s; 95% CrI: -2.57, -0.48; SUCRA = 67.8%), and AT + RT + nutritional intervention group (MD:-2.28 s; 95% CrI: -4.23, -0.29; SUCRA = 84.9%) (Fig S2a, S3a). A favorable trend was found in AT + RT group but did not reach statistical significance (Fig S2a).

**Fig. 2:**
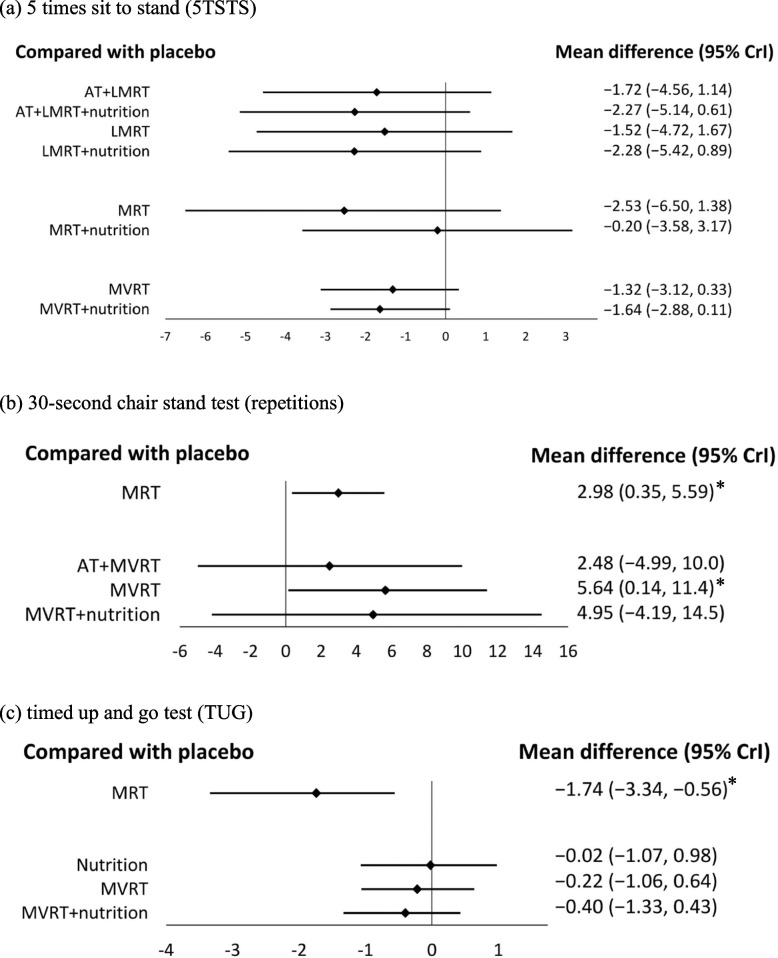

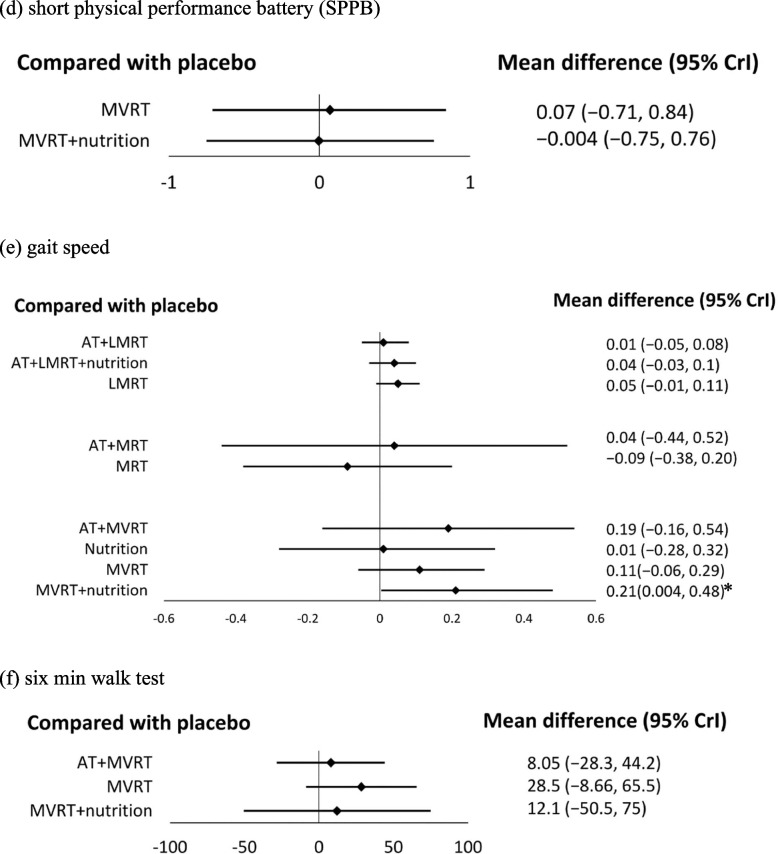
Forest plot comparing the effects of LMRT, MRT, and MVRT on physical performance. An asterisk denotes statistical significance. **a** 5 times sit to stand (5TSTS). **b** 30-s chair stand test (repetitions). **c** timed up and go test (TUG). **d** short physical performance battery (SPPB). **e** gait speed. **f** six min walk test

Regarding RT intensity, neither LMRT, MRT nor MVRT was associated with improvement in 5TSTS (Fig. 2a). In the subgroup analysis, no differences were found for all interventions done on women and participants with low muscle mass.

#### 30-s chair stand test (repetitions)

A total of 95, 25, 15, and 93 older adults in the RT, AT + RT, RT + nutrition, and placebo group, respectively, were included for comparison (Table 1). RT had the greatest effect (SUCRA = 79.15%) on the number of repetitions done in the 30-s chair stand test with a MD of 3.72 (95% CrI: 1.23, 7.31) (Fig S2b, S3b). Regarding RT intensity, MRT (MD:2.98; 95% CrI:0.35, 5.59; SUCRA = 98.32%) and MVRT (MD:5.64; 95% CrI:0.14, 11.4; SUCRA = 81.19%) were both associated with dose-responsive improvement in the 30-s chair stand test (Fig. 2b).

In the subgroup analysis, RT remained effective in women, resulting in a MD of 3.69 (95% CrI: 1.22, 7.27; SUCRA = 85.94%).

#### Timed up and go test

A total of 143 older adults using RT, 86 adults using nutrition supplements, 98 adults using RT + nutrition, 12 adults using oxytocin, 28 adults practicing Taichi, 44 adults using whole body EMS, and 194 adults in the placebo group were included for comparison (Table 1). RT demonstrated greater improvement than other interventions (SUCRA = 65.52%) on TUG with a MD of -0.85 (95% CrI: -1.69, -0.1) (Fig S2c, S3c). The effect was mainly attributable to MRT (MD: -1.74; 95% CrI: -3.34, -0.56; SUCRA = 99.18%) instead of MVRT (Fig. 2c).

There were no differences in subgroup analysis on TUG among all interventions done on women, men, the low muscle mass group, and the sarcopenic obesity group.

#### Short physical performance battery (SPPB)

We included 391 (placebo), 29 (RT), 16 (AT + RT), 289 (nutrition), 73 (AT + nutrition), 51 (RT + nutrition), 66 (MK-0773), and 12 (oxytocin) older adults for comparison (Table 1). No significant differences were found among all interventions in all participants (Fig S2d, S3d), MVRT group (Fig. 2d) and women.

#### Gait speed

A total of 227 participants in the RT group, 81 participants in the AT + RT group, 465 participants in the nutritional intervention group, 62 participants in the RT + nutrition group, 36 participants in the AT + RT + nutrition group, 106 participants in the whole body EMS group, 24 participants in the Taichi group, 115 participants in the Bimagrumab group, 25 participants in the whole body EMS + nutrition group, 66 participants in the MK-0773 group, and 794 participants in the placebo group were included for comparison (Table 1). RT + nutrition increased GS with a MD of 0.17 (95% CrI: 0.01, 0.34) (SUCRA = 85.6%) (Fig S2e, S3e). Regarding RT intensity, MVRT + nutrition was associated with improvement in GS (MD:0.21; 95% CrI: 0.003, 0.48; SUCRA = 84.87%), but LMRT and MRT produced no significant differences (Fig. 2e).

There was no improvement on GS among all interventions in women, those with dynapenia, and those with sarcopenic obesity.

#### Six min walk test

We included 27 adults from the RT group, 65 adults from the AT + RT group, 33 adults from the nutrition group, 15 adults from the RT + nutrition group, 36 adults from the AT + RT + nutrition group, 115 adults from the Bimagrumab group, and 182 adults from the placebo group for analysis (Table 1). No significant differences were found in each group compared to placebo (Fig S2f, S3f). There were also no significant effects of any interventions in the MVRT group (Fig. 2f).

### Muscle mass

#### Appendicular skeletal muscle index (ASMI)

**Fig. 3:**
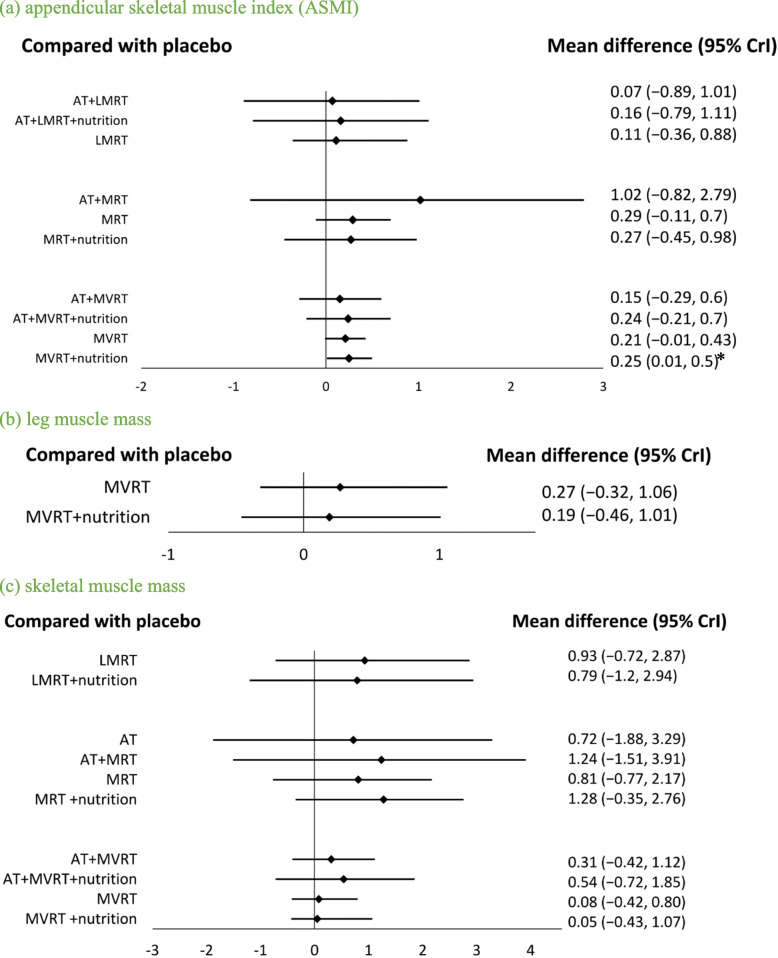
Forest plot comparing the effects of LMRT, MRT, and MVRT on muscle mass. An asterisk denotes statistical significance. **a** appendicular skeletal muscle index (ASMI). **b** leg muscle mass. **c** skeletal muscle mass

Twenty-four adults in the AT group, 305 adults in the RT group, 127 adults in the AT + RT group, 271 adults in the nutrition group, 175 adults in the RT + nutrition group, 95 adults in the AT + RT + nutrition group, 25 adults in the Whole body EMS group, 23 adults in the electrical acupuncture group, 58 adults in the whole body EMS + nutrition group, and 608 adults in the placebo group were compared (Table 1). RT + nutrition (MD:0.24; 95% CrI: 0.1, 0.38; SUCRA = 78.67%), RT (MD:0.19; 95% CrI: 0.08, 0.3; SUCRA = 65.74%), and nutrition (MD:0.15; 95% CrI: 0.04, 0.26; SUCRA = 53.49%) significantly increased ASMI (Fig S2g, S3g). Although LMRT and MRT were not associated with changes in ASMI, MVRT + nutrition was positively associated with increased ASMI (MD:0.25; 95% CrI: 0.01, 0.5; SUCRA = 68.21%) (Fig. 3a).

None of the interventions influenced ASMI in men, women, low muscle mass group, and sarcopenic obesity group.

#### Leg muscle mass

A total of 97 participants in the RT group, 40 participants in the AT + RT group, 142 participants in the nutrition group, 90 participants in the RT + nutrition group, 36 participants in the whole body EMS group, and 272 participants in the placebo group were included for comparing leg muscle mass (Table 1). No significant differences were found in each pairwise comparison with placebo among all participants and the MVRT group (Fig S2h, S3h, and Fig. 3b).

#### Skeletal muscle mass

Studies with skeletal muscle mass as an outcome included 24 participants in the AT group, 298 participants in the RT group, 122 participants in the AT + RT group, 280 participants in the nutrition group, 243 participants in the RT + nutrition group, 59 participants in the AT + RT + nutrition group, 61 participants in the whole body EMS group, 24 participants in the Taichi group, 115 participants in the Bimagrumab group, 65 participants in the MK-0773 group, 12 participants in the oxytocin group, and 655 participants in the placebo group (Table 1). Bimagruab (MD:1.94; 95% CrI: 0.81, 3.07; SUCRA = 91.21%), MK-0773 (MD:1.37; 95% CrI: 0.37, 2.40; SUCRA = 79.58%), RT + nutrition (MD:0.82; 95% CrI: 0.31, 1.32; SUCRA = 57.05%), RT (MD:0.58; 95% CrI: 0.11, 1.04; SUCRA = 41.72%), and nutrition (MD:0.51; 95% CrI: 0.06, 0.97; SUCRA = 36.73%) increased skeletal muscle mass significantly (Fig S2i, S3i). However, LMRT, MRT, and MVRT were not associated with enhanced skeletal muscle mass (Fig. 3c).

### Muscle strength

#### Handgrip strength

We included 43 older adults in the AT group, 267 older adults in the RT group, 152 older adults in the AT + RT group, 553 older adults in the nutrition group, 73 older adults in the AT + nutrition group, 172 older adults in the RT + nutrition group, 95 older adults in the AT + RT + nutrition group, 53 older adults in the whole body EMS group, 24 older adults in the Taichi group, 58 older adults in the whole body EMS + nutrition group, and 1,063 older adults in the placebo group for comparison (Table 1). AT + RT + nutrition (MD:3.25; 95% CrI: 1.12, 5.4; SUCRA = 87.12%), AT + RT(MD:2.43; 95% CrI: 0.69, 4.11; SUCRA = 87.12%), RT + nutrition (MD:2.48; 95% CrI: 0.98, 4; SUCRA = 73.27%), AT + RT (MD:2.43; 95% CrI: 0.69, 4.11; SUCRA = 70.45%), RT (MD:2.21; 95% CrI: 1.18, 3.34; SUCRA = 66%), and nutrition (MD:1.73; 95% CrI: 0.76, 2.74; SUCRA = 50.33%) significantly improved handgrip strength (Fig S2j, S3j).

**Fig. 4:**
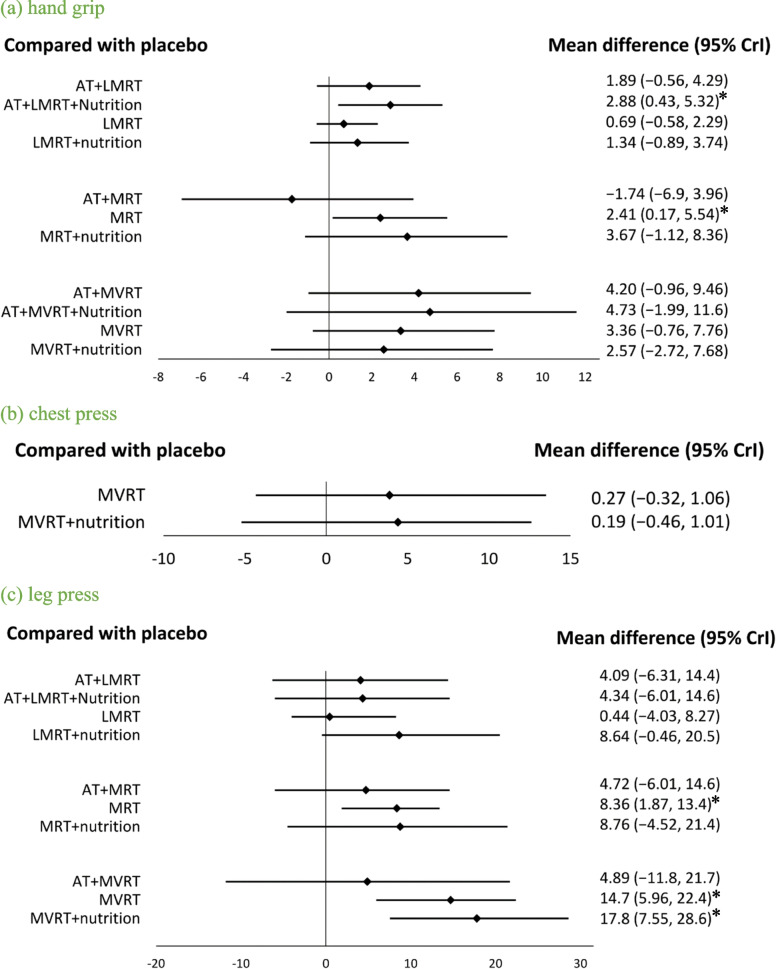
Forest plot comparing the effects of LMRT, MRT, and MVRT on muscle strength. An asterisk denotes statistical significance. **a** hand grip. **b** chest press. **c** leg press

For RT intensity, AT + LMRT + nutrition (MD:2.88; 95% CrI: 0.43, 5.32; SUCRA = 93.01%) and MRT (MD:2.44; 95% CrI: 0.03, 5.70; SUCRA = 77.87%) were positively associated with gains in HG (Fig. 4a), whereas MVRT was not associated with changes in HG.

For women, RT was associated with HG improvement (MD:1.97; 95% CrI: 0.2, 4.12; SUCRA = 62.5%). There was no influence on HG among all interventions in men, low muscle mass group, and sarcopenic obesity group.

#### Chest press

A total of 24 adults in the RT group, 17 adults in the nutrition group, 36 adults in the RT + nutrition group, and 29 adults in the placebo group were included for comparing changes in chest press strength (Table 1). No significant differences among interventions were found in all participants and the MVRT group (Fig S2k, S3k, Fig. 4b).

#### Leg press

Studies examining leg press strength included a total of 43 older adults in the AT group, 267 older adults in the RT group, 152 older adults in the AT + RT group, 553 older adults in the nutrition group, 73 older adults in the AT + nutrition group, 172 older adults in the RT + nutrition group, 36 older adults in the RT + nutrition group, 95 older adults in the AT + RT + nutrition group, 53 older adults in the whole body EMS group, 24 older adults in the Taichi group, 58 older adults in the whole body EMS + nutrition group, and 1,063 older adults in the placebo group (Table 1). RT + nutrition (MD:12.3; 95% CrI: 5.59, 18.9; SUCRA = 88.33%) and RT (MD:8.24; 95% CrI: 3.78, 12.7; SUCRA = 70.81%) were significantly associated with gains in leg press strength (Fig S2l, S3l).

With respect to RT intensity, MRT (MD:8.36; 95% CrI: 1.87, 13.4; SUCRA = 80.5%) was positively associated with improvements in leg press strength. MVRT (MD:14.7; 95% CrI: 5.96, 22.4; SUCRA = 77.59%) and MVRT + nutrition (MD:17.8; 95% CrI: 7.55, 28.6; SUCRA = 89.71%) demonstrated even greater benefits in leg press strength (Fig. 4c). There was no influence on leg press among all interventions in subgroup analysis of men, women, and sarcopenic obesity group.

### Quality of life

**Fig. 5:**
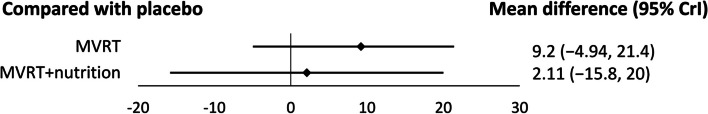
Forest plot comparing the effects of MVRT on physical quality of life

In total, 19 participants in the AT group, 34 participants in the RT group, 33 participants in the nutrition group, and 76 participants in the placebo group were included for comparing overall QOL. For comparing physical QOL, we examined 75 participants in the AT group, 67 participants in the RT group, 40 participants in the AT + RT group, 63 participants in the nutrition group, 74 participants in the AT + nutrition group, 69 participants in the RT + nutrition group, 36 participants in the AT + RT + nutrition group, and 215 participants in the placebo group. Additionally, 40 participants in the AT + RT group, 63 participants in the nutrition group, 69 participants in the RT + nutrition group, 36 participants in the AT + RT + nutrition group, and 159 participants in the placebo group were compared for psychological QOL (Table 1). No significant differences among interventions were found in all participants and the MVRT group (Fig S2m, S3m, Fig. 5).

### Sensitivity analysis

When reanalyzing data with the sequential exclusion of each study, the results remained consistent with the original analysis. Additionally, after excluding four studies that were assessed to have a high risk of bias assessed using ROB2 [35, 44, 69, 70], the rankings remained unchanged. Node-splitting model showed no inconsistency between direct and indirect comparisons (Table S3a-S3j). Egger’s test revealed no obvious publication bias except in studies involving leg muscle mass, HG, leg press, and QOL (Table S4). After removing a study done on patients with Alzheimer’s disease [73], the intervention effects were consistent except that RT + nutrition lost its significant effect on GS and HG, whereas whole body EMS + nutrition lost its significant effect on HG. After removing two studies involving post-operative patients [74, 75], the results were consistent except that RT + nutrition lost its significant effect on GS and HG, and whole body EMS + nutrition lost its significant effect on HG.

## Discussion

For community-dwelling older adults with sarcopenia, our systematic review and network meta-analysis confirmed that a combination of exercise and nutrition were associated with improved sarcopenia parameters, including 5TSTS, 30-s chair stand test (repetitions), TUG, GS, ASMI, skeletal muscle mass, HG, and leg press. Regarding RT intensity, LMRT only demonstrated desired effects on HG. MRT provided improvements in the 30-s chair stand test, TUG, HG, and leg press. MVRT had additional benefits on the 30-s chair stand test, GS, ASMI, and leg press.

Our study is the first network meta-analysis to investigate exercise effects on patients with sarcopenia according to RT intensity. The results revealed that boosting RT intensity to the moderate-vigorous level had significantly greater positive effects on physical function, lower body strength, and muscle mass. RT has been proven to be essential for the management of sarcopenia because it preferentially increases the cross-sectional area of type II muscle fibers that are replaced by slow type I muscle fibers and fatty tissue during the aging process [84–87]. Additionally, research has shown that adaptive responses including increasing myofibrillar protein synthesis, satellite cell count, myonuclei count, glycolytic function, mitochondrial volume, and mitochondrial protein synthesis in skeletal muscle occur following RT [88]. More importantly, relative RT intensity (%1RM) was associated with 18–35% of the variance for the muscle hypertrophy response [89]. More type II muscle units and associated muscle fibers were recruited with higher RT intensity in a dose–response manner, resulting in larger muscle size and greater force [90]. Therefore, with appropriate instruction and supervision before, during, and after exercise, moderate-to-vigorous RT intensity may be suggested for older adults with sarcopenia [11].

Surprisingly, MVRT was not associated with additional benefits compared to MRT in terms of TUG, which is a measure of overall functional mobility, including locomotion, static balance, and dynamic balance. Most MVRT trials increased intensity by elevating %1RM, but used the original exercise type, such as body weight workout and elastic band exercise, which mainly build limb strength. To improve agility and balance, power resistance training may provide benefits in addition to muscle power and physical performance. A 12-week RCT reported that high-speed RT program may bring greater improvement in walking sprint, 8-foot up-and-go test, and sit-to-stand test [91]. Another randomized within-subject trial demonstrated that power resistance training generated more increases in muscle power and movement velocity [92]. Considering the significance of rate-dependent mobility for fall prevention and functional maintenance in older adults [93, 94], velocity-based power training should be introduced and integrated into traditional RT programs.

According to our results, MVRT was not associated with greater gains in HG compared to MRT. Similarly, most MVRT programs tended to focus on reinforcing lower limb strength because gait and balance were more pertinent to all-cause mortality, activities of daily living (ADL) decline, and instrumental activities of daily living (IADL) worsening [95, 96]. Compared to gait and balance, HG has been proven to be equivalently essential in the concept of intrinsic capacity developed by the WHO [97]. In addition, grip strength was related to cognitive performance, mental health, and quality of life cross-sectionally and longitudinally [98], and grip training has been reported to improve cognitive function through increasing the local efficiency of brain white matter connectivity in minor acute ischemic stroke patients [99]. Because most RT programs target large muscle groups (e.g., chest press and squat), training focusing on handgrip strength, like the lateral pulldown and reverse fly, may be incorporated when intensifying exercise is warranted.

Management of sarcopenia based on strong evidence of treatment effectiveness is required. Our findings suggest that adding nutritional support to exercise interventions can amplify the effects on sarcopenia. Specifically, when nutrition is combined with RT, the improvements in outcome measures, such as HG and leg press, are more pronounced than with RT alone. Although electrical muscle stimulation, electrical acupuncture, whole body vibration, and Taichi have been introduced to manage sarcopenia, our pooled analysis showed no promising evidence of these interventions having favorable effects on sarcopenia parameters [9]. Novel agents such as bimagrumab (human monoclonal antibody targeting activin type 2 receptors) [100] and MK0733 (selective androgen receptor modulator) [36] have the potential to improve skeletal muscle mass, but our results show no benefit on muscle function and physical performance. Recent studies suggested that dysfunctions at the neuromuscular junction and within mitochondria may contribute to the deterioration of muscle function [101]. Engaging in physical activity has the potential to modify gene expression of acetylcholine receptor subunits and optimize mitochondrial dynamics, including fusion, fission, and autophagy, thereby supporting muscle function and preserving muscle strength [102]. Notably, sarcopenic patients with low muscle strength may benefit more from exercise interventions than those without strength deficits [103].

Future clinical trials are encouraged to investigate the impact of exercise intensity on sarcopenia outcomes, focusing on patients with low muscle strength and those with severe sarcopenia, characterized by reduced muscle mass, compromised physical performance, and diminished strength.

The strength of our study is the robust evidence from the network meta-analysis of currently available clinical trials. Indirect comparison allows for estimation of intervention effects even if there are no head-to-head trials. Similar results among direct and indirect comparisons reinforce and support our conclusions. However, this study also has several limitations. First, inconsistent sarcopenia criteria among studies compromised the generalizability of the findings. Second, heterogenous interventional protocols of exercise (e.g., exercise type, intensity, duration, and frequency) and diverse nutritional support might make clinical application difficult. Lack of detailed information about exercise protocols in some studies might lead to misclassification bias. Universal reporting of exercise interventions with FITT-VP (frequency, intensity, time, type, volume, and progression) information should be encouraged in future studies [11]. Third, many studies have failed to report on exercise adherence, potentially leading to an underestimation of the true effects of exercise interventions. Fourth, discordant advice on usual diet habits, lifestyle, and physical activity in control groups among studies might obscure the intervention effects.

Fifth, given the variety of metrics used to evaluate exercise, comparing results across different studies can be challenging. The ACSM recommends a holistic approach to evaluating exercise intensity, encompassing metrics such as 1-RM, VO2 max, and RPE. This approach offers both an objective measure and a subjective assessment of effort, streamlining the standardization of exercise intensity across various studies [11]. Detailed reporting on exercise intervention protocols should be emphasized in future studies.

## Conclusions

This network meta-analysis suggests that RT with or without nutritional supplementation improves physical performance, ASMI, and handgrip strength in older adults suffering from sarcopenia. Higher RT intensity potentially generates more benefits on lower body strength and muscle mass compared to lower RT intensity. Further investigation is necessary to clarify the advantages and disadvantages of intensifying RT and give insight to future exercise program modifications.

### Supplementary Information

**Additional file 1.**

## Data Availability

The datasets used and/or during the current study are available from the corresponding author on reasonable request.
