# Supplementary material for: Is moderate resistance training adequate for older adults with sarcopenia? A systematic review and network meta-analysis of RCTs
Source: Eur Rev Aging Phys Act. 2023 Nov 29;20:22. doi: 10.1186/s11556-023-00333-4 (PMC10687931; doi:10.1186/s11556-023-00333-4)
Supplement: Supplementary file 1 — Additional file 1. [file 11556_2023_333_MOESM1_ESM.docx]

Index of supplementary data

- Fig S1. Network plots of treatments
- Fig S2. Forest plots of treatments
- Fig S3. Ranking regarding treatment effectiveness assessed by the

surface under the cumulative ranking curve (SUCRA) values. (A larger SUCRA denoted a more effective treatment strategy)

- Table S1. Risk of bias assessment using ROB2
- Table S2. Details of baseline characteristics of included studies
- Table S3. Treatment comparisons using node-splitting model
- Table S4. Egger's test for publication bias
- S1. Search terms and search string

Fig S1a. Network plot of treatments on 5TSTS. Network plots visually represented the number of study participants according to the size of nodes and the number of trials conducted according to the thickness of connecting lines.


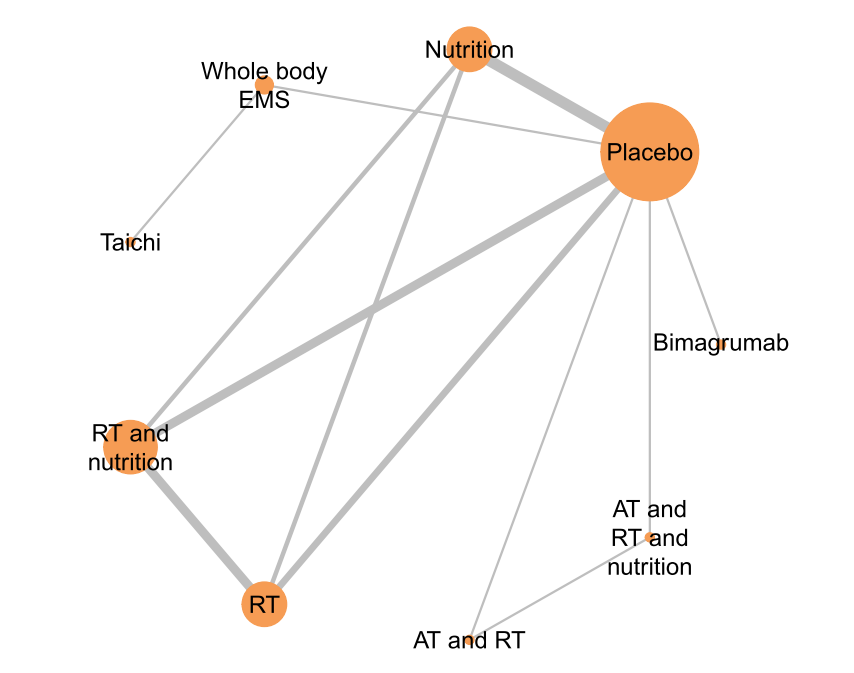


Fig S1b. Network plot of treatments on 30-second chair stand test


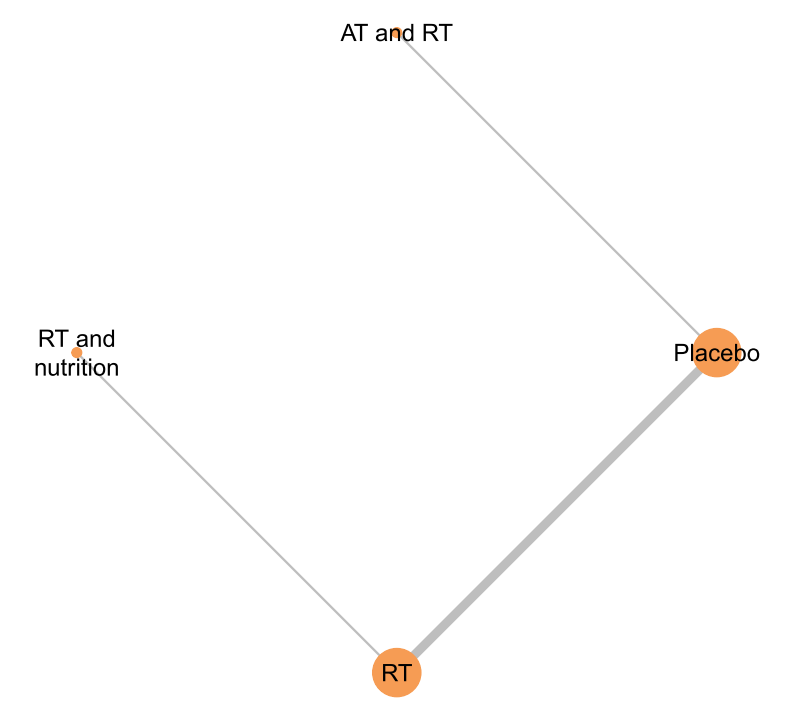


Fig S1c. Network plot of treatments on TUG


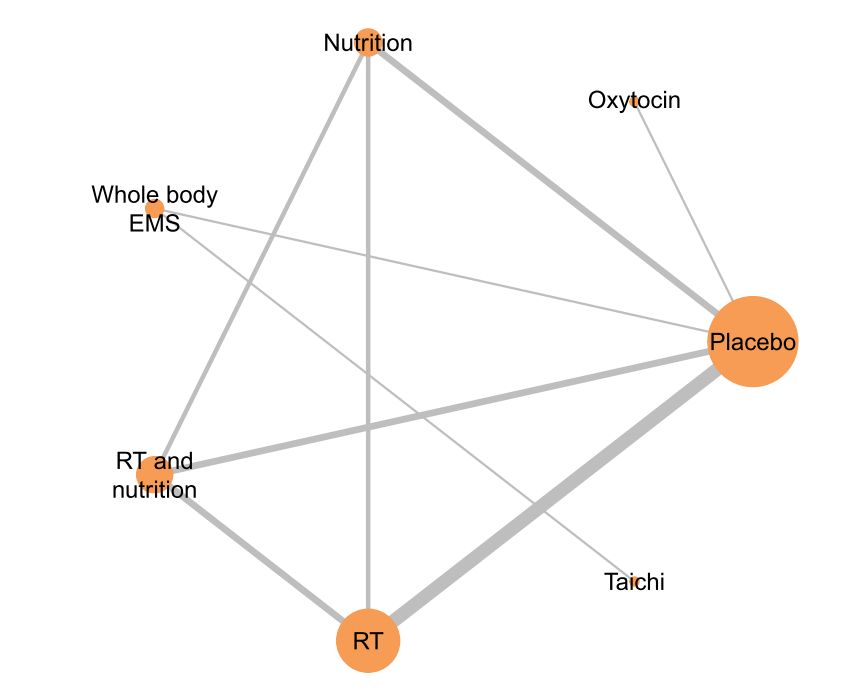


Fig S1d. Network plot of treatments on SPPB


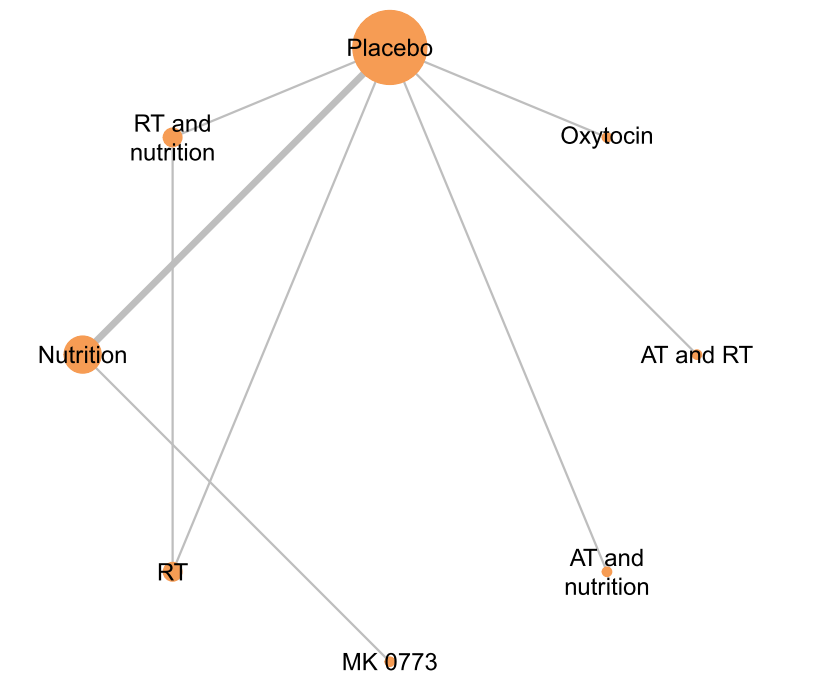


Fig S1e. Network plot of treatments on gait speed


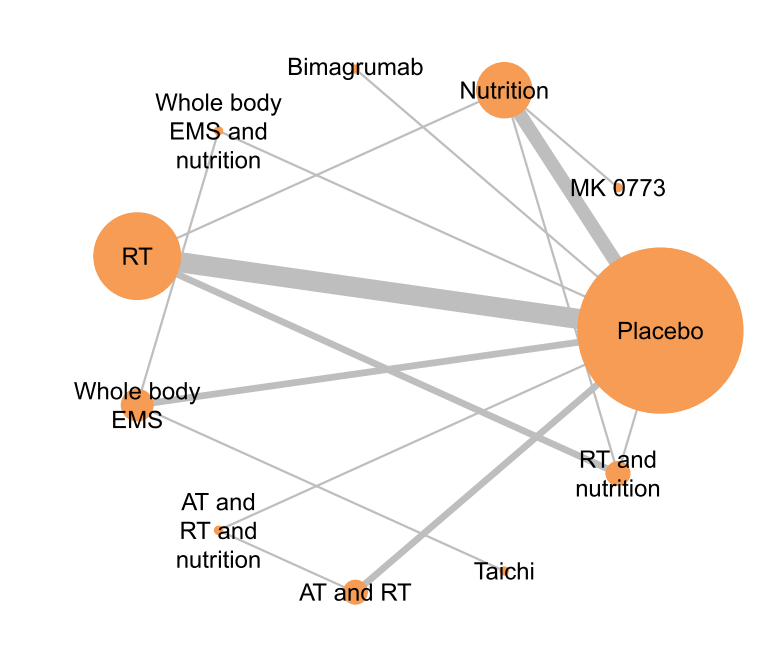


Fig S1f. Network plot of treatments on 6 min walk test


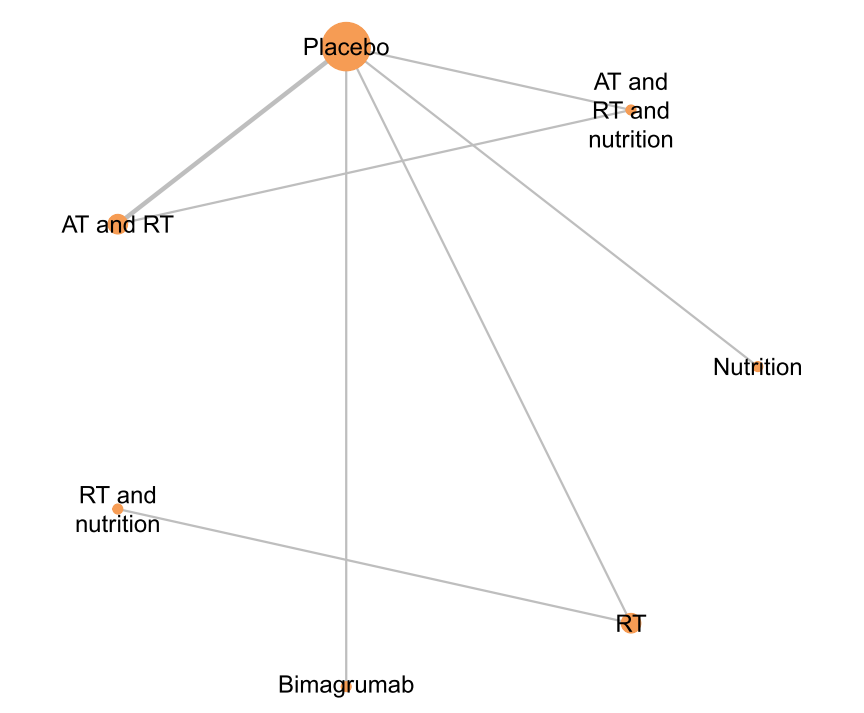


Fig S1g. Network plot of treatments on ASMI


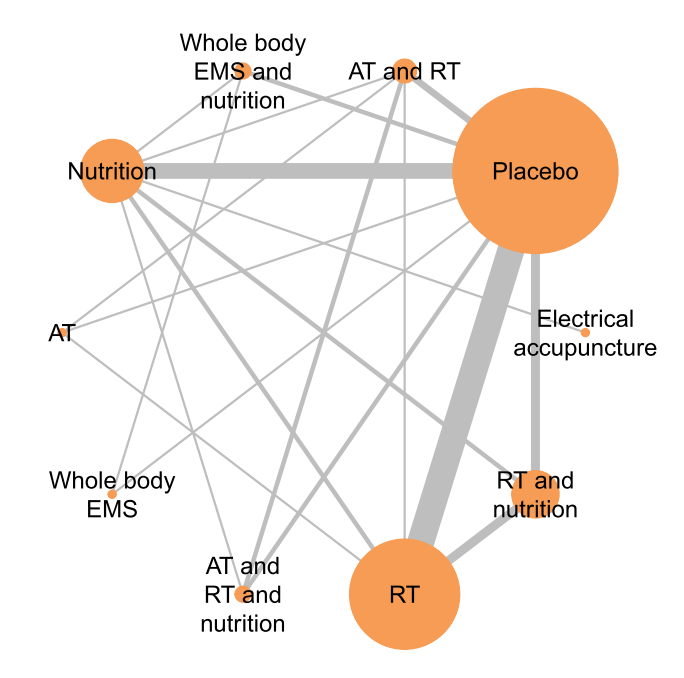


Fig S1h. Network plot of treatments on leg muscle mass


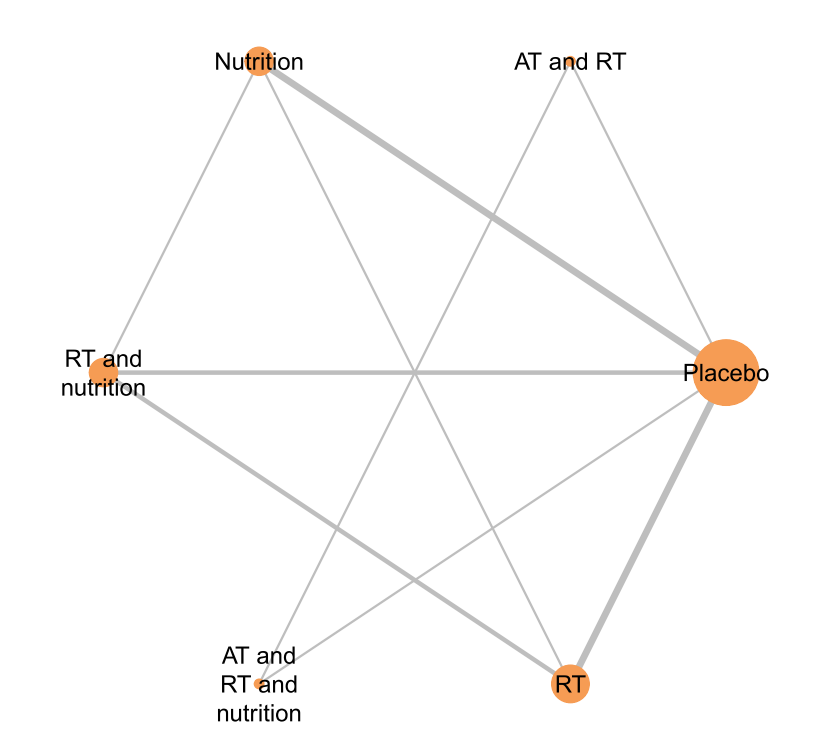


Fig S1i. Network plot of treatments on skeletal muscle mass


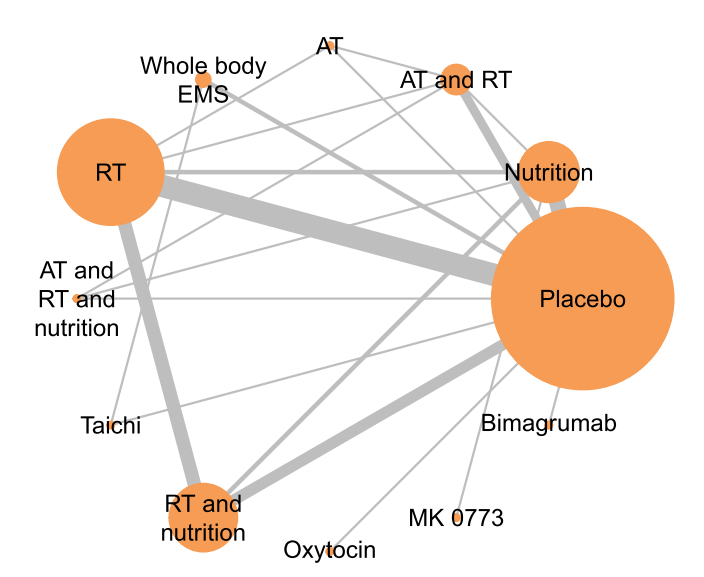


Fig S1j. Network plot of treatments on handgrip strength


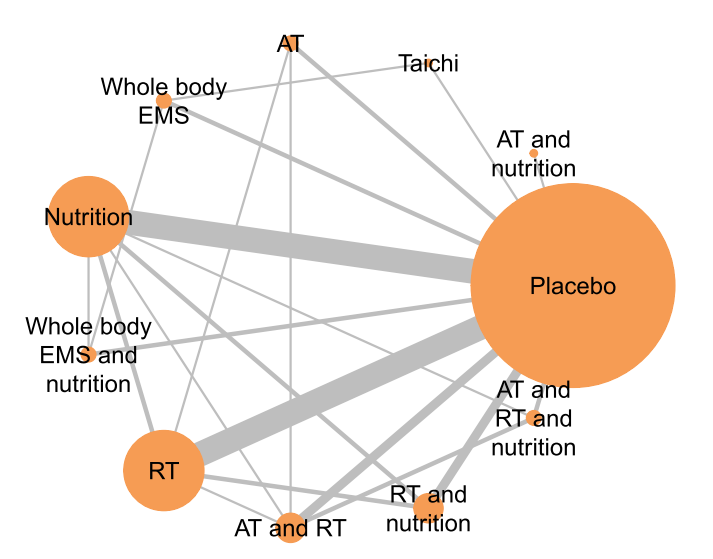


Fig S1k. Network plot of treatments on chest press


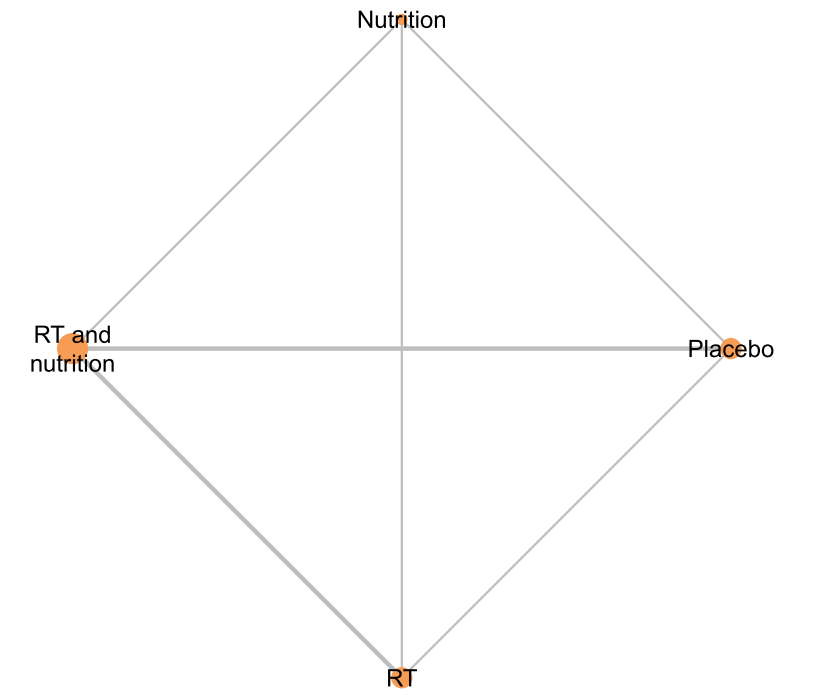


Fig S1l. Network plot of treatments on leg press


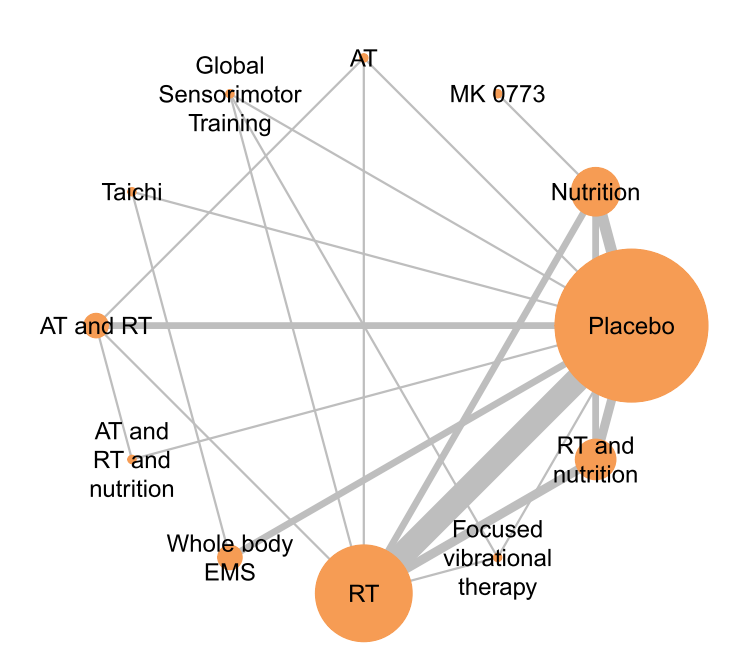


Fig S1m-1. Network plot of treatments on overall QOL


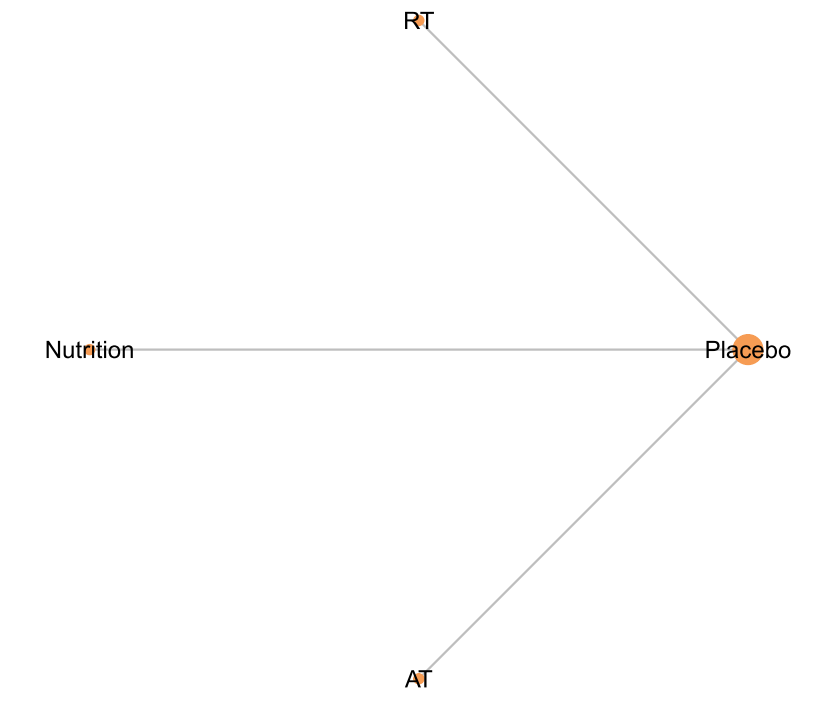


Fig S1m-2. Network plot of treatments on mental QOL


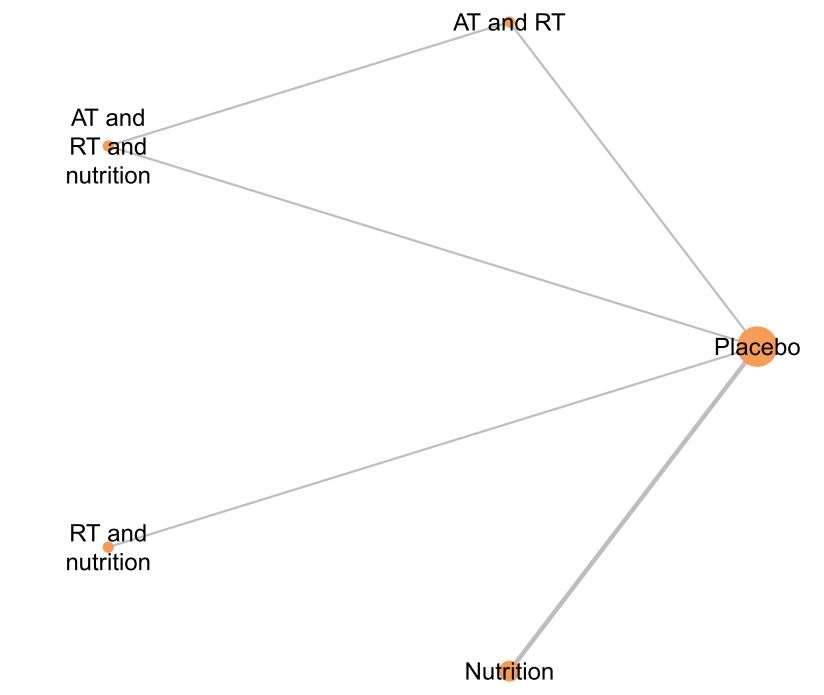


Fig S1m-3. Network plot of treatments on physical QOL


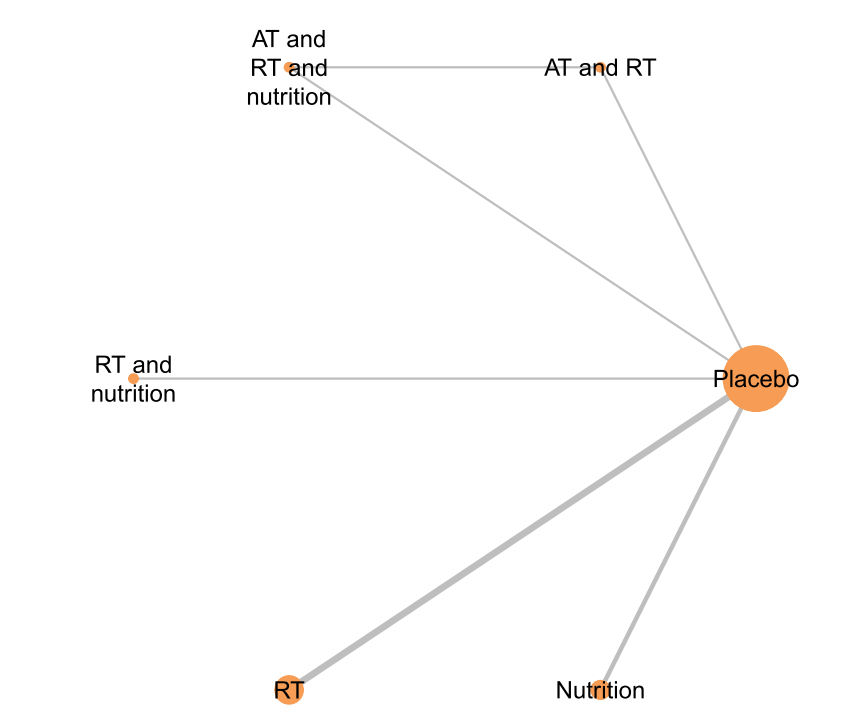


Fig S2a. Forest plot of treatments on 5TSTS


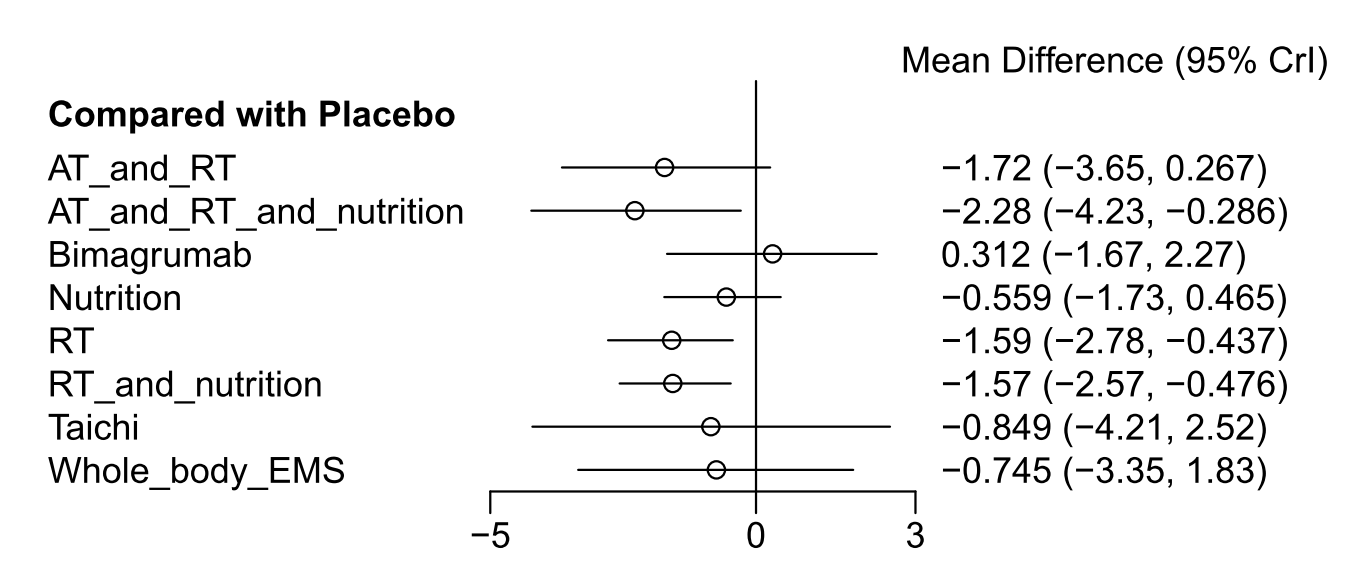


Fig S2b. Forest plot of treatments on 30-second chair stand test


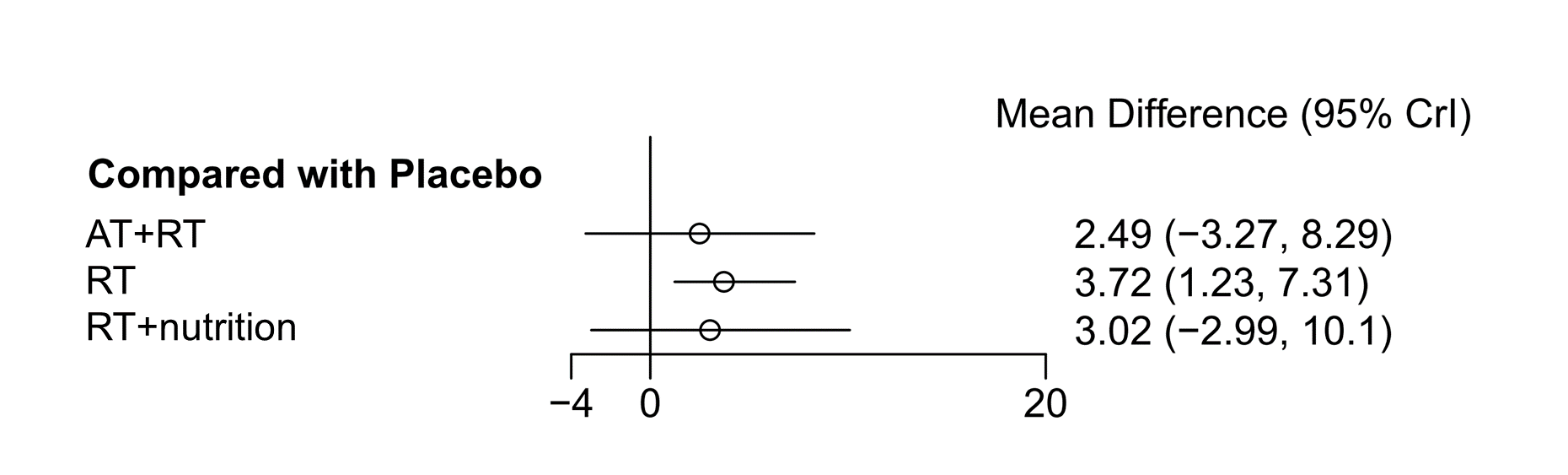


Fig S2c. Forest plot of treatments on TUG


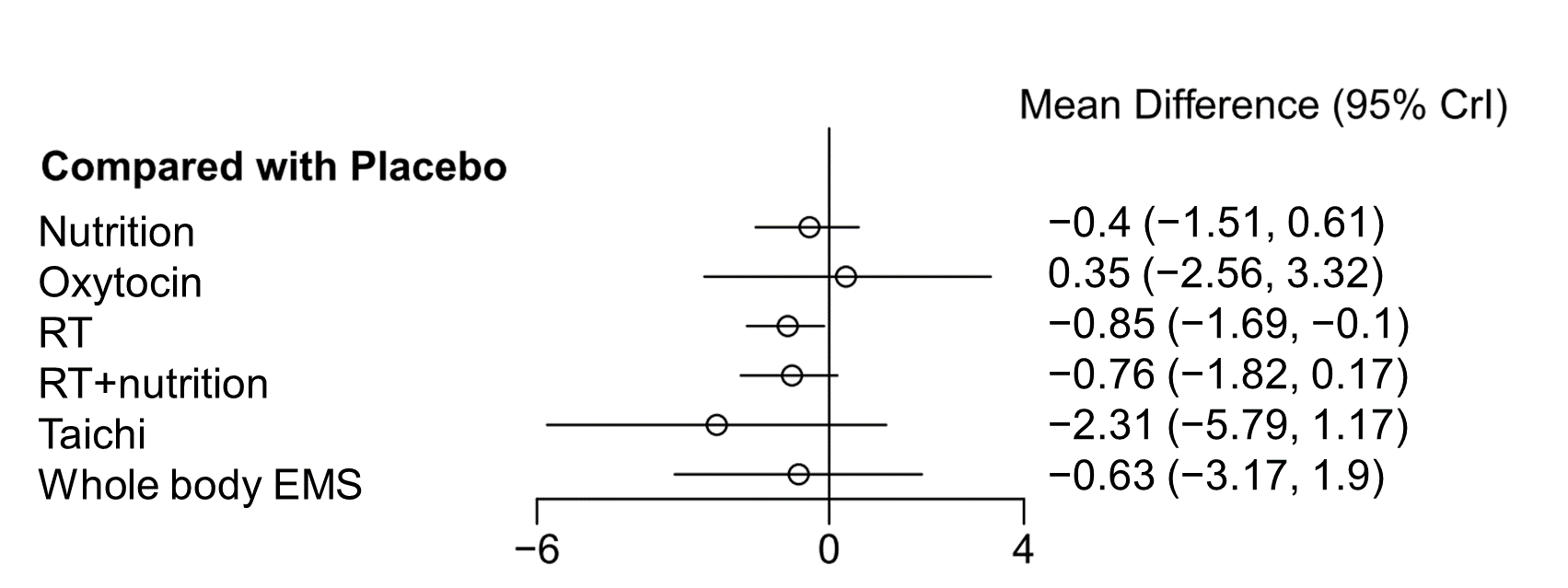


Fig S2d. Forest plot of treatments on SPPB


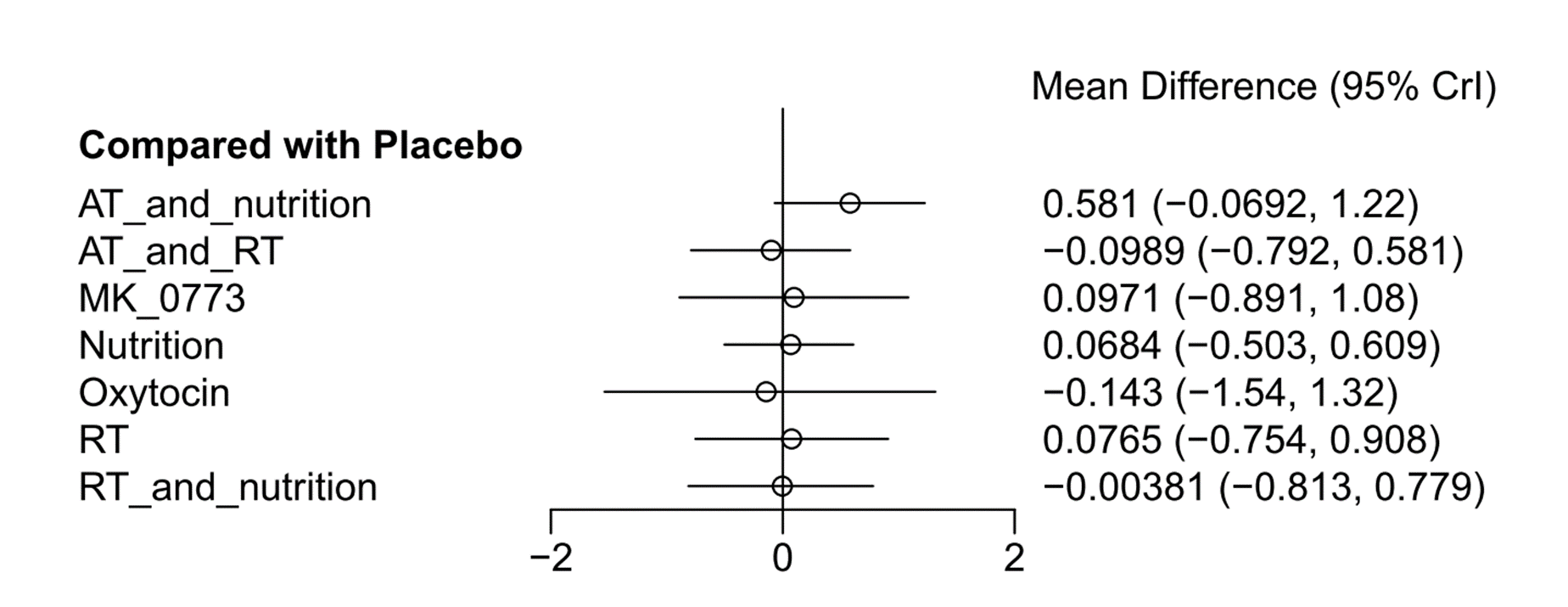


Fig S2e. Forest plot of treatments on gait speed


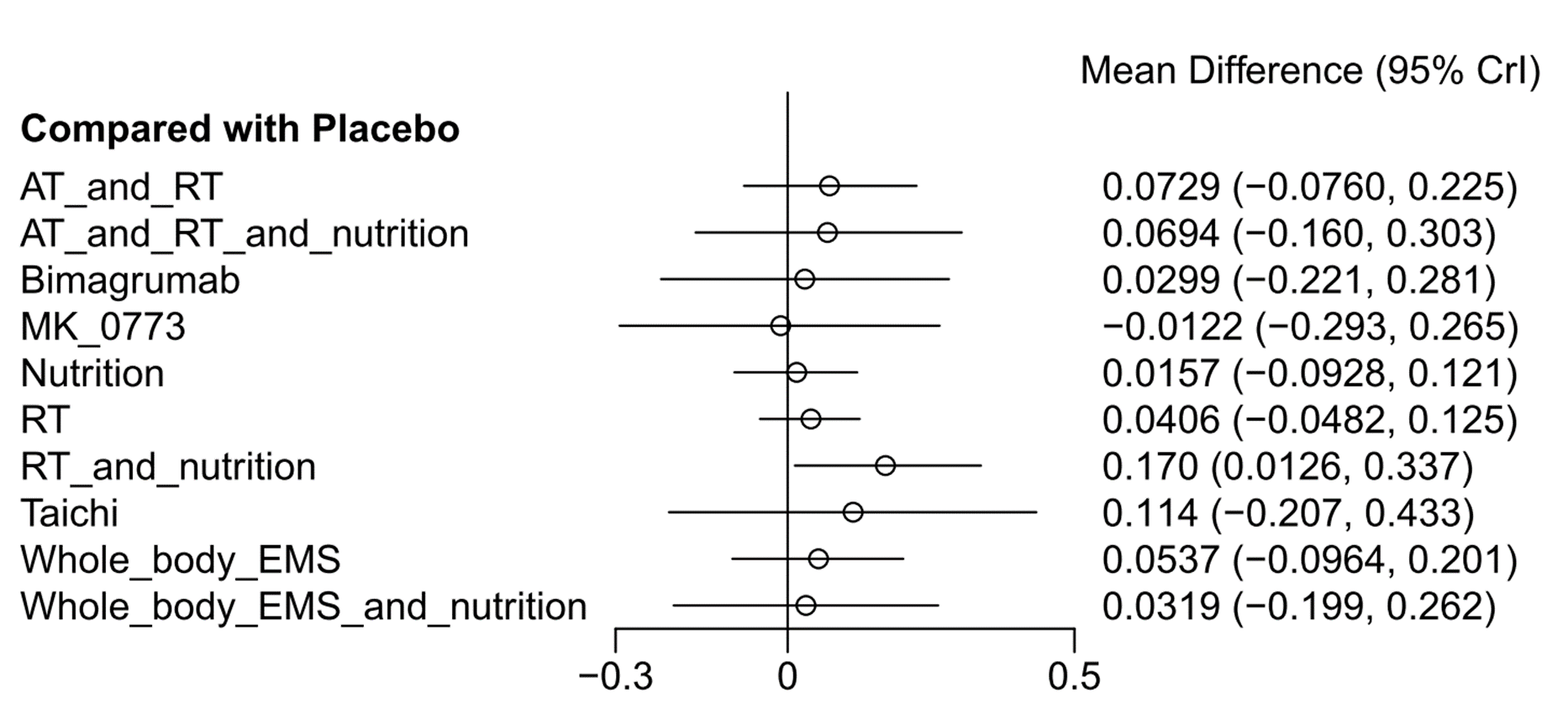


Fig S2f. Forest plot of treatments on 6 min walk test


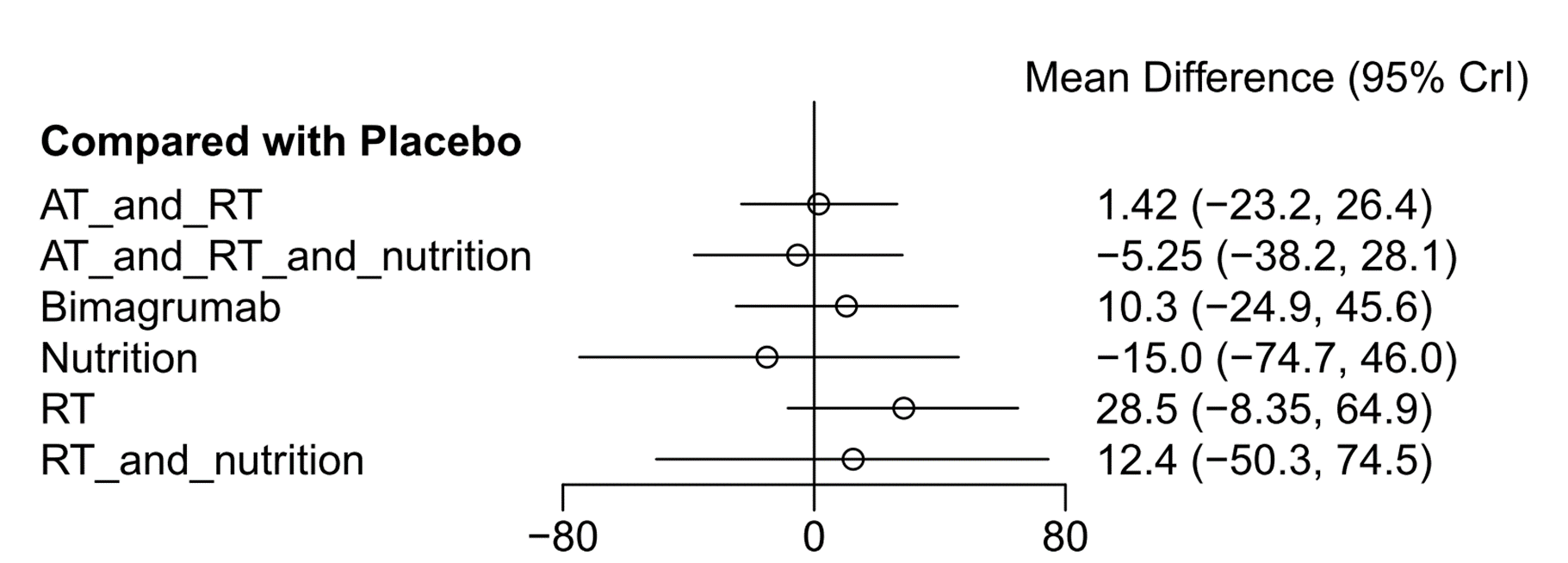


Fig S2g. Forest plot of treatments on ASMI


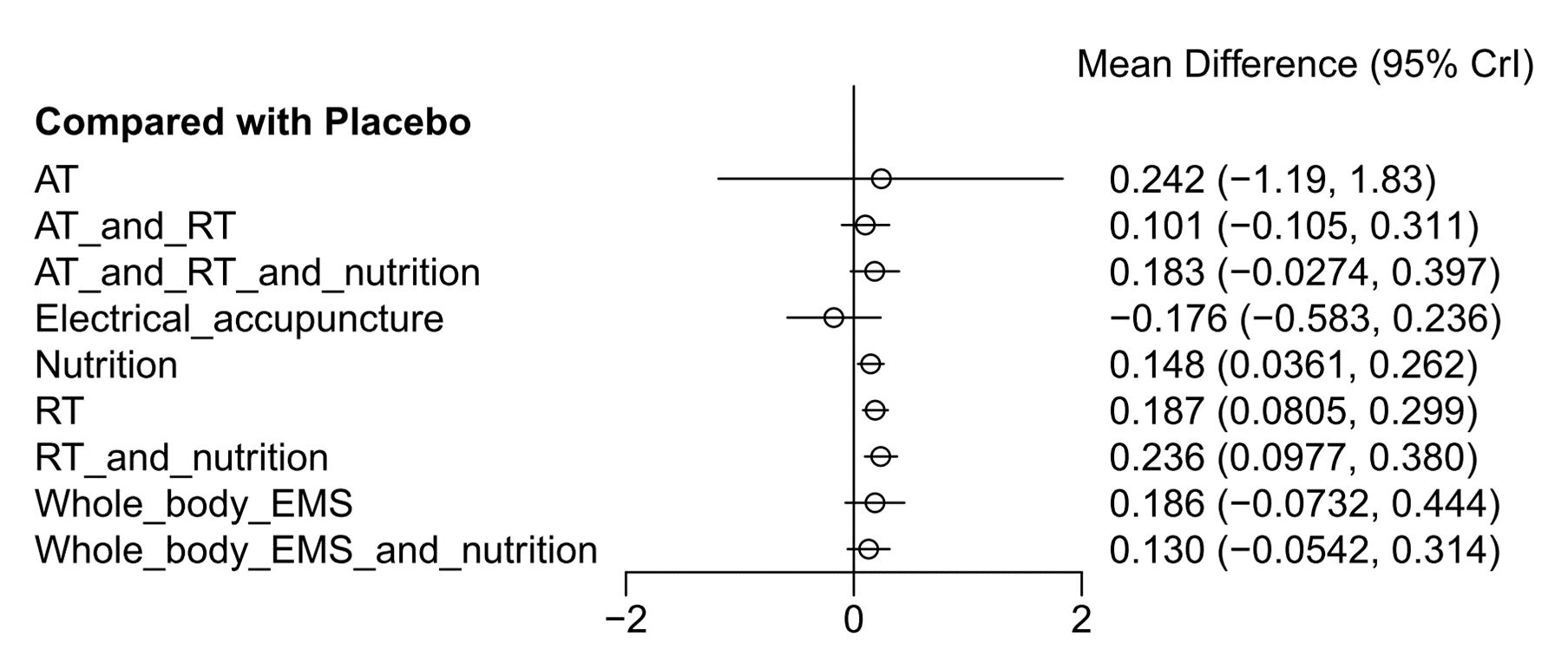


Fig S2h. Forest plot of treatments on leg muscle mass


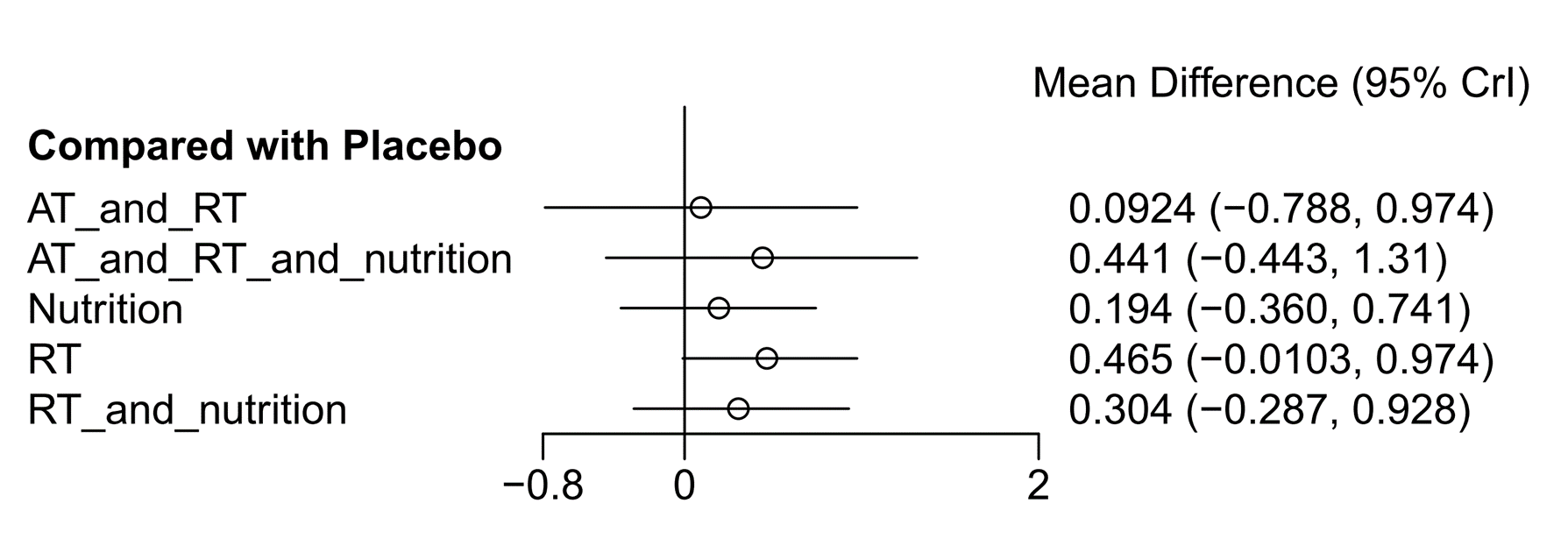


Fig S2i. Forest plot of treatments on skeletal muscle mass


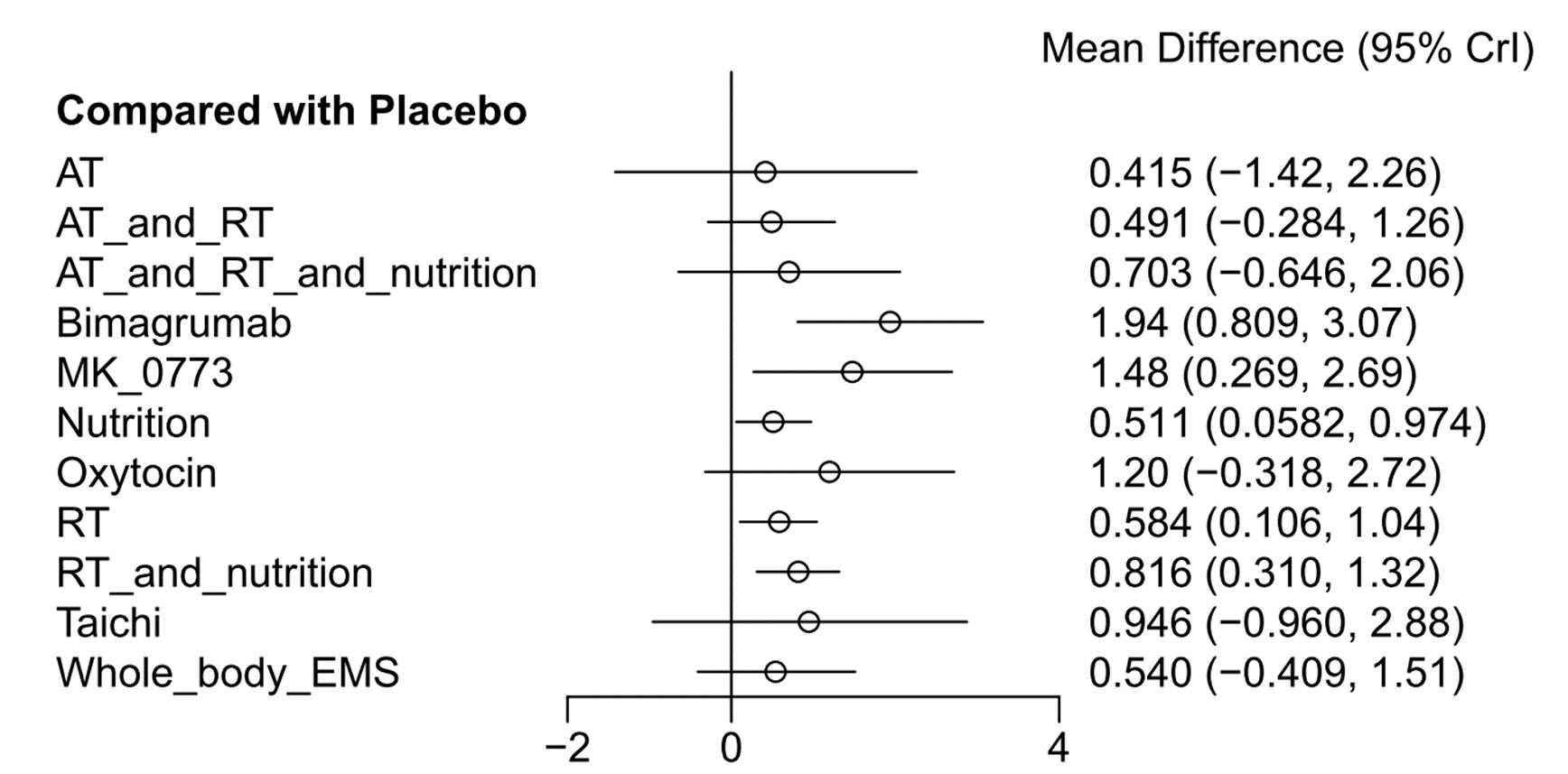


Fig S2j. Forest plot of treatments on handgrip strength


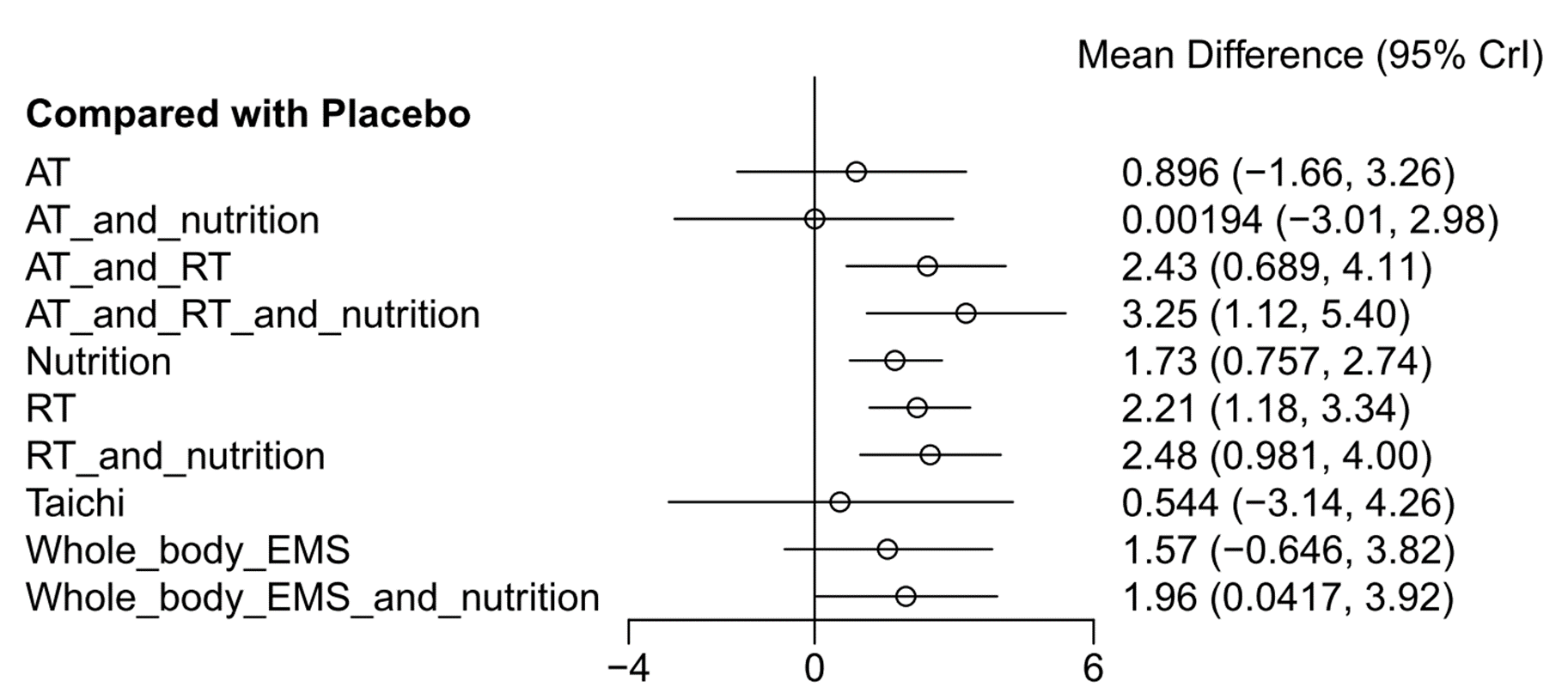


Fig S2k. Forest plot of treatments on chest press


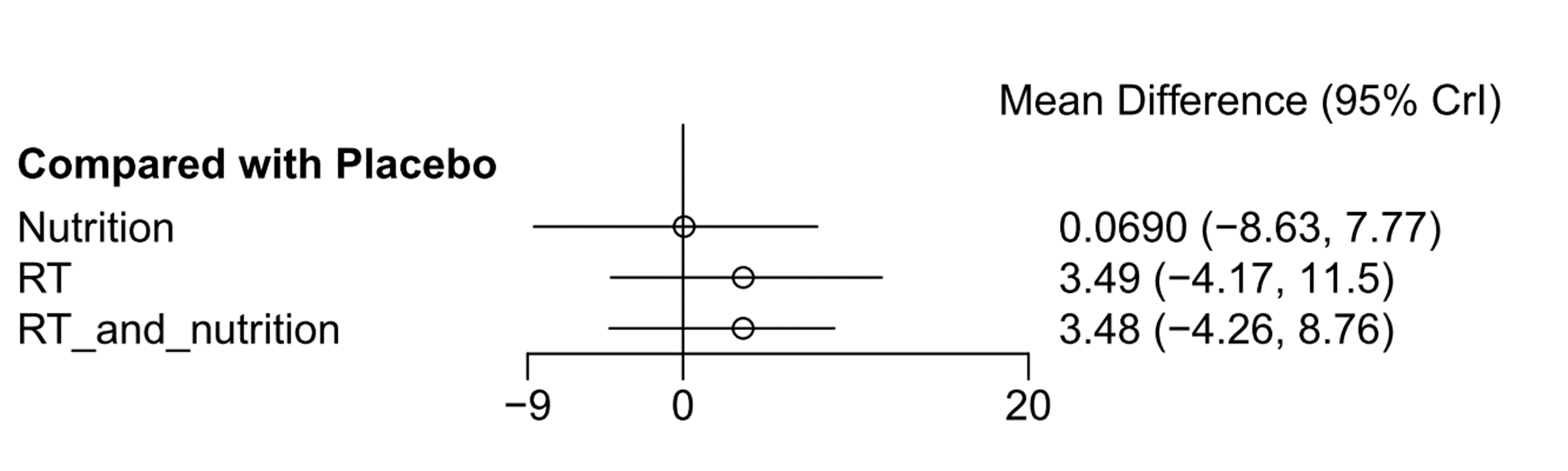


Fig S2l. Forest plot of treatments on leg press


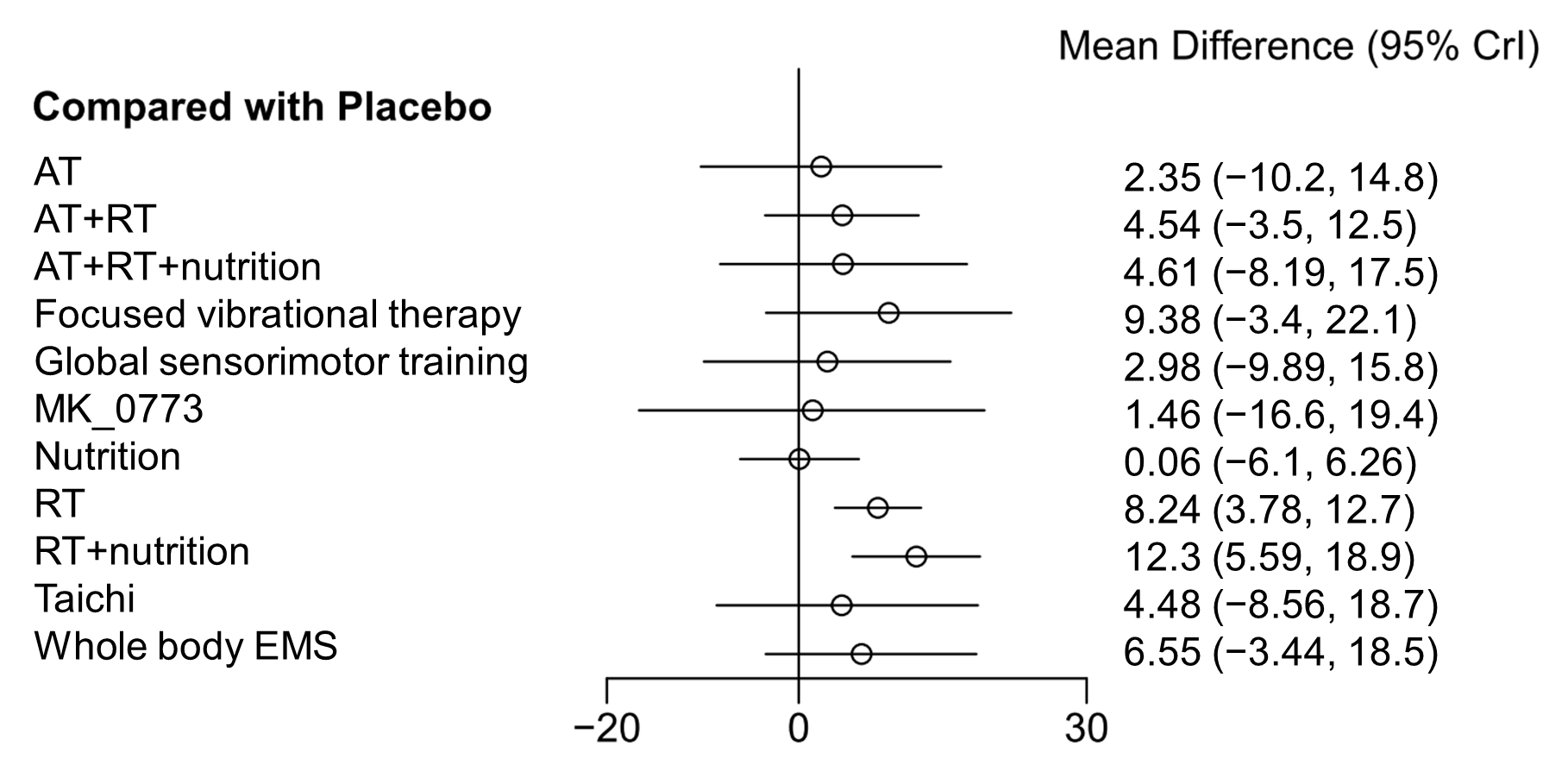


Fig S2m-1. Forest plot of treatments on overall QOL


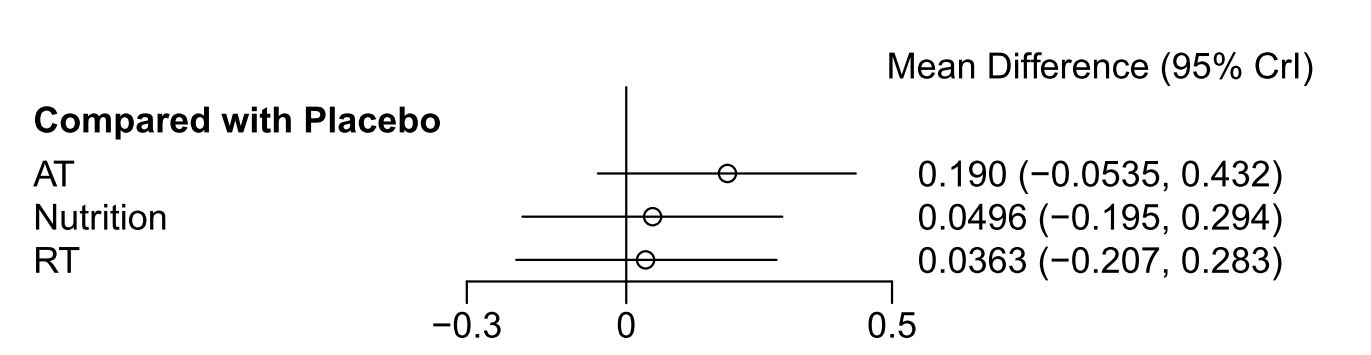


Fig S2m-2. Forest plot of treatments on mental QOL


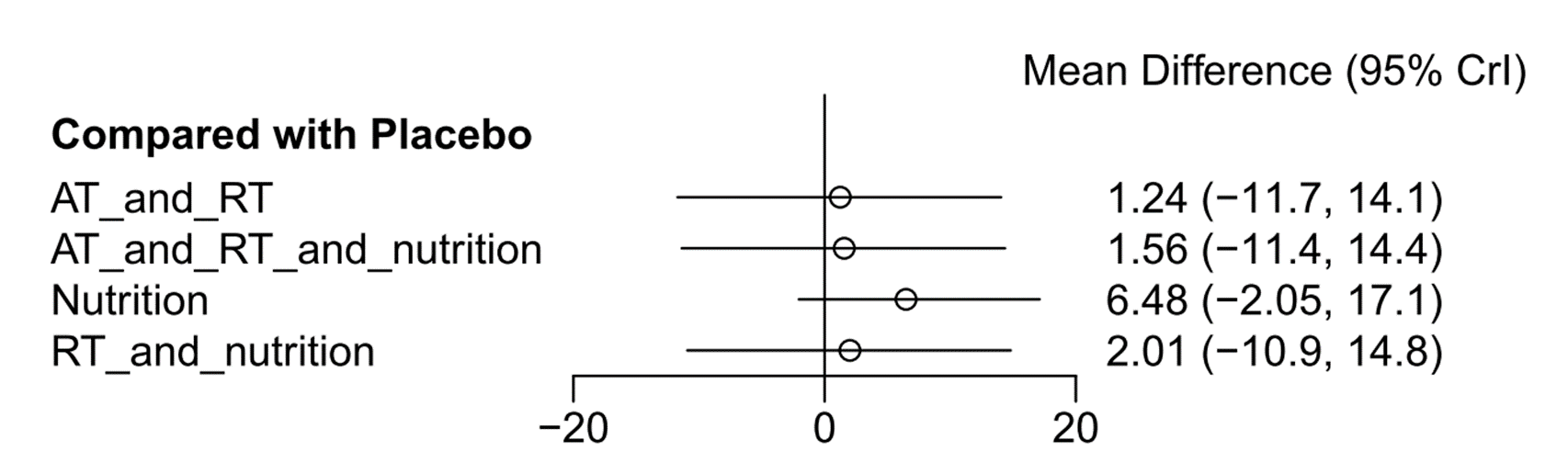


Fig S2m-3. Forest plot of treatments on physical QOL


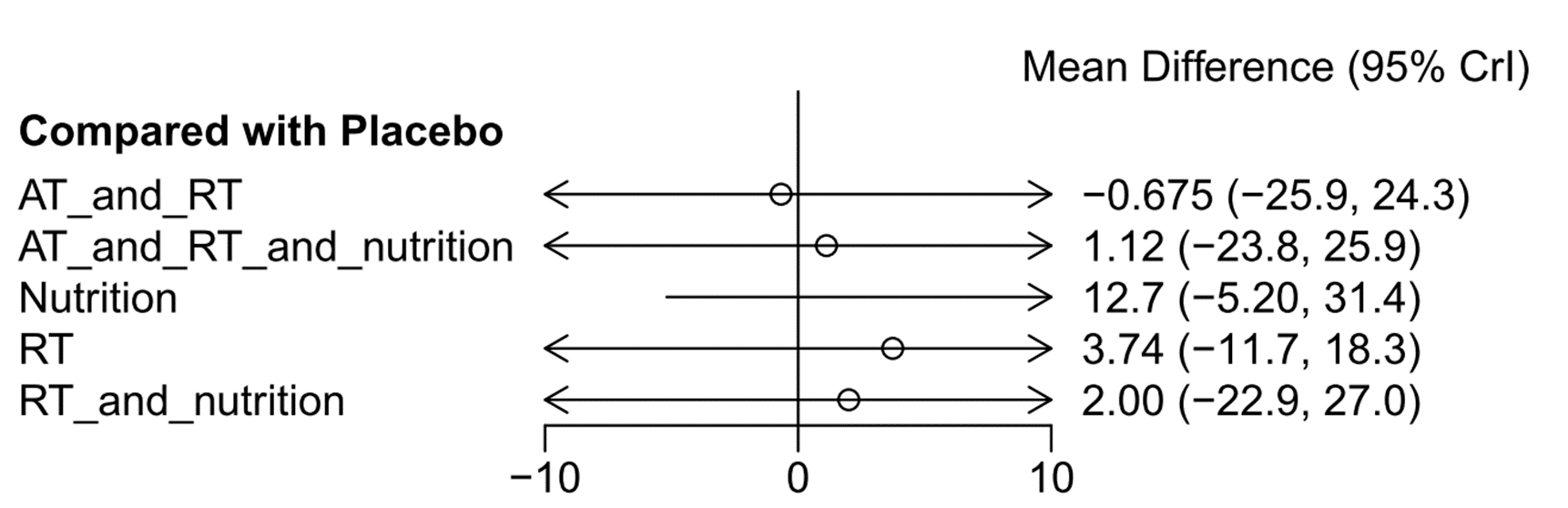


Fig S3a. Ranking regarding treatment effectiveness for 5TSTS assessed by SUCRA values.


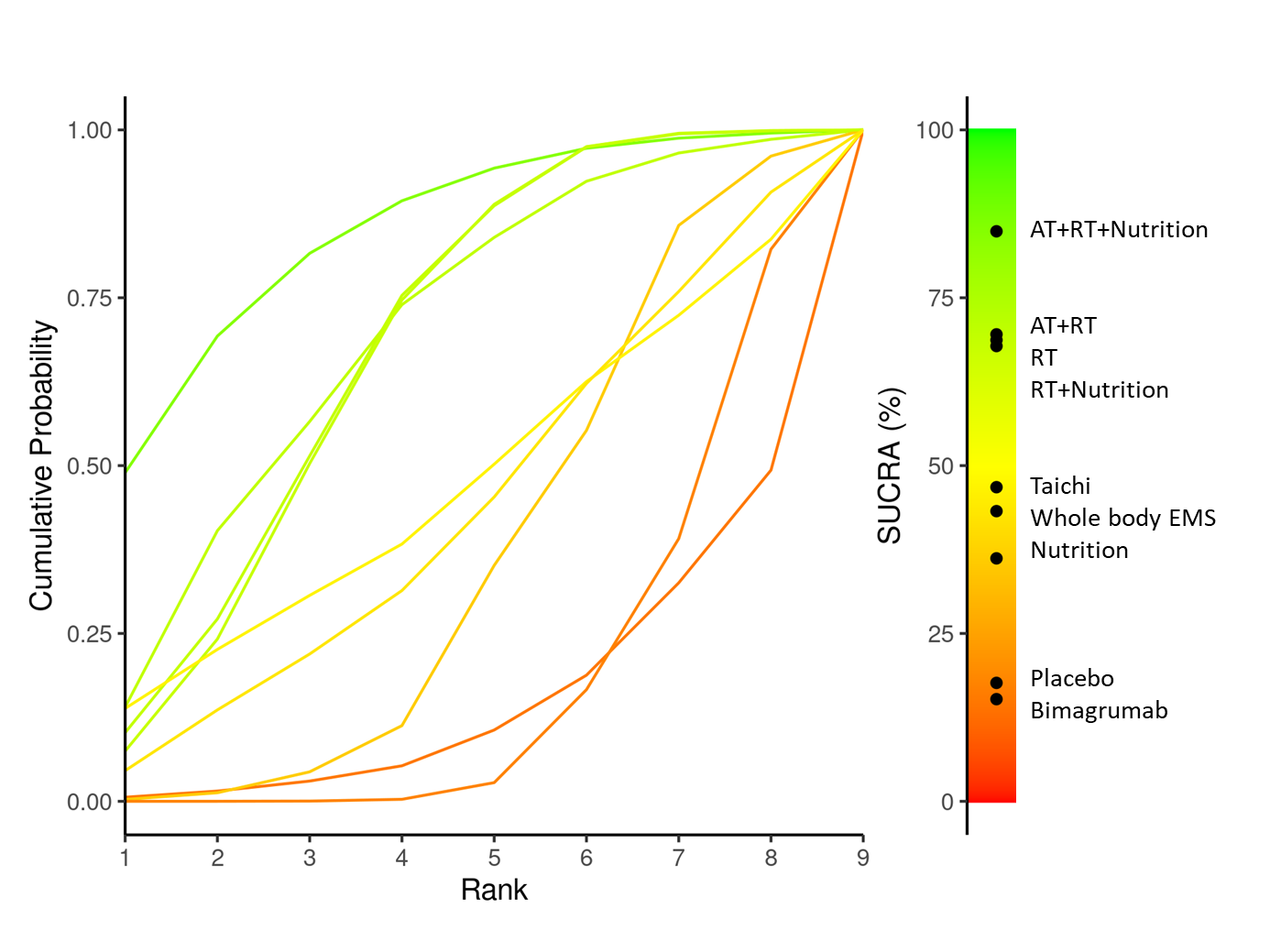


Fig S3b. Ranking regarding treatment effectiveness for 30-second chair stand test assessed by the SUCRA values.


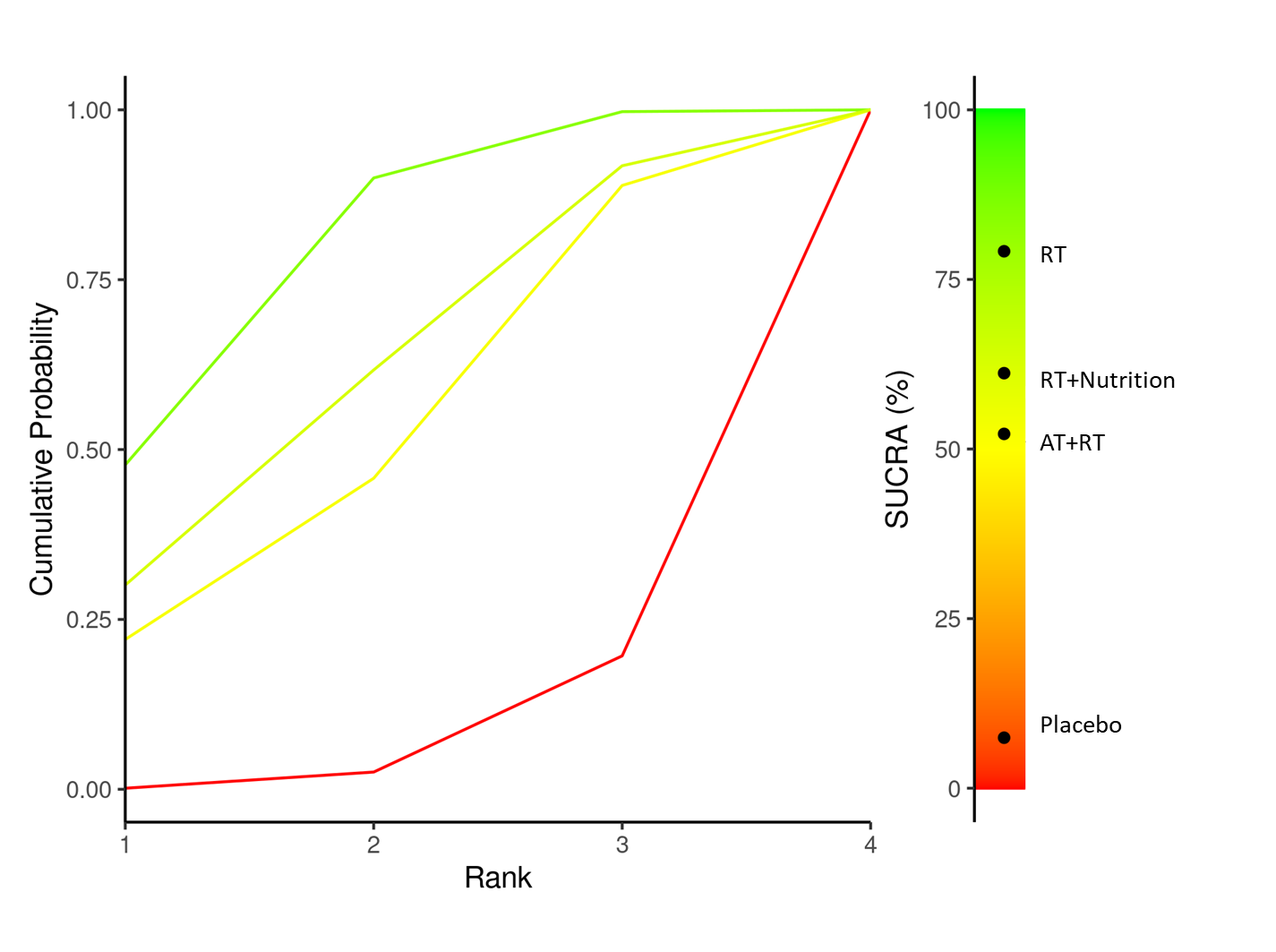


Fig S3c. Ranking regarding treatment effectiveness for TUG assessed by the SUCRA values.


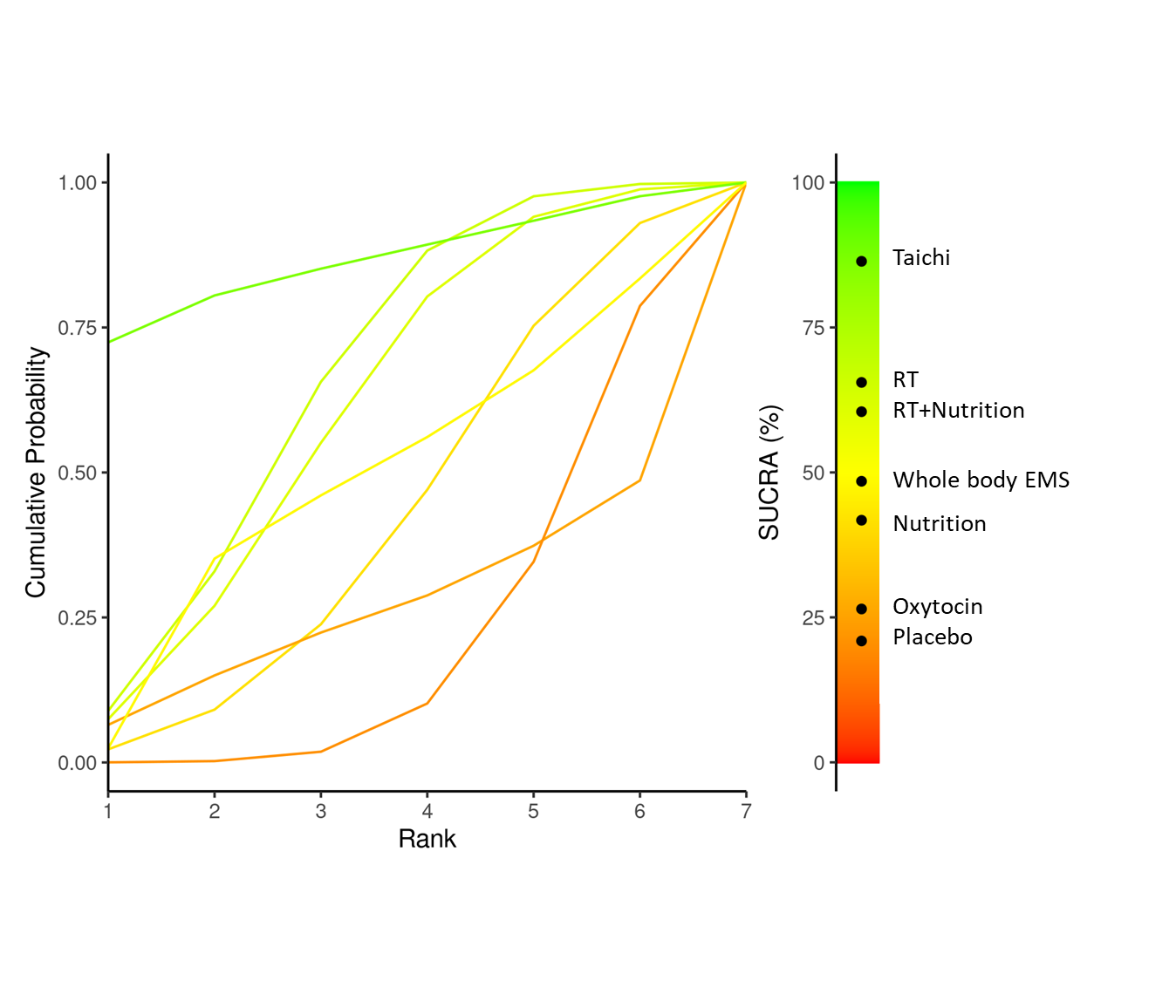


Fig S3d. Ranking regarding treatment effectiveness for SPPB assessed by the SUCRA values.


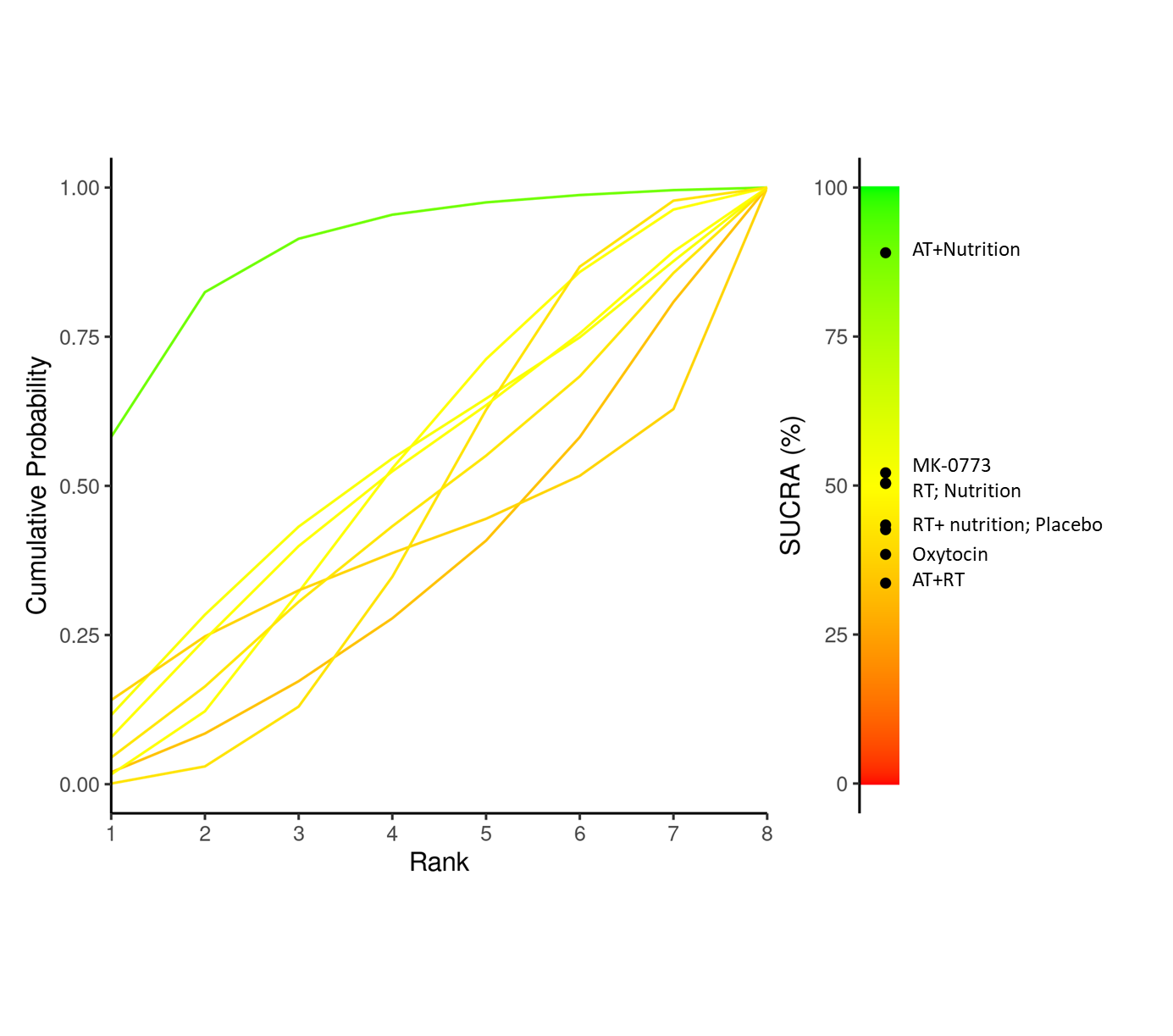


Fig S3e. Ranking regarding treatment effectiveness for gait speed assessed by the SUCRA values.


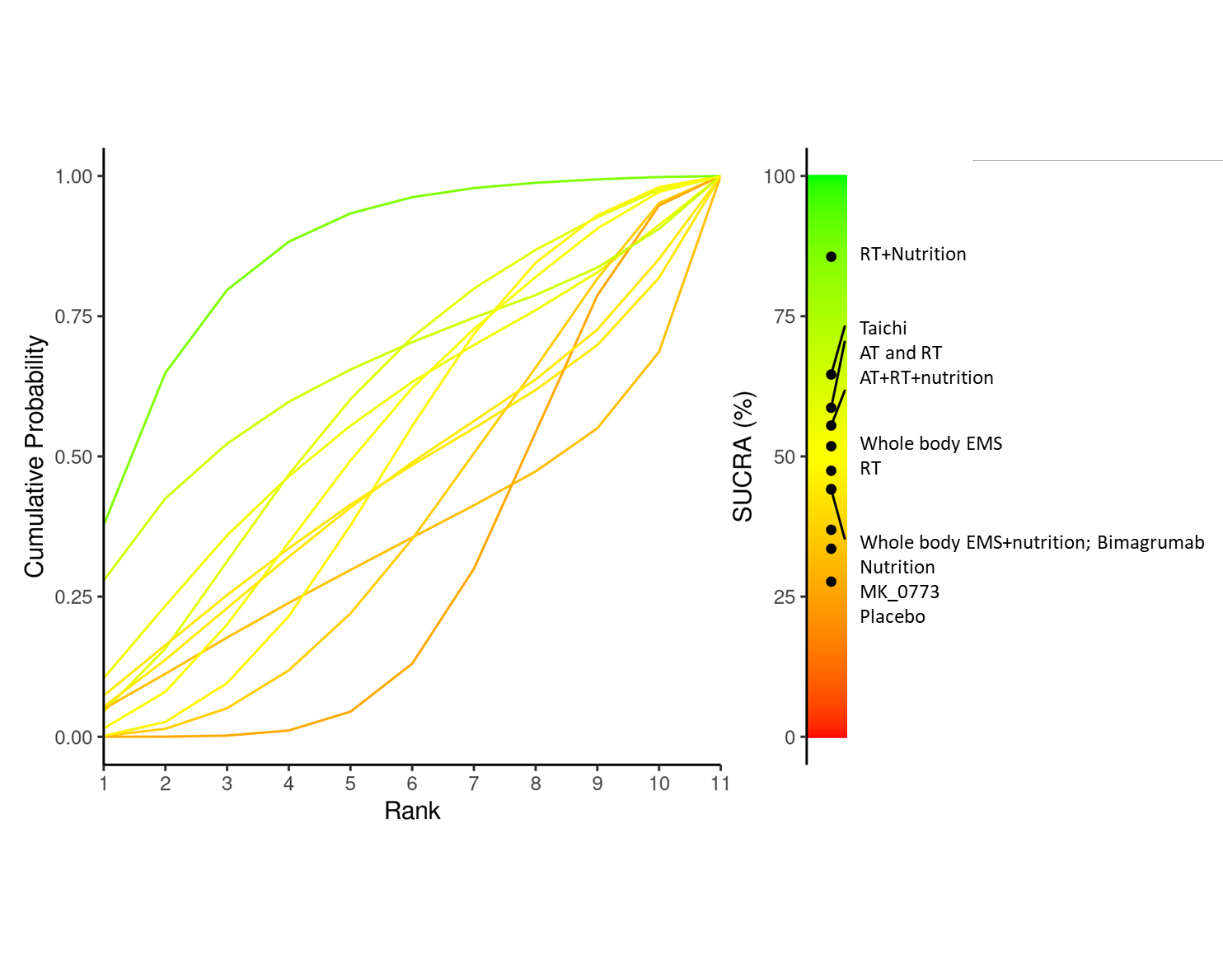


Fig S3f. Ranking regarding treatment effectiveness for 6 min walk test assessed by the SUCRA values.


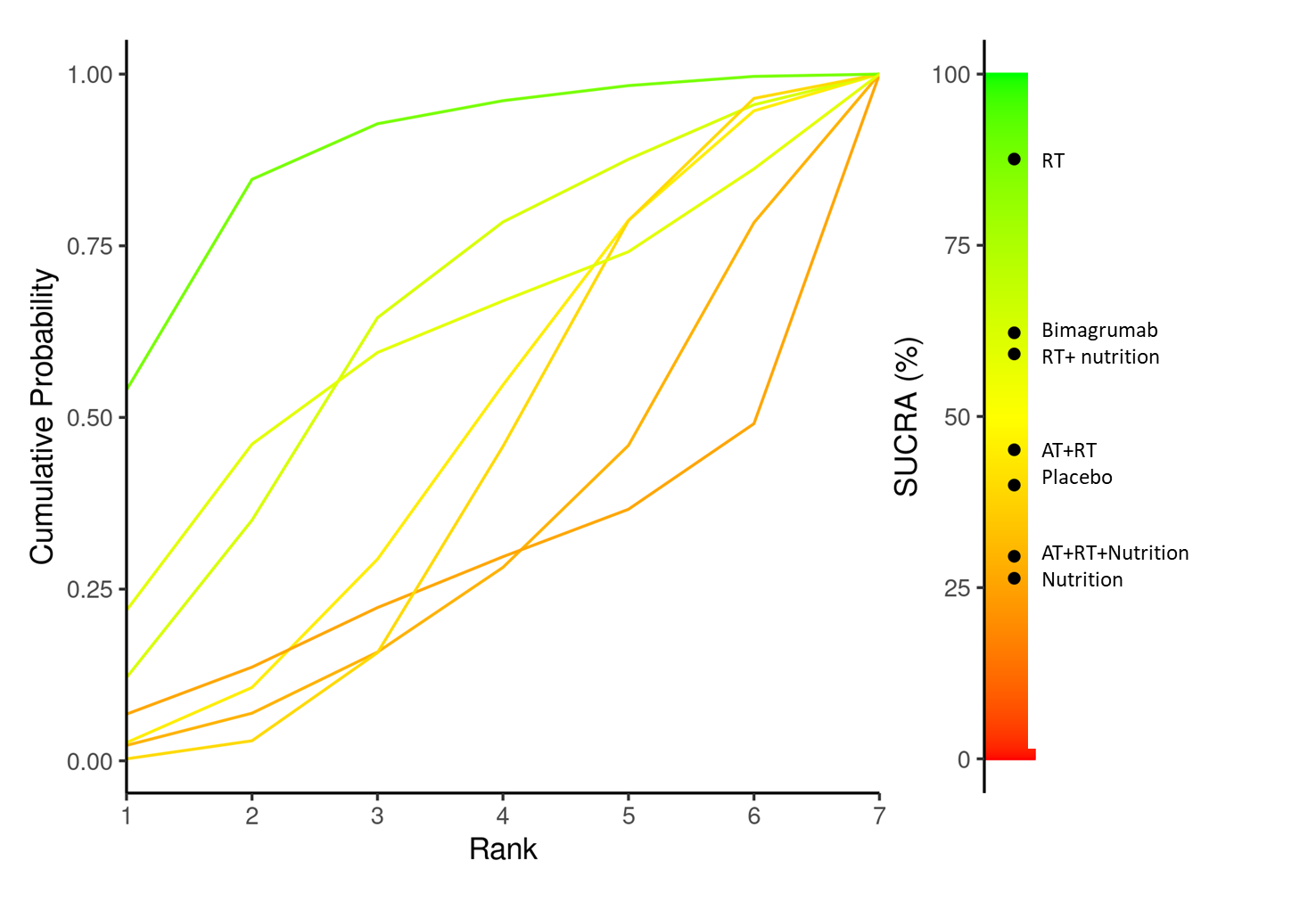


Fig S3g. Ranking regarding treatment effectiveness for ASMI assessed by the SUCRA values.


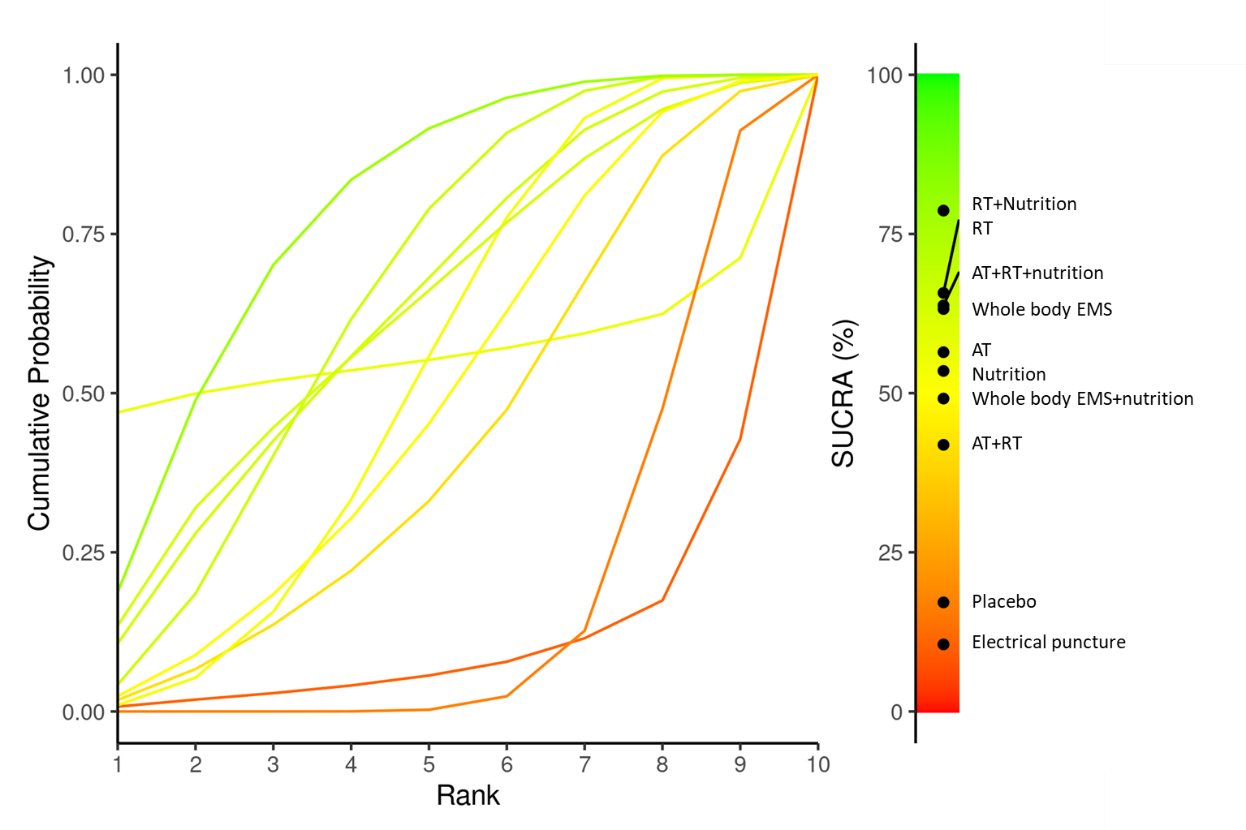


Fig S3h. Ranking regarding treatment effectiveness for leg muscle mass assessed by the SUCRA values.


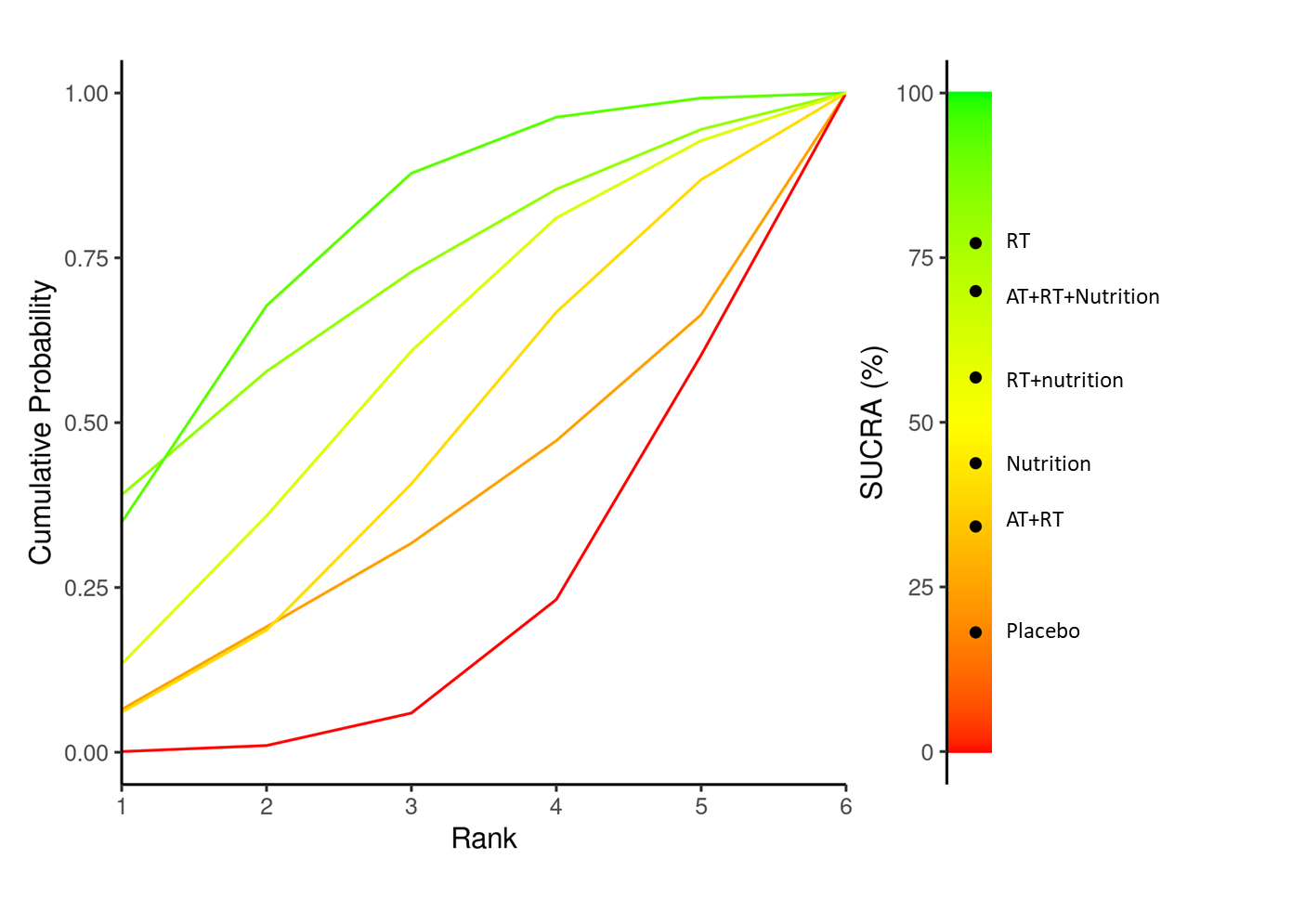


Fig S3i.  Ranking regarding treatment effectiveness for skeletal muscle mass assessed by the SUCRA values.


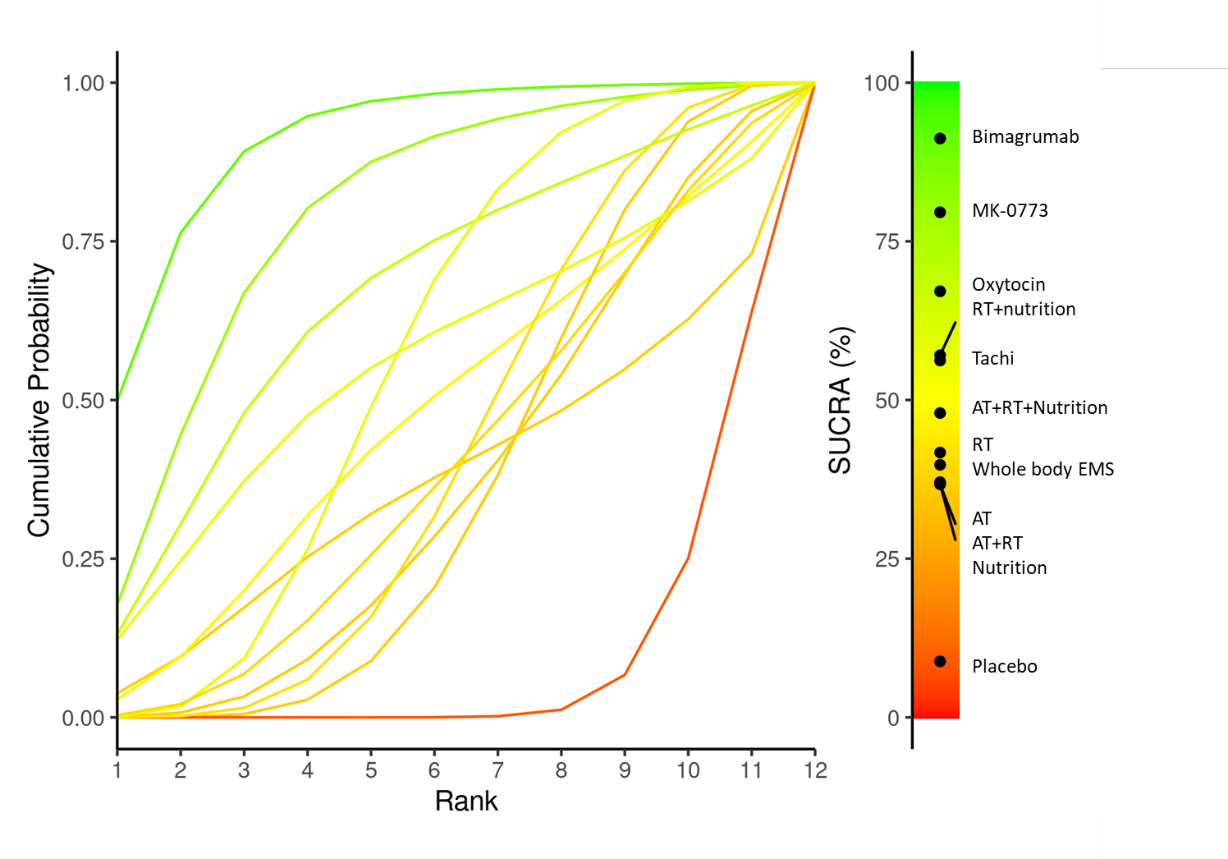


Fig S3j. Ranking regarding treatment effectiveness for handgrip strength assessed by the SUCRA values.


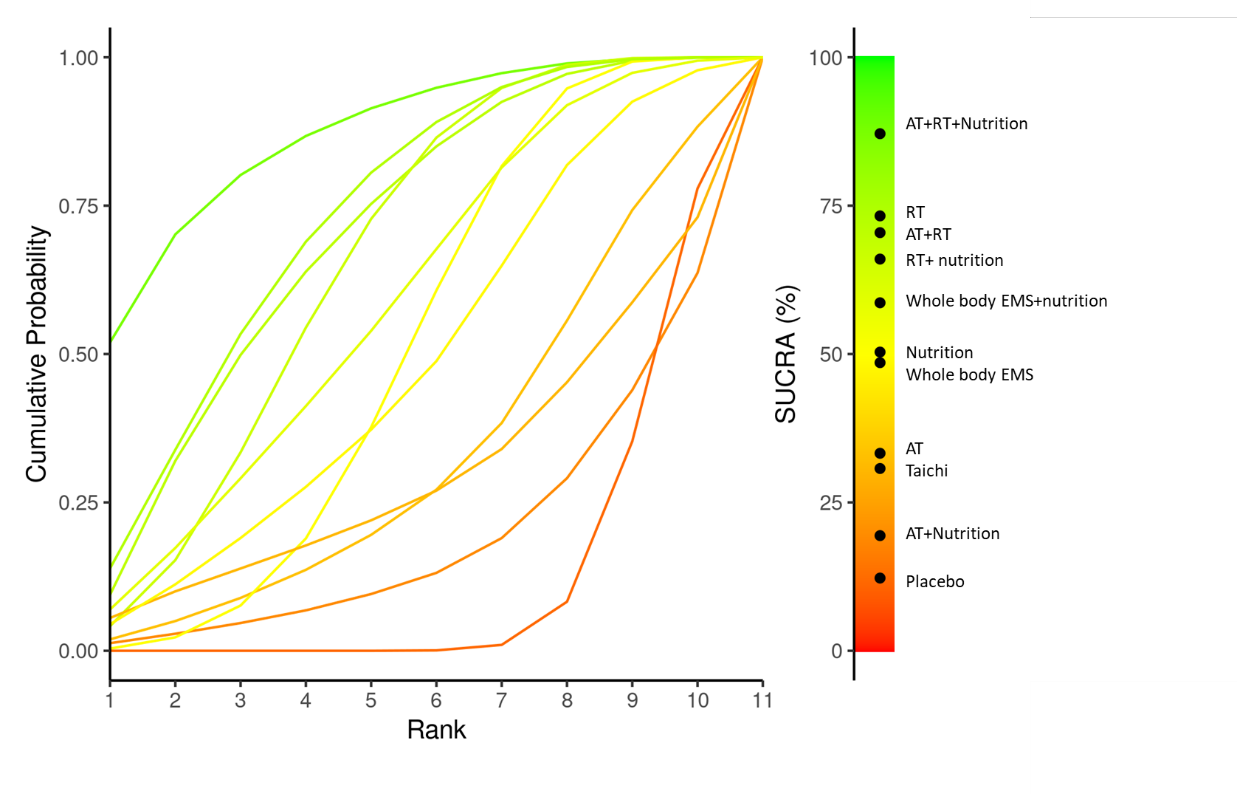


Fig S3k. Ranking regarding treatment effectiveness for chest press assessed by the SUCRA values.


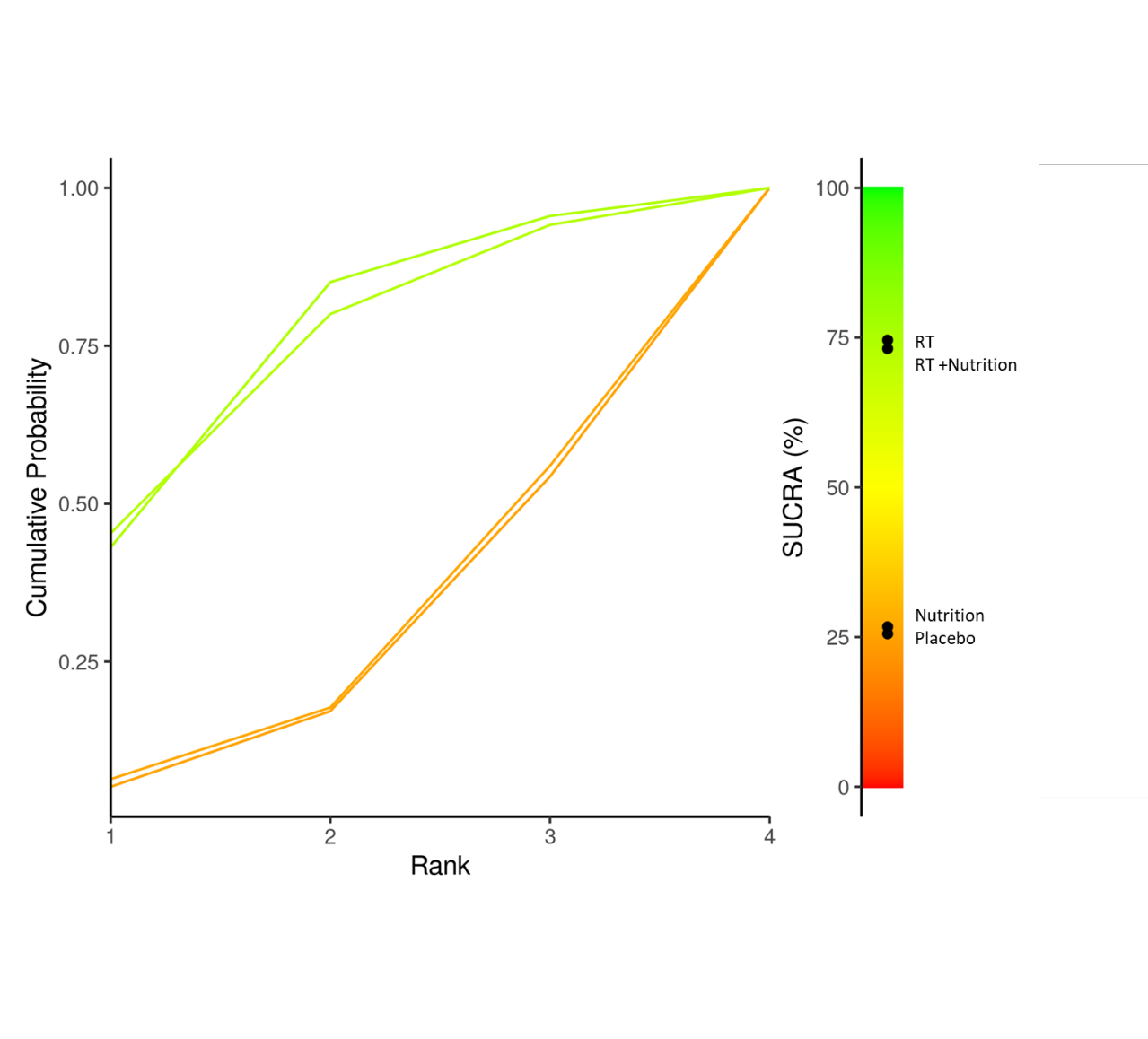


Fig S3l. Ranking regarding treatment effectiveness for leg press assessed by the SUCRA values.


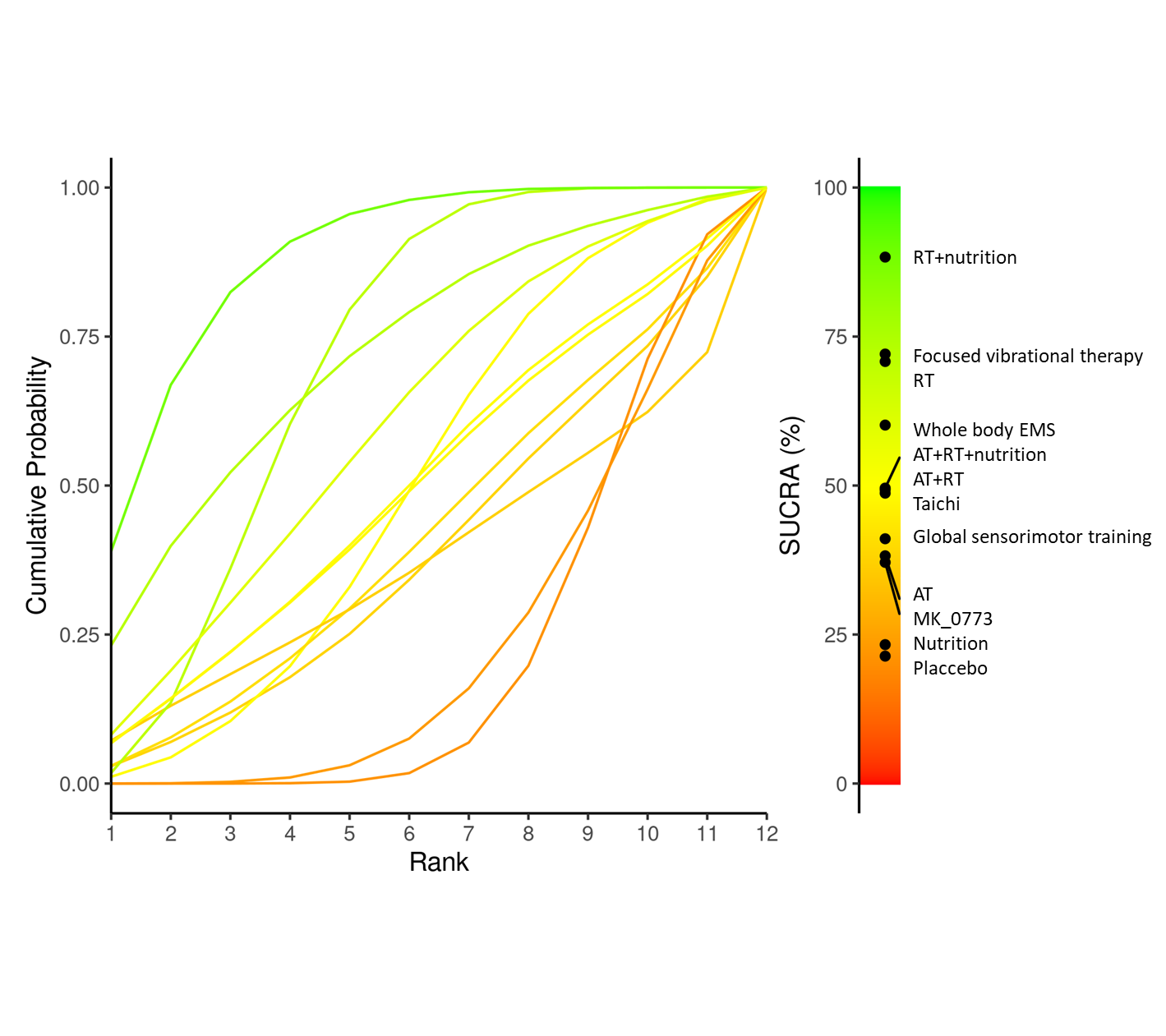


Fig S3m-1. Ranking regarding treatment effectiveness for overall QOL assessed by the SUCRA values.


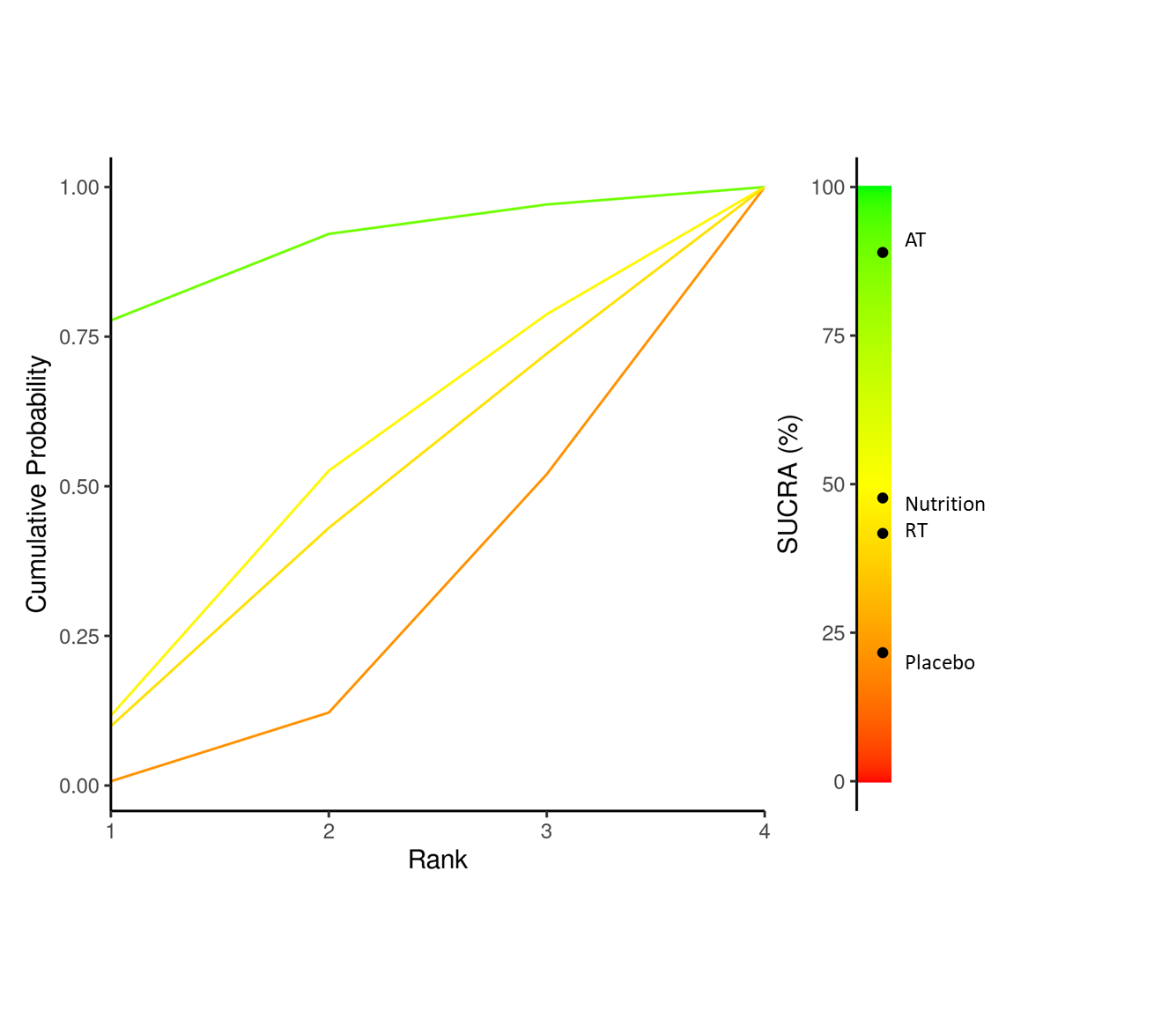


Fig S3m-2. Ranking regarding treatment effectiveness for mental QOL assessed by the SUCRA values.


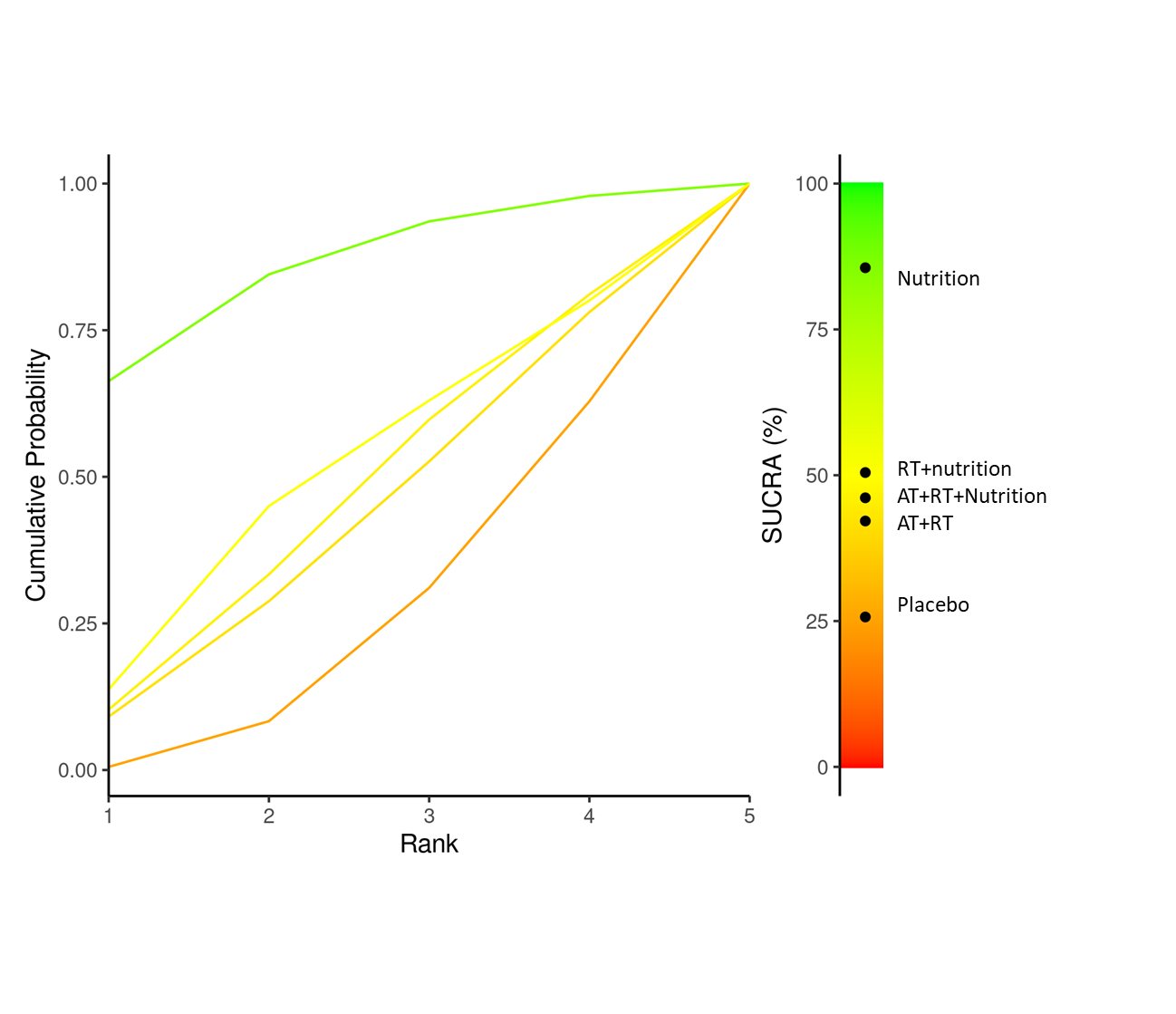


Fig S3m-3. Ranking regarding treatment effectiveness for physical QOL assessed by the SUCRA values.


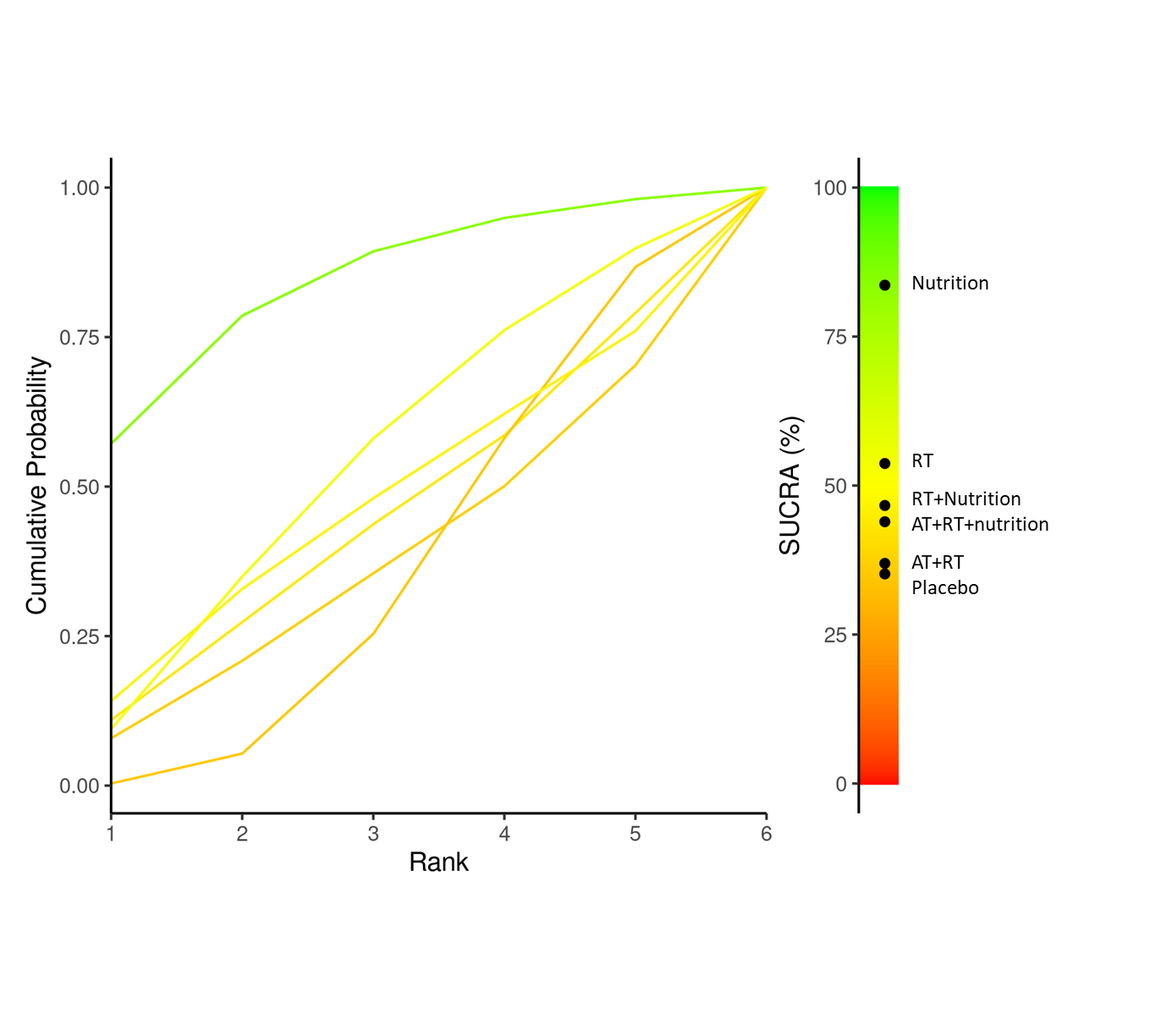


Table S1. Risk of bias assessment using ROB2

| Author | Year | Randomization process | Deviations from intended process | Missing outcome data | Measurement of the outcome | Selection of reported result | Overall bias |
| --- | --- | --- | --- | --- | --- | --- | --- |
| Aleman-Mateo et al. | 2012 | Low risk | Low risk | Some concerns | Low risk | Low risk | Some concerns |
| Papenicolaou D, et al. | 2013 | Low risk | Low risk | Low risk | Low risk | Low risk | Low risk |
| Bellomo et al. | 2013 | Low risk | Low risk | High risk | Low risk | Low risk | High risk |
| Liu C, et al. | 2014 | Some concerns | Low risk | Low risk | Some concerns | Low risk | Some concerns |
| Bauer et al. | 2015 | Low risk | Low risk | Low risk | Low risk | Low risk | Low risk |
| Zdzieblik D et al. | 2015 | Low risk | Low risk | Low risk | Low risk | Low risk | Low risk |
| Cramer J, et al. | 2016 | Low risk | Low risk | Low risk | Low risk | Low risk | Low risk |
| Kim H, et al. | 2016 | Low risk | Some concerns | Low risk | Low risk | Low risk | Some concerns |
| Vasconce los et al. | 2016 | Low risk | Low risk | Low risk | Low risk | Low risk | Low risk |
| Maltias et al. | 2016 | Some concerns | High risk | High risk | High risk | Some concerns | High risk |
| Maltias et al. | 2016 | Low risk | Low risk | Low risk | Some concerns | Low risk | Some concerns |
| Kemmler W, et al. | 2016 | Low risk | Low risk | Low risk | Low risk | Low risk | Some concerns |
| Rondanelli, et al. | 2016 | Low risk | Low risk | Low risk | Low risk | Low risk | Low risk |
| Maruya, et al. | 2016 | Low risk | Low risk | Some concerns | Low risk | Low risk | Some concerns |
| Liao et al. | 2017 | Low risk | Low risk | Low risk | Low risk | Low risk | Low risk |
| Park et al. | 2017 | Low risk | Low risk | Low risk | Low risk | Some concerns | Some concerns |
| Huang S, et al. | 2017 | Low risk | Low risk | Low risk | Low risk | Low risk | Low risk |
| Kemmler W, et al. | 2017 | Low risk | Low risk | Low risk | Low risk | Low risk | Low risk |
| Sammarco et al. | 2017 | Low risk | Low risk | Low risk | Low risk | Low risk | Low risk |
| Wei, et al. | 2017 | Low risk | Low risk | Low risk | Low risk | Low risk | Low risk |
| Wei et al. | 2017 | Low risk | Low risk | Low risk | Low risk | Low risk | Low risk |
| Piastra G, et al. | 2018 | Some concerns | Low risk | Low risk | Low risk | Low risk | Some concerns |
| Liao et al. | 2018 | Some concerns | Low risk | Low risk | Low risk | Some concerns | Some concerns |
| Chen et al. | 2018 | Low risk | Low risk | Low risk | Low risk | Low risk | Low risk |
| Kemmler et al. | 2018 | Low risk | Low risk | Low risk | Low risk | Low risk | Low risk |
| Chen et al. | 2018 | Low risk | Low risk | Low risk | Low risk | Some concerns | Some concerns |
| Von Berens A, et al. | 2018 | Low risk | Low risk | Low risk | Low risk | Low risk | Low risk |
| Tsekoura M, et al. | 2018 | Low risk | Low risk | Low risk | Low risk | Low risk | Low risk |
| Zhou X, et al. | 2018 | Some concerns | Low risk | Low risk | Low risk | Low risk | Some concerns |
| Mafi F, et al. | 2019 | Low risk | Low risk | Low risk | Low risk | Low risk | Low risk |
| Vikberg et al. | 2019 | Low risk | Some concerns | Low risk | Low risk | Low risk | Low risk |
| Zhu et al. | 2019 | Low risk | Low risk | High risk | High risk | Low risk | High risk |
| Amasene et al. | 2019 | Some concerns | Low risk | Low risk | Low risk | Low risk | Some concerns |
| Nabuco et al. | 2019 | Low risk | Low risk | Low risk | Low risk | Low risk | Low risk |
| Zhu, et al. | 2019 | High risk | Low risk | Low risk | Low risk | Some concerns | High risk |
| Bo Y, et al. | 2019 | Low risk | Low risk | Low risk | Low risk | Low risk | Low risk |
| Yamada, et al. | 2019 | Low risk | Low risk | Low risk | Low risk | Low risk | Low risk |
| Oh , et al. | 2020 | Some concerns | Low risk | Low risk | Low risk | Low risk | Some concerns |
| Liao et al. | 2020 | Low risk | Low risk | Low risk | Low risk | Low risk | Low risk |
| Chang et al. | 2020 | Some concerns | Some concerns | Low risk | Low risk | Low risk | Some concerns |
| Bjorkman et al. | 2020 | Low risk | Low risk | Low risk | Low risk | Low risk | Low risk |
| Bagheri R, et al. | 2020 | Some concerns | Low risk | Low risk | Some concerns | Low risk | Some concerns |
| Rooks D, et al. | 2020 | Low risk | Low risk | Low risk | Low risk | Low risk | Low risk |
| Lee et al. | 2021 | Some concerns | Some concerns | Low risk | Low risk | Low risk | Some concerns |
| Osuka et al. | 2021 | Low risk | Low risk | Low risk | Low risk | Low risk | Low risk |
| Seo et al. | 2021 | Low risk | Low risk | Low risk | Low risk | Low risk | Low risk |
| Li Z et al. | 2021 | Low risk | Low risk | Some concerns | Low risk | Low risk | Some concerns |
| Nasimi N, et al. | 2021 | Low risk | Low risk | Low risk | Low risk | Low risk | Low risk |
| Espinoza S, et al. | 2021 | Low risk | Low risk | Some concerns | Low risk | Low risk | Some concerns |
| The LACE study group | 2022 | Low risk | Low risk | Low risk | Low risk | Low risk | Low risk |

Table S2. Details of baseline characteristics of included studies

| Study and Year | Country | Intention to treat or per protocol | Lean mass measurement | Lean mass or fat free mass (kg) | Exercise intensity-Resistance | Gait speed(m/s) |
| --- | --- | --- | --- | --- | --- | --- |
| Alemán-Mateo et al. (2012)[1] | México | Intention to treat | DXA | Baseline: 37.1 (6.3) vs 36.8 (6.4)  Follow-up: 37.9 (6.5) vs 37.6 (6.4) | No data | No data |
| Bellomo et al.(2013)[2] | Italy | Per protocol | No data | No data | Vigorous | No data |
| Papanicolaou et al. (2013)[3] | Asain, Multi-Racial, White | No data | DXA | Baseline: 30.94 (3.39) vs 30.83 (3.62)  Change: 1.26 (1.09) vs 0.29 (1.29) | No data | Baseline 69.15 (14.30) vs 68.21 (13.60)  Change 6.24 (17.65) vs 8.91 (15.05) |
| Liu et al. (2014)[4] | US | No data | DXA | No data | Moderate | PA: baseline: 0.90 (95%CI:0.84-0.96), 6 months:0.92 (95%CI:0.84-1.00), 12 months:0.93 (95%CI:0.85-1.01)  SA: baseline: 0.90 (95%CI:0.84-0.96), 6 months:0.97 (95%CI:0.89-1.04), 12 months:0.89 (95%CI:0.81-0.96) |
| Bauer et al. (2015)[5] | Belgium, Germany, Ireland, Italy, Sweden, and the United Kingdom | Intention to treat | BIA (screening), DXA (outcome) | No data | No data | Active: baseline: 0.8 (0.2), week 7 changes: 0.03 (0.11), week 13 changes: 0.07 (0.12)  Control: baseline: 0.8 (0.2), week 7 changes: 0.03 (0.10), week 13 changes: 0.05 (0.12) |
| Zdzieblik et al. (2015)[6] | Germany | Per protocol | DXA | Fat free mass(kg): treatment group baseline 56.9(SD6.68), after-intervention 61.1(SD6.88)  placebo group baseline 54.9(SD6.96) after-intervention 57.8(SD7.46) | Vigorous | No data |
| Cramer et al. (2016)[7] | 8 countries across Europe and North America | No data | DXA | (1) Leg muscle mass (LMM)  < SARCOPENIA >  Control: median baseline 13 (11, 16), change after 12 wks: 0.05 (-0.36, 0.56), change after 24 wks: -0.08 (-0.39, 0.40)  Experimental: median baseline 13 (10, 16), change after 12 wks: -0.05 (-0.28, 0.29), change after 24 wks: 0.06 (-0.27, 0.39)  < SEVERE SARCOPENIA >  Control: median baseline 11 (9, 13), change after 12 wks: 0.14 (-0.10, 0.41), change after 24 wks: 0.10 (-0.32, 0.57)  Experimental: median baseline 11 (10, 14), change after 12 wks: 0.08 (-0.26, 0.54), change after 24 wks 0.10 (-0.31, 0.46)  (2) Tested leg muscle mass (TLMM)  Control: median baseline 6.4 (5.3, 8.1), change after 12 wks: 0.10 (0.16, 0.24), change after 24 wks: 0.05 (0.25, 0.21)  Experimental: median baseline 6.3 (5.3, 8.0), change after 12 wks: 0.01 (0.20, 0.25), change after 24 wks: 0.04 (0.13, 0.21)  < SEVERE SARCOPENIA >  Control: median baseline 5.4 (4.8, 6.6), change after 12 wks: 0.07 (0.14, 0.23), change after 24 wks: 0.05 (0.18, 0.40)  Experimental: median baseline 5.7 (5.1, 6.9), change after 12 wks: 0.00 (0.24, 0.25), change after 24 wks 0.02 (0.21, 0.20) | No data | Control group: baseline median 0.84 (0.79, 0.95), median improvement 0.02 (-0.04, 0.10) at 12 wks and 0.02 (-0.03, 0.11) at 24 wks  Experimental: baseline median 0.87 (0.79, 0.97), median improvement 0.01 (-0.05, 0.08) at 12 wks and 0.05 (-0.04, 0.15) at 24 wks |
| Kemmler et al. (2016)[8] | Germany | No data | No data | No data | No data | Baseline 1.14 (1.08 to 1.20) 1.17 (1.10 to 1.25) 1.23 (1.13 to 1.32)  Changes .08 (.01 to .15) .03 (−.04 to .10) −.03 (−.10 to .04) |
| Kim et al. (2016)[9] | Japan | No data | DXA | E+N: baseline12.8 ± 2.2, follow up 13.0 ± 2.3  E: baseline 12.7 ± 2.2, follow up 13.0 ± 2.2  N: baseline 13.2 ± 2.4, follow up 13.4 ± 2.4  HE: baseline 12.6 ± 1.5, follow up 12.9 ± 1.6 | Mild | E+N: baseline 1.1 ± 0.2, follow up 1.2 ± 0.2  E: baseline 1.1 ± 0.2, follow up 1.3 ± 0.2  N: baseline 1.2 ± 0.2, follow up 1.2 ± 0.2  HE: baseline 1.1 ± 0.2, follow up 1.2 ± 0.2 |
| Maltais et al. (2016)[10] | Canada | No data | DXA | Nondairy: T1: 56.3 ± 8.1, T2: 58.2 ± 7.9  Dairy: T1: 54.5 ± 5.3, T2: 56.2 ± 4.5  Control: T1: 56.2 ± 6.3, T2: 57.6 ± 6.8 | Vigorous | No data |
| Maltais et al. (2016)[11] | Canada | No data | DXA | Control: Baseline:56.2±6.3, post:57.6 ± 6.8;  EAA supp: Baseline:56.3± 8.1 , post:58.2 ± 7.9;  EAA milk: Baseline:54.5± 5.3, post: 56.2 ± 4.5 | No data | Normal gait speed:  Control: baseline: 1.5 ± 0.5, post:1.4 ± 0.3;  EAA supp: baseline: 1.2 ± 0.3, post: 1.5 ±0.3  EAA milk: baseline: 1.4 ± 0.2, post: 1.5 ±0.2 |
| Maruya et al.(2016)[12] | Japan | Per protocol | No data | No data | Mild | Baseline: intervention group 1.48 ± 0.27, control group 1.48 ± 0.26; post-intervention: intervention group 1.46 ± 0.20, control group 1.42 ± 0.17 |
| Rondanelli et al.(2016)[13] | Italy | No data | DXA | Change from baseline: Dietary supplement group: 1.382 (0.273), Placebo group: -0.484 (0.288) | Moderate | No data |
| Vasconcelos et al. (2016)[14] | Brazil | Intention to treat | No data | No data | Moderate- Vigorous | EG: baseline: 1.09±0.10, week 10: 1.11±0.16  CG: baseline: 1.04±0.19, week 10: 1.09±0.11 |
| Chen et al. (2017)[15] | Taiwan | Per protocol | BIA | CON: baseline: 21.6 ± 3.6, 8 wks: 21.2 ± 3.8, 12 wks: 20.9 ± 3.5  RT: baseline: 22.9 ± 4.0, 8 wks: 23.0 ± 4.1, 12 wks: 23.1 ± 4.0  AT: baseline: 20.0 ± 3.3, 8 wks: 20.2 ± 3.2, 12 wks: 20.2 ± 2.3  CT: baseline: 20.7 ± 4.0, 8 wks: 21.4 ± 3.7, 12 wks: 20.9 ± 3.7 | Moderate | No data |
| Huang et al. (2017)[16] | Taiwan | Intention to treat | DXA | ERT group: pre 34.29 ± 4.02, post 32.81 ± 8.45  Control group: pre 36.06 ± 4.02, post 35.45 ± 4.02 | Moderate | No data |
| Kemmler et al. (2017)[17] | No data | Intention to treat | No data | No data | No data | No data |
| Liao et al. (2017)[18] | Taiwan | Intention to treat | DXA | < Fat free mass, kg >  EG: baseline: 36.58±4.30, 12 wks: 36.86±4.35  CG: baseline: 37.00±4.08, 12 wks: 36.56±3.68  < Leg lean mass, kg >  EG: baseline: 10.42±1.43, 12 wks: 11.08±1.66  CG: baseline: 11.08±1.66, 12 wks: 10.57±1.68 | Moderate | EG: baseline: 1.51±0.28, 12 wks: 1.53±0.23  CG: baseline: 1.16±0.28, 12 wks: 1.14±0.20 |
| Park et al. (2017)[19] | South Korea | Intention to treat | BIA | Exercise: baseline: 14.1±1.8, 24 weeks: 14.5±1.9  Control: baseline: 15.0±2.0, 24 weeks: 14.9±1.6 | Moderate- Vigorous | Exercise: baseline: 1.49±0.21, 24 weeks: 1.64±0.21  Control: baseline: 1.47±0.21, 24 weeks: 1.43±0.21 |
| Sammarco et al.(2017)[20] | Italy | Intention to treat | BIA | Low-calorie diet(control): baseline 47.7 ± 3.34, post intervention48.0 ± 2.83; low-calorie high-protein diet: baseline 47.6 ± 2.45, post intervention 48.7 ± 2.11 | No data | No data |
| Wei et al.(2017)[21] | China | Intention to treat | No data | No data | No data | No data |
| Wei et al.(2017)[22] | China | Intention to treat | No data | No data | No data | Low-frequency group: pre-intervention:0.94(0.13), post-intervention: 1.01(0.15); medium- frequency group: pre-intervention:0.96(0.19); post-intervention: 1.05(0.16); high-frequency group:pre-intervention:1.08(0.22); post-intervention: 1.07(0.15); control group: pre-intervention:1.03(0.17); post-intervention: 1.02(0.17) |
| Chen et al. (2018)[23] | Taiwan | Unclear | BIA | < Skeletal muscle mass, kg >  KT: baseline: 20.99 ± 3, week 8: 21.16 ± 2.87, week 12: 21.26 ± 3.06  CON: baseline: 19.99 ± 2.74, week 8: 19.7 ± 2.74, week 12: 19.48 ± 2.39  < Appendicular skeletal muscle mass, kg >  KT: baseline: 15.46 ± 2.44, week 8: 15.72 ± 2.39, week 12:15.71 ± 2.35  CON: baseline: 14.71 ± 2.21, week 8: 14.57 ± 2.3, week 12: 14.55 ± 2.28 | Moderate- Vigorous | No data |
| Kemmler et al. (2018)[24] | Germany | Intention to treat | BIA and MRI | < Intra-fascial total volume of the mid-thigh (cm3) >  EMS&P: baseline: 357.6±44.9, changes: 8.65±10.55  CG: baseline: 359.8±44.3, changes: 7.05±8.89  < Intra-fascial fat-free muscle volume of the mid-thigh (cm3) >  EMS&P: baseline: 297.8±38.2, changes: 8.47±10.37  CG: baseline: 292.1±37.5, changes: 1.77±10.9  < Appendicular skeletal muscle mass (kg) >  EMS&P: baseline: 18.5±1.7, changes: 0.45±0.53  CG: baseline: 18.4±1.6, changes: −0.01±0.47 | Moderate- Vigorous | EMS&P: baseline: 1.26±0.20, changes: 0.035±0.046  CG: baseline: 1.27±0.18, changes: −0.006±.039 |
| Liao et al. (2018)[25] | Taiwan | Intention to treat | DXA | < Total skeletal mass, kg >  EG: T0: 34.78(4.14), T1: 35.06(4.22), T2: 35.00(4.24)  CG: T0: 35.19(4.00), T1: 34.75(3.56), T2: 34.70(3.71)  < Appendicular lean muscle mass, kg >  EG: T0: 14.21(2.03), T1: 14.39(2.11), T2: 14.26(2.20)  CG: T0: 13.97(1.87), T1: 13.78(1.65), T2: 13.56(1.56) | Moderate | EG: T0: 1.44(0.28), T1: 1.46(0.24), T2: 1.48(0.25)  CG: T0: 1.17(0.27), T1:1.16(0.20) , T2: 1.16(0.22) |
| Piastra et al. (2018)[26] | Italy | Per protocol | BIA | < Lean mass >  RESISTANCE: baseline 19.50±6.59, f/u T1=21.25±6.05  POSTURAL: baseline 19.85±7.39, f/u 19.63±6.49  < Skeletal muscle mass >  RESISTANCE: baseline 17.31±1.16, f/u 19.02±6.58  POSTURAL: baseline T0=17.59±7.31, f/u 17.53±6.39 | Mild- Vigorous | No data |
| Tsekoura et al. (2018)[27] | Greece | No data | BIA | Group-based exercise: baseline: 25.59 ± 4.4, after 3-months intervention:26.5 ± 4.4, after 6 months follow-up:25.95 ± 4.68;  Home-based exercise: baseline: 24.41 ± 4.9, after 3-months intervention: 24.73 ± 4.98, after 6 months follow-up:24.38 ± 3.1;  Control: baseline: 25.15 ± 3.52, after 3-months intervention: 24.57 ± 4.66, after 6 months follow-up: 26.45 ± 10.46 | Moderate | Group-based exercise: baseline: 0.87 ± 0.13, after 3-months intervention:1.21 ± 0.15, after 6 months follow-up:1.32 ± 0.3;  Home-based exercise: baseline: 0.9 ± 0.10, after 3-months intervention: 1.08 ± 0.15, after 6 months follow-up:1.11 ± 0.2;  Control: baseline:0.91 ± 0.12, after 3-months intervention: 0.92 ± 0.15, after 6 months follow-up: 0.87 ± 0.15 |
| Von Berens et al. (2018)[28] | US and Sweden | Both | No data | No data | No data | No data |
| Zhou et al. (2018)[29] | China | No data | BIA | No data | No data | No data |
| Amasene et al. (2019)[30] | Spain | Per protocol | DXA | Arm lean mass  Placebo: pre: 2.3 ± 0.67, post: 2.3 ± 0.41  Protein: pre: 2.3 ± 0.44, post: 2.2 ± 0.41  Legs lean mass  Placebo: pre: 6.8 ± 1.7, post: 6.9 ± 1.45  Protein: pre: 6.4 ± 1.08, post: 6.5 ± 1.04  Total lean mass  Placebo: pre: 45.2 ± 9.85, post: 44.7 ± 8.54  Protein: pre: 42.3 ± 6.63, post: 42.5 ± 6.61 | Moderate- Vigorous | No data |
| Bo et al. (2019)[31] | China | No data | BIA | Intervention: baseline: 14.74 ± 2.79, change: 0.23 ± 1.07  Placebo: baseline: 15.21 ± 3.61, post: -0.25 ±0.71 | No data | No data |
| Mafi et al. (2019)[32] | Iran | No data | DXA | No data | Moderate- Vigorous | No data |
| Nabuco et al. (2019)[33] | Brazil | Intention to treat | DXA | < Total lean soft tissue mass >  Whey: pre: 31.8 ± 2.8, post: 33.0 ± 2.9  Placebo: pre: 32.0 ± 2.9, post: 33.5 ± 2.8  < Lower limbs lean soft tissue mass >  Whey: pre: 10.7 ± 0.9, post: 11.2 ± 0.9  Placebo: pre: 10.9 ± 0.9, post: 11.0 ± 0.8  < Appendicular lean soft tissue mass >  Whey: pre: 13.9 ± 0.9, post: 14.7 ± 1.1  Placebo: pre: 13.9 ± 0.8, post: 14.2 ± 0.8 | Moderate | Whey: pre: 7.4 ± 0.8, post: 6.9 ± 0.8  Placebo: pre: 7.4 ± 0.8, post: 6.8 ± 0.6 |
| Vikberg et al. (2019)[34] | Sweden | Per protocol | DXA | Total lean mass  CONTROL: baseline: 41.8±8.64, 10 weeks: 41.9±8.63  INTERVENTION: baseline: 40.8±7.60, 10 weeks: 41.9±7.94  Arm lean mass  CONTROL: baseline: 4.56 ± 1.44, 10 wks: 4.57 ± 1.46  INTERVENTION: baseline 4.30 ± 1.22, 10 wks: 4.53 ± 1.31  Leg lean mass  CONTROL: baseline: 13.6 ± 3.07, 10 wks: 13.6 ± 3.02  INTERVENTION: baseline: 13.2 ± 2.81, 10 wks: 13.6 ± 2.82 | Moderate- Vigorous | No data |
| Yamada et al.(2019)[35] | Japan | Both | BIA | Appendicular muscle mass (kg): Ex+nutrition: change from baseline: 0±1.29, Ex: 0.07±1.11, Nutrition: -0.11±0.92, Control: −0.72 ±1.51 | Mild | No data |
| Zhu et al. (2019a)[36] | China | Intention to treat | DXA | Upper limb muscle mass  Control: baseline 3.06 ± 0.71, 12 wks: 3.06 ± 0.74, 24 wks: 3.19 ± 0.75  Exercise: baseline: 3.11 ± 0.75, 12 wks: 3.24 ± 0.82, 24 wks: 3.22 ± 0.78  Combined: baseline 2.98 ± 0.65, 12 wks: 3.07 ± 0.75, 24 wks: 2.94 ± 0.52  Lower limb muscle mass  Control: baseline: 9.50 ± 2.27, 12 wks: 9.09 ± 2.10, 24 wks: 9.60 ± 2.22  Exercise: baseline 9.63 ± 2.03, 12 wks: 9.64 ± 1.91, 24 wks: 9.59 ± 1.87  Combined: baseline 9.25 ± 1.80, 12 wks: 9.44 ± 1.99, 24 wks: 9.24 ± 1.60 | Mild | Control: baseline: 0.94 ± 0.26, 12 wks: 1.05 ± 0.24, 24 wks: 1.04 ± 0.28  Exercise: baseline: 0.82 ± 0.25, 12 wks: 0.98 ± 0.35, 24 wks: 0.98 ± 0.30  Combined: baseline 0.89 ± 0.26, 12 wks: 1.08 ± 0.23, 24 wks: 1.12 ± 0.22 |
| Zhu et al. (2019b)[37] | China | No data | DXA | Upper limb: TC group: baseline:4.28 ± 0.59, 8 wk: 4.30 ± 0.54; WBV: baseline: 4.35 ± 0.62, 8 wk: 4.43 ± 0.51; Con: baseline: 4.46 ± 0.52, 8wks: 4.41 ± 0.68  Lower limb: TC group: baseline: 13.52 ± 2.28, 8wk:13.60 ± 2.23; WBV: baseline: 13.18 ± 2.77, 8 wk: 13.35 ± 2.21; Con: baseline:13.49 ± 2.03, 8wks: 13.51 ± 1.92  Trunk muscle mass: TC group: baseline: 20.66 ± 1.54, 8 wk: 21.03 ± 1.57; WBV: baseline: 21.74 ± 1.66, 8 wk: 21.79 ± 1.75, Con: baseline: 21.98 ± 1.63, 8wk: 21.47 ± 1.71  Body msucle mass: TC group: baseline: 41.45 ± 3.44, 8wk: 42.11 ± 2.97; WBV: baseline: 42.33 ± 3.68, 8 wk: 42.76 ± 3.35, Con: baseline: 43.52 ± 3.76, 8wk: 43.04 ± 3.91 | No data | TC group: increased by 0.25 ± 0.28 m/s(37.8 ± 5.4%)  WBV group: 0.19 ± 0.23 m/s (30 ± 5.2%) |
| Bagheri et al. (2020)[38] | Iran | No data | BIA | No data | Vigorous | No data |
| Björkman et al. (2020)[39] | Finland | Intention to treat | Bioimpedance spectroscopy | No data | No data | No data |
| Chang et al. (2020)[40] | South Korea | Intention to treat | BIA | No data | Mild | Exercise: pre: 0.47 ± 0.15, post: 0.52 ± 0.15  Control: pre: 0.48 ± 0.14, post: 0.48 ± |
| Liao et al. (2020)[41] | Taiwan | Intention to treat | DXA | No data | Moderate- Vigorous | RET: T0: 0.75 ± 0.31, T1-T0: −0.17 ± 0.31, T2-T0 0.30 ± 0.28  Control: T0: 0.79 ± 0.37, T1-T0: −0.22 ± 0.35, T2-T0: 0.04 ± 0.27 |
| Oh et al. (2020)[42] | South Korea | No data | DXA | No data | No data | No data |
| Rooks et al. (2020)[43] | 13 countries | No data | DXA | Week 13:  Bimagrumab: baseline: 35.4 (8.89), change from baseline: 1.93 (1.74);  Placebo: 33.6 (6.89), change from baseline: 0.37 (1.24)  Week 25:  Bimagrumab: baseline: 35.4 (8.89), change from baseline: 2.02 (1.95);  Placebo: 33.6 (6.89), change from baseline: 0.08 (1.17) | No data | Bimagrumab: baseline:0.642 (0.1079); change from baseline: 0.14 [95% CI,0.09 to 0.18]  placebo: 0.656 (0.0836); change from baseline: 0.11 [95% CI, 0.05 to 0.16] |
| Espinoza et al. (2021)[44] | USA | No data | DXA | 1.0+-1.4 vs −0.2+-1.2 | No data | No data |
| Lee et al. (2021)[45] | Taiwan | Intention to treat | DXA | Total skeletal muscle mass  peRET: baseline: 33.46 ± 4.39, 3 months: 31.46 ± 8.79, 9 months: 33.14 ± 4.22  Control: baseline: 34.46 ± 3.97, 3 months: 34.04 ± 3.88, 9 months 33.84 ± 3.58  Appendicular lean muscle mass  peRET: baseline: 13.59 ± 1.90, 3 months: 13.58 ± 2.14, 9 months 13.64 ± 2.27  Control: baseline: 13.76 ± 2.05, 3 months: 13.56 ± 1.91, 9 months: 13.27 ± 2.05 | Moderate | peRET: baseline: 0.82 ± 0.21, 3 months: 0.71 ± 0.21, 9 months: 0.81± 0.20  Control: baseline: 0.90 ± 0.21, 3 months: 0.88 ± 0.19, 9 months: 0.87 ± 0.21 |
| Li et al. (2021)[46] | China | Both | BIA | No data | Vigorous | No data |
| Nasimi et al. (2021)[47] | Iran | No data | DXA | baseline: 17.8(14.9,19.38) vs 17.0(13.6,18.7)  change: 0.20(0.03,0.54) vs 0.03(0.38,0.53) | No data | 0.08(0.04,0.16) vs 0.00(0.03,0.06) |
| Osuka et al. (2021)[48] | Japan | Intention to treat | BIA | < Upper-extremity lean mass, kg >  Exercise + HMB: baseline: 2.79 ± 0.32, changes: − 0.02 (−0.06, 0.03)  Exercise + Placebo: baseline: 2.87 ± 0.29, changes: − 0.05 (−0.09, 0.00)  Education + HMB: baseline: 2.80 ± 0.34, changes: 0.02 (−0.03, 0.07)  Education + Placebo: baseline: 2.81 ± 0.37, changes: − 0.03 (−0.07, 0.01)  < Lower-extremity lean mass, kg >  Exercise + HMB: baseline: 9.2 ± 0.7, changes: 0.2 (0.1, 0.3)  Exercise + Placebo: baseline: 9.5 ± 1.1, changes: 0.2 (0.1, 0.3)  Education + HMB: baseline: 9.4 ± 1.1, changes: 0.2 (0.1, 0.3)  Education + Placebo: baseline: 9.4 ± 1.1, changes: 0.2 (0.1, 0.3)  < Appendicular lean mass, kg >  Exercise + HMB: baseline: 12.0 ± 0.9, changes: 0.1 (0.0, 0.3)  Exercise + Placebo: baseline:12.4 ± 1.3, changes: 0.2 (0.1, 0.3)  Education + HMB: baseline: 12.2 ± 1.4, changes: 0.2 (0.1, 0.4)  Education + Placebo: baseline: 12.3 ± 1.4, changes: 0.2 (0.1, 0.3)  < Fat-free mass, kg >  Exercise + HMB: baseline: 32.7 ± 1.9, changes: − 0.1 (−0.4, 0.3)  Exercise + Placebo: baseline: 33.2 ± 2.3, changes: 0.0 (−0.3, 0.2)  Education + HMB: baseline: 32.9 ± 2.6, changes: 0.3 (0.0, 0.6)  Education + Placebo: baseline: 33.0 ± 2.7, changes: 0.0 (−0.2, 0.3) | Moderate- Vigorous | < Usual gait speed, m/s >  Exercise + HMB: baseline: 1.29 ± 0.22, changes: 0.26 (0.20, 0.32)  Exercise + Placebo: baseline: 1.40 ± 0.24, changes: 0.15 (0.10, 0.20)  Education + HMB: baseline: 1.37 ± 0.27, changes: 0.05 (0.00, 0.11  Education + Placebo: baseline: 1.36 ± 0.16, changes: 0.04 (−0.01, 0.10)  < Maximal gait speed, m/s>  Exercise + HMB: baseline: 1.88 ± 0.33, changes: 0.26 (0.19, 0.33)  Exercise + Placebo: baseline: 2.01 ± 0.33, changes: 0.22 (0.15, 0.29)  Education + HMB: baseline: 1.99 ± 0.34, changes: 0.11 (0.04, 0.18)  Education + Placebo: baseline: 1.98 ± 0.25, changes: 0.07 (0.01, 0.13) |
| Seo et al. (2021)[49] | South Korea | Unclear | DXA and CT | < Fat free mass, kg >  RT: baseline: 31.6 ± 2.10, 16 wks: 31.9 ± 1.87  CON: baseline: 31.5 ± 2.58 , 16 wks: 31.5 ± 2.87  < Appendicular skeletal mass, kg >  RT: baseline: 12.3 ± 0.96, 16 wks: 12.4 ± 0.77  CON: baseline: 12.4 ± 0.95, 16 wks: 12.2 ± 1.10 | Moderate- Vigorous | RT: baseline: 0.96 ± 0.08, 16 wks: 1.14 ± 0.11  CON: baseline: 0.93 ± 0.09, 16 wks: 0.95 ± 0.09 |
| Lace study group et al.(2022)[50] | UK | Intention to treat | DXA | No data | No data | Perindopril: baseline 0.73±0.21, after 12 months: 0.84±0.25; no perinodopril: baseline 0.84±0.25, after 12 months: 1±1.11;  Leucine: baseline 0.74±0.23, after 12 months: 0.85±0.27; no leucine: baseline 0.75±0.24, after 12 months: 1±1.12 |

ASM = Appendicular skeletal muscle mass, ASMI = Appendicular muscle mass index, SMI = Total skeletal muscle mass index, SPPB = Short Physical Performance Battery, TUG = timed up and go test

†Median (25th interquartile range, 75th interquartile range)

(Continued)

| Study and Year | Fat mass (kg) | Body fat percentage (%) | Appendicular skeletal muscle mass index (SMMI, kg/m2) | Quality of life(QOL) | Handgrip strength (kg) |
| --- | --- | --- | --- | --- | --- |
| Alemán-Mateo et al. (2012)[1] | No Data | No Data | No Data | No Data | No Data |
| Bellomo et al.(2013)[2] | No Data | No Data | No Data | No Data | No Data |
| Papanicolaou et al. (2013)[3] | baseline: 16.59 (8.33) vs 18.56 (9.07)  change: 0.72 (0.55) vs 0.15 (0.69) | No Data | No Data | No Data | No Data |
| Liu et al. (2014)[4] | No Data | No Data | No Data | No Data | No Data |
| Bauer et al. (2015)[5] | No Data | No Data | No Data | No Data | Active: baseline: 20.9 (7.9), week 7 changes: 0.20 (3.2) , week 13 changes: 0.79 (3.6)  Control: baseline: 20.6 (7.5), week 7 changes: 0.34 (2.8), week 13 changes: 0.54 (3.2) |
| Zdzieblik et al. (2015)[6] | Fat mass(kg)  treatment group: baseline 28.1(SD7.09) after-intervention 22.7(SD7.08)  placebo group:  baseline 25.1(SD8.69) after-intervention 21.6(SD8.15) | Fat mass% treatment group:  Baseline:31.63 (SD4.58), after-intervention:  25.67(SD5.22); placebo group: baseline:29.5(SD5.53), after-intervention: 25.4(SD5.55) | No Data | No Data | No Data |
| Cramer et al. (2016)[7] | No Data | No Data | No Data | No Data | < SARCOPENIA >  Control: median baseline 23 (18,30), median improvement 0.67 (-0.67, 2.33) at 12 wks and 0.67 (-0.83, 3.00) at 24 wks  Experimental: median baseline 23 (18, 33), median improvement 0.67 (-0.67, 2.33) at 12 wks and 1.33 (0.17, 3.5) at 24 wks  < SEVERE SARCOPENIA >  Control: median baseline 16 (12, 19), median improvement 0.83 (-0.67, 2.00) at 12 wks and 1.33 (-0.33, 3.0) at 24 wks  Experimental: median baseline 17 (14, 20), median improvement 0.68 (-0.50, 2.17) at 12 wks and 0.67 (0.0, 2.8) at 24 wks |
| Kemmler et al. (2016)[8] | No Data | Baseline 37.3 (35.6 to 39.0) 37.5 (36.2 to 38.7) 36.4 (35.1 to 37.8)  Changes −.34 (−.78 to .10) −.52 (−.98 to −.06) −.28 (−.72 to .16) | Baseline 5.67 (5.50 to 5.83) 5.66 (5.48 to 5.83) 5.62 (5.47 to 5.78)  Changes .14 (.08 to .21) .11 (.04 to .19) −.07 (−.14 to −.00) | No Data | Baseline 18.8 (17.2 to 20.4) 20.9 (19.7 to 22.1) 19.4 (17.6 to 21.1)  Changes −.20 (−.95 to .55) −.04 (−.84 to .77) −1.17 (−1.94 to −.41) |
| Kim et al. (2016)[9] | No Data | E+N: baseline 38.0 ± 4.3, follow up 36.5 ± 4.7  E: baseline 36.9 ± 4.2, follow up 35.5 ± 4.5  N: baseline 37.8 ± 3.3, follow up 36.6 ± 3.5  HE: baseline 38.6 ± 5.2, follow up 37.4 ± 4.8 | No Data | No Data | E+N: baseline 19.2 ± 5.2, follow up 19.6 ± 5.2  E: baseline 20.5 ± 3.8, follow up 20.3 ± 3.8  N: baseline 20.4 ± 5.3, follow up 20.9 ± 5.1  HE: baseline 21.4 ± 4.5, follow up 21.1 ± 4.1 |
| Maltais et al. (2016)[10] | Nondairy: T1: 20.9 ± 7.1, T2: 20.8 ± 6.9  Dairy: T1: 19.5 ± 7.1, T2: 18.4 ± 7.8  Control: T1: 20.2 ± 7.8, T2: 19.3 ± 7.2 | No Data | Nondairy: T1: 9.34 ± 0.86, T2: 9.73 ± 0.90  Dairy: T1: 8.90 ± 0.94, T2: 9.17 ± 0.92  Control: T1: 8.94 ± 0.94, T2: 9.31 ± 1.13 | No Data | No Data |
| Maltais et al. (2016)[11] | No Data | No Data | Control: Baseline:8.9±0.9, post: 9.3 ± 1.1  EAA supp: Baseline:9.3±0.9, post:9.7 ± 0.9;  EAA milk: Baseline:8.9±0.9, post: 9.2 ± 0.9 | No Data | No Data |
| Maruya et al.(2016)[12] | No Data | Baseline: intervention group24.1 ± 6.3, control group 21.9 ± 6.0; post-intervention: intervention group 23.3 ± 7.1, control group 20.7 ± 6.5 | Baseline: intervention group 5.91 ± 0.66, control group 6.02 ± 0.57; post-intervention: intervention group 5.93 ± 0.65, control group 5.95 ± 0.75 | Baseline: intervention group 0.909 ± 0.128, control group 0.918 ± 0.114; post-intervention: intervention group 0.945 ± 0.100, control group 0.917 ± 0.117 | Baseline: intervention group 24.8 ± 7.4, control group 28.6 ± 6.5; post-intervention: intervention group 26.1 ± 8.0, control group 29.7 ± 7.8 |
| Rondanelli et al.(2016)[13] | Change from baseline: Dietary supplement group:-0.345 (0.205), Placebo group:-0.484(0.288) | No Data | Change from baseline: Dietary supplement group: 0.21 (0.071), Placebo group: -0.06(0.283) | SF-36 MCS score:  Change from baseline: Dietary supplement group: 4.5 (0.93), Placebo group: 2.48(1.16)  SF-36 PCS score:  Change from baseline: Dietary supplement group:1.32 (0.70), Placebo group: -0.77 (0.68) | Change from baseline: Dietary supplement group: 3.2 (0.50), Placebo group: -0.47(0.30) |
| Vasconcelos et al. (2016)[14] | No Data | No Data | No Data | EG: baseline: 81±21, week 10: 80±23  CG: baseline: 72±23, week 10: 78±22 | No Data |
| Chen et al. (2017)[15] | CON: baseline: 29.0 ± 6.2, 8 wks:29.8 ± 6.3 , 12 wks: 29.8 ± 5.9  RT: baseline: 27.9 ± 6.8, 8 wks: 26.9 ± 7.9, 12 wks: 27.3 ± 7.5  AT: baseline: 25.3 ± 5.6, 8 wks: 24.6 ± 5.6 , 12 wks:24.2 ± 5.8  CT: baseline: 25.7 ± 6.0 , 8 wks: 24.1 ± 5.6 , 12 wks: 24.6 ± 5.8 | CON: baseline: 39.8 ± 4.5, 8 wks: 40.5 ± 4.7, 12 wks: 41.0 ± 4.6  RT: baseline: 39.7 ± 5.6, 8 wks: 38.7 ± 6.4, 12 wks:38.8 ± 6.3  AT: baseline: 40.0 ± 4.4, 8 wks: 39.0 ± 4.6, 12 wks: 38.6 ± 4.9  CT: baseline: 39.7 ± 5.8, 8 wks: 37.4 ± 5.3, 12 wks: 38.3 ± 5.5 | No Data | No Data | CON: baseline: 22.2 ± 9.5, 8 wks: 19.7 ± 9.1, 12 wks: 18.6 ± 9.0  RT: baseline: 20.0 ± 7.0, 8 wks: 23.5 ± 7.3 , 12 wks: 22.1 ± 6.8  AT: baseline: 22.1 ± 7.6, 8 wks: 19.2 ± 6.7, 12 wks: 18.3 ± 7.3  CT: baseline: 26.4 ± 7.5, 8 wks: 24.3 ± 7.0, 12 wks: 23.1 ± 7.0 |
| Huang et al. (2017)[16] | ERT group: pre 25.52±5.01, post 24.94±5.24  Control group: pre 28.42±4.78, post 29.12±5.07 | ERT group: pre 41.21±3.98, post 40.34±3.74  Control group: pre 42.79±3.75, post 43.65±3.61 | SMI(%): total skeletal muscle mass/ total body mass  ERT group: pre 22.37±2.14, post 22.47±2.45  Control group: pre 22.03±2.25, post: 21.76±2.40 | No Data | No Data |
| Kemmler et al. (2017)[17] | No Data | WB-EMS:31.6 (30.5 to 32.9), WB-EMS+P:31.4 (30.4 to 32.4), CG:31.4 (0.34 to 0.94)  Changes: WB-EMS:−2.05 (−1.40 to −2.68), WB-EMS+P: −1.13 (−0.48 to −1.78), CG:0.30 (−0.24 to 0.12) | WB-EMS: 0.709 (0.695 to 0.734) WB-EMS:+P: 0.703 (0.681 to 0.723), CG: 0.710 (0.687 to 0.732)  Changes: WB-EMS:0.018 (0.011 to 0.026), WB-EMS+P:0.008 (0.001 to 0.015), CG:−0.008 (−0.001 to −0.016) | No Data | WB-EMS: 33.8 (31.0 to 36.6), WB-EMS+P:33.3 (31.2 to 35.4), CG:34.4 (31.1 to 36.6)  Changes: WB-EMS:1.90 (0.99 to 2.82), WB-EMS+P: 0.90 (−0.03 to 1.83), CG:−0.35 (−0.56 to 1.25) |
| Liao et al. (2017)[18] | EG: baseline: 26.39±5.34, 12 wks: 25.78±5.32  CG: baseline: 29.17±5.88, 12 wks: 29.60±6.01 | EG: baseline: 41.65±4.02, 12 wks: 40.89±3.77  CG: baseline: 43.40±5.23, 12 wks: 44.08±4.97 | No Data | No Data | EG: baseline: 12.99±3.03, 12 wks: 14.68±2.92  CG: baseline: 11.63±3.47, 12 wks: 11.01±3.75 |
| Park et al. (2017)[19] | No Data | Control: baseline: 40.4±3.6, 24 weeks: 40.8±4.2  Exercise: baseline: 41.0±3.6, 24 weeks: 39.0±3.9 | No Data | No Data | < Left handgrip, kg >  Control: baseline: 22.5±3.1, 24 weeks: 21.9±2.7  Exercise: baseline: 23.2±2.4, 24 weeks: 25.7±2.6  < Right handgrip, kg >  Control: baseline: 23.3±2.9, 24 weeks: 22.8±2.8  Exercise: baseline: 23.7±2.2, 24 weeks: 26.9±2.5 |
| Sammarco et al.(2017)[20] | No Data | No Data | No Data | No Data | Low-calorie diet (control): baseline 16.4 ± 3.89 , post intervention 16.4 ± 3.33 ; low-calorie high-protein diet: baseline 16.6 ± 3.74, post intervention 18.2 ± 3.56 |
| Wei et al.(2017)[21] | No Data | No Data | No Data | No Data | No Data |
| Wei et al.(2017)[22] | No Data | No Data | No Data | No Data | No Data |
| Chen et al. (2018)[23] | KT: baseline: 27.12 ± 7.28, week 8: 26.33 ± 8.05, week 12: 26.65 ± 7.78  CON: baseline: 28.41 ± 6.22, week 8: 28.83 ± 6.5, week 12: 28.74 ± 6.28 | No Data | KT: baseline: 5.57 ± 0.28, week 8: 5.66 ± 0.28 , week 12: 5.66 ± 0.25  CON: baseline: 5.45 ± 0.29, week 8: 5.39 ± 0.34, week 12: 5.39 ± 0.35 | No Data | < Left handgrip, kg >  KT: baseline: 16.75 ± 4.53, week 8: 21.04 ± 3.93, week 12: 19.72 ± 4.71  CON: baseline: 16.58 ± 4.2, week 8: 16.95 ± 3.72, week 12: 16.56 ± 3.84  < Right handgrip, kg >  KT: baseline: 17.24 ± 4.63, week 8: 21.95 ± 4.71, week 12: 20.95 ± 4.92  CON: baseline: 17.91 ± 4.91, week 8: 17.72 ± 4.83, week 12: 17.89 ± 4.84 |
| Kemmler et al. (2018)[24] | EMS&P: baseline: 59.8±13.6, changes: 0.19±2.65  CG: baseline: 67.8±22.6, changes: 5.28±6.92 | EMS&P: baseline: 34.4±4.6, changes: −1.58±1.60  CG: baseline: 34.6±3.7, changes: 0.25±1.87 | No Data | No Data | No Data |
| Liao et al. (2018)[25] | CG: T0: 13.97(1.87), T1: 13.78(1.65), T2: 13.56(1.56)  EG: T0: 14.21(2.03), T1: 14.39(2.11), T2: 14.26(2.20) | EG: T0: 41.65(4.02), T1: 40.89(3.77) , T2:42.02(3.86)  CG: T0: 43.40(5.23), T1: 44.08(4.97), T2:43.99(4.52) | EG: T0: 6.09(0.83), T1: ,6.37(0.76) T2: 6.23(0.92)  CG: T0: 6.06(0.68), T1:6.16(0.73) , T2: 6.10(0.61) | < 36-item Short Form Health Survey: physical function >  EG: T0: 91.11(13.38), T1: 94.14(11.27), T2: 96.26(17.32)  CG: T0: 76.52(17.57), T1: 74.20(19.21) , T2: 76.09(18.55)  < 36-item Short Form Health Survey: Physical component summary >  EG: T0: 83.27(13.65), T1: 86.38(10.62), T2: 88.93(10.14)  CG: T0: 68.87(17.12), T1: 65.39(17.55), T2: 65.91(18.38) | No Data |
| Piastra et al. (2018)[26] | No Data | No Data | RESISTANCE: baseline 6.48±2.75, f/u 7.36±2.31  POSTURAL: baseline 6.74±2.46, f/u 6.67±2.17 | No Data | RESISTANCE: baseline 17.84±4.91, f/u 19.86±5.22  POSTURAL: baseline 17.84±5.25, f/u17.55±4.85 kg) |
| Tsekoura et al. (2018)[27] | No Data | No Data | Group-based exercise: baseline: 5.7 ± 0.49, after 3-months intervention:5.94 ± 0.51, after 6 months follow-up:5.86 ± 0.46;  Home-based exercise: baseline: 5.64 ± 0.55, after 3-months intervention: 5.71 ± 0.54, after 6 months follow-up:5.69 ± 0.5;  Control: baseline: 5.58 ± 0.66, after 3-months intervention: 5.57 ± 0.65, after 6 months follow-up: 5.54 ± 0.65 | Group-based exercise: baseline: 57.08 ± 13.24, after 3-months intervention:64.37 ± 11.77, after 6 months follow-up:61.4 ± 15.73;  Home-based exercise: baseline: 56.51 ± 10.64, after 3-months intervention: 59.93 ± 11.28, after 6 months follow-up:58.5 ± 11.23;  Control: baseline: 53.21 ± 13.15, after 3-months intervention: 58.44 ± 12.74, after 6 months follow-up: 49.22 ± 10.49 | Group-based exercise: baseline: 17.22 ± 4.8, after 3-months intervention:20.58 ± 4.29, after 6 months follow-up:20.07 ± 4.35;  Home-based exercise: baseline: 19.2 ± 3.6, after 3-months intervention: 19.33 ± 4.51, after 6 months follow-up:18.68 ± 3.34;  Control: baseline: 17.43 ± 4.04, after 3-months intervention: 17.92 ± 4.14, after 6 months follow-up: 17.34 ± 3.77 |
| Von Berens et al. (2018)[28] | No Data | No Data | No Data | Physical component summary:  Intervention: baseline: 45.8 ± 7.6, after 6 months follow-up: 45.5 ± 8.1;  Con: baseline: 45.1 ± 8.1, after 6 months follow-up: 45.2 ± 9.1  Mental component summary:  Intervention: baseline: 50.4 ± 9.5, after 6 months follow-up: 53.1 ± 9.1;  Con: baseline: 53.1 ± 8.8, after 6 months follow-up: 56.1 ± 8.1 | No Data |
| Zhou et al. (2018)[29] | No Data | EA+EAA: baseline:33.17 ± 2.66, 4 weeks: 32.96 ± 2.88, 12 weeks: 27.3 0 ± 1.96, 20 weeks: 26.61 ± 1.92, 28 weeks: 25.91 ± 1.00;  EAA: baseline: 31.80 ± 2.83, 4 weeks: 31.64 ± 2.71, 12 weeks: 30.16 ± 2.84, 20 weeks: 30.16 ± 2.84, 28 weeks: 29.60 ± 2.75 | EA+EAA: baseline: 6.04 ± 0.50, 4 weeks: 6.07 ± 0.51, 12 weeks: 6.19 ± 0.52, 20 weeks: 6.94 ± 0.24, 28 weeks: 6.97 ± 0.24;  EAA: baseline: 5.94 ± 0.49, 4 weeks: 5.96 ± 0.48, 12 weeks: 5.96 ± 0.48, 20 weeks: 5.96 ± 0.45, 28 weeks: 6.41 ± 0.53 | No Data | No Data |
| Amasene et al. (2019)[30] | No Data | No Data | No Data | No Data | Protein: pre: 0.4 ± 0.09, post 0.4 ± 0.09  Placebo: pre: 0.3 ± 0.09, post: 0.3 ± 0.09 |
| Bo et al. (2019)[31] | No Data | No Data | No Data | No Data | No Data |
| Mafi et al. (2019)[32] | No Data | No Data | RT: baseline 9.65 ± 0.84, change after 8 wks 0.38 ± 0.27  RT+EP: baseline 9.31 ± 0.57, change after 8 wks 0.53 ± 0.20  EP: baseline 9.45 ± 0.74, change after 8 wks 0.34 ± 0.19  PL: baseline 9.33 ± 0.88, change after 8 wks -0.001 ± 0.005 | No Data | No Data |
| Nabuco et al. (2019)[33] | < Total fat mass, kg >  Whey: pre: 23.8 ± 5.4, post: 23.0 ± 5.3  Placebo: pre: 23.8 ± 5.9, post: 23.7 ± 6.4  < Trunk fat mass, kg >  Whey: pre: 13.6 ± 3.4, post: 13.0 ± 3.3  Placebo: pre: 13.9 ± 4.2, post: 13.8 ± 4.6 | Whey: pre: 40.9 ± 4.1, post: 39.7 ± 4.1  Placebo: pre: 39.6 ± 4.4, post: 39.5 ± 4.9 | No Data | No Data | No Data |
| Vikberg et al. (2019)[34] | No Data | No Data | CONTROL: baseline: 6.24±0.85, 10 weeks: 6.23±0.86  INTERVENTION: baseline: 6.17±0.87, 10 weeks: 6.40±0.89 | No Data | CONTROL: baseline: 30.0±11.1, 10 weeks: 30.5±10.6  INTERVENTION: baseline: 30.7±9.55, 10 weeks: 32.0±10.7 |
| Yamada et al.(2019)[35] | No Data | No Data | No Data | No Data | Ex+nutrition: change from baseline: 0.77±1.8, Ex: -0.05±2.27, Nutrition: -0.08±3.14, Control: -0.43 ±1.99 |
| Zhu et al. (2019a)[36] | No Data | No Data | Control: baseline: 5.23 ± 0.69, 12 wks: 5.21 ± 0.69, 24 wks: 5.35 ± 0.69  Exercise: baseline: 5.22 ± 0.68, 12 wks: 5.33 ± 0.71, 24 wks: 5.26 ± 0.65  Combined: baseline: 5.16 ± 0.59, 12 wks: 5.26 ± 0.67, 24 wks: 5.10 ± 0.44 | SF-12 (Physical)  Control: baseline 41.02 ± 11.94, 12 wks: 46.81 ± 6.82, 24 wks: 44.59 ± 8.13  Exercise: baseline 38.90 ± 9.90, 12 wks: 44.16 ± 9.14, 24 wks: 42.34 ± 8.85  Combined: baseline 41.20 ± 9.70, 12 wks: 48.97 ± 5.97, 24 wks 47.86 ± 6.42  SF-12 (Mental)  Control: baseline 49.14 ± 10.18, 12 wks: 53.30 ± 6.61, 24 wks: 55.37 ± 7.40  Exercise: baseline 49.25 ± 10.00, 12 wks: 54.81 ± 6.29, 24 wks: 55.13 ± 6.68  Combined: baseline 48.72 ± 10.62, 12 wks: 54.20 ± 6.92, 24 wks: 56.27 ± 6.71 | Control: baseline: 15.11 ± 5.63, 12 wks: 16.21 ± 5.01, 24 wks: 16.88 ± 4.77  Exercise: baseline: 15.77 ± 5.86, 12 wks: 19.37 ± 7.11, 24 wks: 19.64 ± 6.61  Combined: baseline: 13.81 ± 4.90, 12 wks: 18.17 ± 4.56, 24 wks: 15.50 ± 5.74 |
| Zhu et al. (2019b)[37] | No Data | No Data | No Data | No Data | TC group: baseline:14.87 ± 5.33, 8wk: 15.29 ± 5.38; WBV group: 13.41 ± 4.84, 8 wks: 15.09 ± 4.44; Con: baseline:15.50 ± 3.97, 8wks:15.16 ± 4.52 |
| Bagheri et al. (2020)[38] | No Data | No Data | No Data | No Data | E+R= 6.9 kg (d= -3.20)  R+E= 2.3 kg (d= -0.98) |
| Björkman et al. (2020)[39] | No Data | No Data | CRi-SMI (cm2/omega):  Baseline:  Con: 1.29 ±0.34; Isocaloric: 1.27 ±0.35; Protein: 1.28±0.34;  12 month (change from baseline):  Con: -0.01 (-0.05, 0.04), Isocaloric:-0.05 (-0.09, -0.01), and Protein: -0.05 (-0.10, -0.01) | No Data | Baseline: Con: 19.4 ±5.9; Isocaloric: 20.5 ±6.7; Protein: 18.5±6.5;  12 month (change from baseline):  Con: -1.9 (-2.7, 1.1), Isocaloric:-1.7 (-2.5, -0.99), and Protein: -1.9 (2.6, -1.1) |
| Chang et al. (2020)[40] | No Data | No Data | Exercise: pre: 5.29 ± 0.38, post: 5.31 ± 0.36  Control: pre: 5.30 ± 0.31, post: 5.27 ± 0.31 | No Data | Exercise: pre: 12.3 ± 4.9, post: 12.9 ± 4.6  Control: pre: 13.3 ± 3.8, post: 13.3 ± 3.7 |
| Liao et al. (2020)[41] | No Data | No Data | RET: T0: 6.22 ± 1.10, T2-T0: 0.17 ± 0.32  Control: 5.95 ± 0.99, T2-T0: −0.07 ± 0.34 | No Data | No Data |
| Oh et al. (2020)[42] | No Data | No Data | No Data | < Euro Quality of Life Questionnaire Five-Dimensional Classification (EQ-5D) >  Exp: baseline 0.18 ± 0.26, 3 wks 0.67 ± 0.09, 3 mo 0.76 ± 0.08, 6 mo 0.77 ± 0.83  Control: baseline 0.15 ± 0.22, 3 wks 0.38 ± 0.22, 3 mo 0.54 ± 0.24, 6 mo 0.57 ± 0.20 | < Right hand >  Exp: baseline 14.21 ± 3.77, 3 wks 16.15±4.39, 3 mo 17.81 ± 5.16, 6 mo 17.31 ± 5.68  Control: baseline 13.26 ± 3.11, 3 wks 14.79 ± 3.01, 3 mo 15.07 ± 2.82, 6 mo 15.08 ± 3.12  < Left hand >  Exp: baseline 13.58 ± 3.59, 3 wks 15.50 ± 4.35, 3 mo 17.07 ± 5.44, 6 mo 17.00 ± 5.18  Control: baseline 12.47 ± 3.23, 3 wks 13.94 ± 3.31, 3 mo 15.28 ± 3.23, 6 mo 15.13 ± 3.51 |
| Rooks et al. (2020)[43] | Week 13:  Bimagrumab:baseline: 22.7 (8.74), change from baseline: -1.29 (1.8);  Placebo: 21.2 (7.5), change from baseline: 0.1 (1.3)  Week 25:  Bimagrumab:baseline: 22.7 (8.74), change from baseline: -3.24 (2.5);  Placebo: 21.2 (7.5), change from baseline: 0.6 (1.6) | No Data | Bimagrumab:baseline: 5.7 (0.82), change from baseline: 7% (95% CI, 6% to 8%);  Placebo: 5.5 (0.75), change from baseline: 1% (95% CI, 0% to 2%) | No Data | No Data |
| Espinoza et al. (2021)[44] | −0.2+-0.99 vs −0.1+-0.92 | −1.764+-2.9262 vs −1.150+-4.7994 | No Data | No Data | No Data |
| Lee et al. (2021)[45] | No Data | peRET: baseline: 41.27 ± 6.07, 3 months: 38.58 ± 8.34, 9 months: 39.91 ± 5.06  Control: baseline 44.20 ± 7.71, 3 months: 43.24 ± 7.43, 9 months: 44.03 ± 6.80 | peRET: baseline: 5.01 ± 0.83, 3 months: 5.01 ± 0.94, 9 months: 5.03 ± 0.98  Control: baseline: 5.12 ± 1.00, 3 months: 5.01 ± 0.82, 9 months: 5.13 ± 0.90 | No Data | peRET: baseline 20.40 ± 4.00, 3 months: 20.35 ± 3.53, 9 months: 19.40 ± 3.85  Control: baseline 19.34 ± 6.36, 3 months: 18.11 ± 5.91, 9 months: 17.19 ± 5.42 |
| Li et al. (2021)[46] | Baseline 19.50 ± 6.39 vs 18.80 ± 5.71 vs 18.25 ± 4.70 vs 17.60 ± 5.58  Week 12: 16.87 ± 6.12 vs 18.37 ± 6.07 vs 15.39±4.91 vs 18.13 ± 5.43 | Baseline 33.14 ± 8.88 vs 32.92 ± 7.15 vs 31.49 ± 6.88 vs 31.25 ± 8.40  Week 12: 29.35 ± 9.04 vs 32.35 ± 8.10 vs 27.53 ± 7.62 vs 32.36 ± 7.84 | No Data | No Data | baseline: 19.84+-7.9 vs 20.66+-6.8 vs 21.59+-6.67 vs 23.71+-6.98  week12: 23.76+-7.27 vs 23.62+-5.83 vs 24.83+- 6.26 vs 20.16+-8.57 |
| Nasimi et al. (2021)[47] | baseline:16.62+-5.09 vs 18.3+-04.83  change: 0.37(0.76,0.00) vs 0.41(0.81,0.00) | No Data | No Data | No Data | baseline: 13.6(11.1,14.7) vs 13.6(11.3,15.8)  change: 4.15(2.64,5.93) vs 0.37(0.75,2.26) |
| Osuka et al. (2021)[48] | Exercise + HMB: baseline: 15.5 ± 4.4, changes: − 0.3 (−0.6, 0.1)  Exercise + Placebo: baseline: 14.4 ± 4.0, changes: − 0.2 (−0.6, 0.2)  Education + HMB: baseline: 12.9 ± 4.4, changes: − 0.6 (−0.9, −0.2)  Education + Placebo: baseline: 14.8 ± 4.7, changes: − 0.1 (−0.4, 0.2) | Exercise + HMB: baseline: 31.7 ± 6.3, changes: − 0.4 (−1.0, 0.2)  Exercise + Placebo: baseline: 29.8 ± 6.4, changes: − 0.3 (−0.9, 0.4)  Education + HMB: baseline: 27.6 ± 7.4, changes: − 1.1 (−1.8, −0.5)  Education + Placebo: baseline: 30.5 ± 7.1, changes: − 0.2 (−0.8, 0.4) | Exercise + HMB: baseline: 5.31 ± 0.32, changes: 0.06 (0.01, 0.11)  Exercise + Placebo: baseline: 5.40 ± 0.31, changes: 0.08 (0.03, 0.13)  Education + HMB: baseline: 5.32 ± 0.35, changes:0.09 (0.04, 0.14)  Education + Placebo: baseline: 5.29 ± 0.38, changes: 0.08 (0.03, 0.13) | No Data | Exercise + HMB: baseline: 19.3 ± 3.8, changes: 0.8 (−0.4, 2.0)  Exercise + Placebo: baseline: 21.5 ± 3.5, changes: − 0.0 (−0.9, 0.8)  Education + HMB: baseline: 20.6 ± 4.3, changes: 0.1 (−0.6, 0.8)  Education + Placebo: baseline: 20.4 ± 4.1, changes: − 0.3 (−1.1, 0.6) |
| Seo et al. (2021)[49] | RT: baseline: 19.1 ± 3.11, 16 wks: 19.2 ± 3.21  CON: baseline: 17.5 ± 3.58, 16 wks: 17.4 ± 3.77 | RT: baseline: 36.3 ± 3.74, 16 wks: 36.3 ± 3.82  CON: baseline: 34.4 ± 3.85, 16 wks: 34.3 ± 4.14 | No Data | No Data | RT: baseline: 18.6 ± 3.07, 16 wks: 17.3 ± 3.61  CON: baseline: 0.96 ± 0.08, 16 wks: 1.14 ± 0.11 |
| Lace study group et al.(2022)[50] | No Data | No Data | Perindopril: baseline 6.47±1.03, after 12 months: 6.09±2.18; no perinodopril: baseline 6.38±1.41, after 12 months: 6.22±1.89  Leucine:baseline 6.44±1.34, after 12 months: 6.03±2.23; no leucine: baseline 6.42±1.12, after 12 months: 6.28±1.83 | EQ5D main score: Perindopril: baseline 0.77±0.11, after 12 months: 0.77±0.10; no perinodopril: baseline 0.77±0.10, after 12 months: 0.81±0.13;  Leucine:baseline 0.77±0.11, after 12 months: 0.81±0.13; no leucine: baseline 0.78±0.10, after 12 months: 0.77±0.10  EQ5D thermometer: Perindopril: baseline 69±17, after 12 months: 69±18; no perinodopril: baseline 74±13, after 12 months: 75±14;  Leucine:baseline 73±17, after 12 months: 72±20; no leucine: baseline 70±13, after 12 months: 72±13 | Perindopril: baseline 18.3±6.7, after 12 months: 20.4±6.7; no perinodopril: baseline 17.8±6.9, after 12 months: 19±6.9  Leucine:baseline 17.8±6.8, after 12 months: 19.4±6.5; no leucine: baseline 18.3±6.8, after 12 months: 19.9±7.1 |

ASM = Appendicular skeletal muscle mass, ASMI = Appendicular muscle mass index, SMI = Total skeletal muscle mass index, SPPB = Short Physical Performance Battery, TUG = timed up and go test

†Median (25th interquartile range, 75th interquartile range)

Table S3a. Treatment comparisons using node-splitting model on 5TSTS

|  | Comparison | P value | MD(CrI) |
| --- | --- | --- | --- |
| 1 | Nutrition vs. RT | 0.7413 |  |
|  | -> direct |  | -1.3 (-2.6, 0.53) |
|  | -> indirect |  | -0.83 (-3.2, 1.5) |
|  | -> network |  | -1.0 (-2.3, 0.36) |
| 2 | Nutrition vs. RT+nutrition | 0.041575 |  |
|  | -> direct |  | -1.9 (-2.8, -0.53) |
|  | -> indirect |  | 0.55 (-1.1, 2.1) |
|  | -> network |  | -1.0 (-2.2, 0.41) |
| 3 | Placebo vs. RT | 0.89035 |  |
|  | -> direct |  | -1.6 (-3.3, -0.020) |
|  | -> indirect |  | -1.8 (-4.6, 1.1) |
|  | -> network |  | -1.6 (-2.8, -0.45) |
| 4 | Placebo vs. RT+nutrition | 0.575075 |  |
|  | -> direct |  | -1.5 (-2.4, -0.35) |
|  | -> indirect |  | -2.2 (-4.7, 0.57) |
|  | -> network |  | -1.6 (-2.5, -0.48) |
| 5 | RT vs. RT+nutrition | 0.23645 |  |
|  | -> direct |  | -0.30 (-1.5, 1.1) |
|  | -> indirect |  | 2.0 (-1.7, 5.6) |
|  | -> network |  | 0.032 (-1.1, 1.3) |

Table S3b. Treatment comparisons using node-splitting model on TUG

|  | Comparison | P value | MD(CrI) |
| --- | --- | --- | --- |
| 1 | Nutrition vs. RT | 0.8244 |  |
|  | -> direct |  | -0.18 (-1.7, 1.4) |
|  | -> indirect |  | -0.50 (-3.3, 2.3) |
|  | -> network |  | -0.45 (-1.6, 0.66) |
| 2 | Nutrition vs. RT+nutrition | 0.764375 |  |
|  | -> direct |  | -0.20 (-1.8, 1.4) |
|  | -> indirect |  | -0.70 (-4.0, 2.5) |
|  | -> network |  | -0.36 (-1.6, 0.80) |
| 3 | Placebo vs. RT | 0.253375 |  |
|  | -> direct |  | -0.96 (-2.0, -0.097) |
|  | -> indirect |  | 0.71 (-2.4, 3.7) |
|  | -> network |  | -0.85 (-1.7, -0.11) |
| 4 | Placebo vs. RT+nutrition | 0.015075 |  |
|  | -> direct |  | -0.14 (-0.97, 0.65) |
|  | -> indirect |  | -2.9 (-4.8, -0.98) |
|  | -> network |  | -0.76 (-1.8, 0.17) |
| 5 | RT vs. RT+nutrition | 0.1448 |  |
|  | -> direct |  | -0.12 (-0.92, 0.33) |
|  | -> indirect |  | 0.97 (-0.55, 2.5) |
|  | -> network |  | 0.087 (-0.92, 1.1) |

Table S3c. Treatment comparisons using node-splitting model on SPPB

|  | Comparison | P value | MD(CrI) |
| --- | --- | --- | --- |
| 1 | Placebo vs. RT | 0.3832 |  |
|  | -> direct |  | -0.18 (-1.2, 0.85) |
|  | -> indirect |  | 0.59 (-0.93, 2.1) |
|  | -> network |  | 0.071 (-0.78, 0.90) |
| 2 | Placebo vs. RT+nutrition | 0.3715 |  |
|  | -> direct |  | 0.20 (-0.78, 1.1) |
|  | -> indirect |  | -0.60 (-2.1, 0.96) |
|  | -> network |  | -0.00034 (-0.83, 0.78) |
| 3 | RT vs. RT+nutrition | 0.3892 |  |
|  | -> direct |  | -0.42 (-1.6, 0.73) |
|  | -> indirect |  | 0.36 (-1.0, 1.8) |
|  | -> network |  | -0.068 (-0.97, 0.80) |

Table S3d. Treatment comparisons using node-splitting model on gait speed

|  | Comparison | P value | MD(CrI) |
| --- | --- | --- | --- |
| 1 | Nutrition vs. RT | 0.489175 |  |
|  | -> direct |  | 0.10 (-0.17, 0.37) |
|  | -> indirect |  | -0.0027 (-0.16, 0.16) |
|  | -> network |  | 0.024 (-0.11, 0.15) |
| 2 | Nutrition vs. RT+nutrition | 0.570925 |  |
|  | -> direct |  | 0.21 (-0.061, 0.48) |
|  | -> indirect |  | 0.10 (-0.17, 0.40) |
|  | -> network |  | 0.15 (-0.019, 0.34) |
| 3 | Placebo vs. RT+nutrition | 0.6237 |  |
|  | -> direct |  | 0.22 (-0.049, 0.49) |
|  | -> indirect |  | 0.13 (-0.10, 0.40) |
|  | -> network |  | 0.17 (0.014, 0.34) |

Table S3e. Treatment comparisons using node-splitting model on ASMI

|  | Comparison | P value | MD(CrI) |
| --- | --- | --- | --- |
| 1 | AT+RT vs. Nutrition | 0.550825 |  |
|  | -> direct |  | 0.15 (-0.22, 0.53) |
|  | -> indirect |  | 0.012 (-0.29, 0.30) |
|  | -> network |  | 0.048 (-0.18, 0.27) |
| 2 | AT+RT vs. RT | 0.61005 |  |
|  | -> direct |  | -0.50 (-2.9, 1.7) |
|  | -> indirect |  | 0.097 (-0.13, 0.33) |
|  | -> network |  | 0.084 (-0.15, 0.32) |
| 3 | AT+RT+nutrition vs. Nutrition | 0.59785 |  |
|  | -> direct |  | 0.062 (-0.33, 0.46) |
|  | -> indirect |  | -0.067 (-0.38, 0.23) |
|  | -> network |  | -0.035 (-0.27, 0.20) |
| 4 | Nutrition vs. RT | 0.7025 |  |
|  | -> direct |  | 0.012 (-0.22, 0.24) |
|  | -> indirect |  | 0.068 (-0.14, 0.28) |
|  | -> network |  | 0.037 (-0.11, 0.18) |
| 5 | Nutrition vs. RT+nutrition | 0.6997 |  |
|  | -> direct |  | 0.062 (-0.13, 0.26) |
|  | -> indirect |  | 0.12 (-0.14, 0.38) |
|  | -> network |  | 0.086 (-0.076, 0.25) |
| 6 | Nutrition vs. Whole body EMS+nutrition | 0.995175 |  |
|  | -> direct |  | 0.010 (-0.28, 0.30) |
|  | -> indirect |  | 0.012 (-0.31, 0.32) |
|  | -> network |  | -0.019 (-0.22, 0.18) |
| 7 | Placebo vs. RT | 0.7508 |  |
|  | -> direct |  | 0.18 (0.079, 0.29) |
|  | -> indirect |  | 0.29 (-0.39, 0.97) |
|  | -> network |  | 0.19 (0.080, 0.30) |
| 8 | Placebo vs. RT+nutrition | 0.6859 |  |
|  | -> direct |  | 0.25 (0.085, 0.42) |
|  | -> indirect |  | 0.10 (-0.57, 0.79) |
|  | -> network |  | 0.24 (0.096, 0.38) |
| 9 | RT vs. RT+nutrition | 0.781675 |  |
|  | -> direct |  | 0.034 (-0.14, 0.22) |
|  | -> indirect |  | 0.075 (-0.18, 0.32) |
|  | -> network |  | 0.049 (-0.11, 0.21) |

Table S3f. Treatment comparisons using node-splitting model on leg muscle mass

|  | Comparison | P value | MD(CrI) |
| --- | --- | --- | --- |
| 1 | Nutrition vs. RT | 0.32805 |  |
|  | -> direct |  | -0.0042 (-0.91, 0.92) |
|  | -> indirect |  | 0.55 (-0.43, 1.6) |
|  | -> network |  | 0.27 (-0.35, 0.94) |
| 2 | Nutrition vs. RT+nutrition | 0.62235 |  |
|  | -> direct |  | 0.0040 (-1.1, 1.1) |
|  | -> indirect |  | 0.37 (-0.96, 1.7) |
|  | -> network |  | 0.11 (-0.57, 0.85) |
| 3 | Placebo vs. RT | 0.672675 |  |
|  | -> direct |  | 0.51 (-0.10, 1.2) |
|  | -> indirect |  | 0.20 (-1.3, 1.8) |
|  | -> network |  | 0.46 (-0.012, 0.98) |
| 4 | Placebo vs. RT+nutrition | 0.34815 |  |
|  | -> direct |  | 0.066 (-0.68, 0.89) |
|  | -> indirect |  | 0.65 (-0.51, 1.8) |
|  | -> network |  | 0.31 (-0.29, 0.94) |
| 5 | RT vs. RT+nutrition | 0.63275 |  |
|  | -> direct |  | -0.00076 (-0.31, 0.31) |
|  | -> indirect |  | -0.38 (-1.8, 1.1) |
|  | -> network |  | -0.16 (-0.75, 0.44) |

Table S3g. Treatment comparisons using node-splitting model on skeletal muscle mass

|  | Comparison | P value | MD(CrI) |
| --- | --- | --- | --- |
| 1 | AT+RT vs. Nutrition | 0.3977 |  |
|  | -> direct |  | 0.56 (-0.94, 2.1) |
|  | -> indirect |  | -0.22 (-1.3, 0.86) |
|  | -> network |  | 0.026 (-0.83, 0.88) |
| 2 | AT+RT vs. RT | 0.5486 |  |
|  | -> direct |  | -0.61 (-3.1, 1.9) |
|  | -> indirect |  | 0.21 (-0.75, 1.2) |
|  | -> network |  | 0.092 (-0.79, 0.96) |
| 3 | Nutrition vs. RT | 0.95025 |  |
|  | -> direct |  | 0.080 (-0.77, 0.95) |
|  | -> indirect |  | 0.045 (-0.92, 0.94) |
|  | -> network |  | 0.069 (-0.52, 0.64) |
| 4 | Nutrition vs. RT+nutrition | 0.1331 |  |
|  | -> direct |  | -0.022 (-0.71, 0.69) |
|  | -> indirect |  | 0.86 (-0.067, 1.7) |
|  | -> network |  | 0.30 (-0.32, 0.90) |
| 5 | Placebo vs. RT | 0.80655 |  |
|  | -> direct |  | 0.52 (0.013, 1.0) |
|  | -> indirect |  | 0.31 (-1.3, 2.0) |
|  | -> network |  | 0.58 (0.11, 1.0) |
| 6 | Placebo vs. RT+nutrition | 0.560825 |  |
|  | -> direct |  | 0.78 (0.17, 1.4) |
|  | -> indirect |  | 1.3 (-0.39, 3.0) |
|  | -> network |  | 0.81 (0.30, 1.3) |
| 7 | RT vs. RT+nutrition | 0.1777 |  |
|  | -> direct |  | 0.0039 (-0.53, 0.62) |
|  | -> indirect |  | 0.71 (-0.15, 1.5) |
|  | -> network |  | 0.23 (-0.34, 0.81) |

Table S3h. Treatment comparisons using node-splitting model on handgrip strength

|  | Comparison | P value | MD(CrI) |
| --- | --- | --- | --- |
| 1 | AT vs. AT+RT | 0.920675 |  |
|  | -> direct |  | 0.86 (-4.1, 5.8) |
|  | -> indirect |  | 1.1 (-2.2, 4.6) |
|  | -> network |  | 1.5 (-1.3, 4.4) |
| 2 | AT vs. RT | 0.034925 |  |
|  | -> direct |  | 6.4 (1.4, 11) |
|  | -> indirect |  | 0.073 (-2.9, 3.1) |
|  | -> network |  | 1.3 (-1.2, 4.1) |
| 3 | AT+RT vs. Nutrition | 0.3837 |  |
|  | -> direct |  | 0.96 (-2.8, 4.7) |
|  | -> indirect |  | -0.92 (-3.0, 1.2) |
|  | -> network |  | -0.68 (-2.5, 1.2) |
| 4 | AT+RT vs. RT | 0.01575 |  |
|  | -> direct |  | 5.6 (0.66, 11) |
|  | -> indirect |  | -1.1 (-3.1, 0.93) |
|  | -> network |  | -0.22 (-2.1, 1.8) |
| 5 | AT+RT+nutrition vs. Nutrition | 0.263675 |  |
|  | -> direct |  | 0.69 (-3.1, 4.4) |
|  | -> indirect |  | -1.9 (-4.8, 0.89) |
|  | -> network |  | -1.5 (-3.7, 0.79) |
| 6 | Nutrition vs. RT | 0.504925 |  |
|  | -> direct |  | -0.038 (-2.3, 2.3) |
|  | -> indirect |  | 0.90 (-0.83, 2.7) |
|  | -> network |  | 0.47 (-0.85, 1.9) |
| 7 | Nutrition vs. RT+nutrition | 0.8125 |  |
|  | -> direct |  | 0.76 (-1.6, 3.1) |
|  | -> indirect |  | 1.2 (-2.0, 4.2) |
|  | -> network |  | 0.75 (-0.95, 2.4) |
| 8 | Nutrition vs. whole body EMS+nutrition | 0.478225 |  |
|  | -> direct |  | 1.0 (-2.2, 4.2) |
|  | -> indirect |  | -0.53 (-3.8, 2.7) |
|  | -> network |  | 0.23 (-1.8, 2.3) |
| 9 | RT vs. RT+nutrition | 0.80985 |  |
|  | -> direct |  | 0.81 (-1.4, 3.0) |
|  | -> indirect |  | 0.36 (-2.8, 3.2) |
|  | -> network |  | 0.28 (-1.5, 1.9) |
| 10 | Whole body EMS vs.  whole body EMS+nutrition | 0.9087 |  |
|  | -> direct |  | 0.17 (-3.0, 3.3) |
|  | -> indirect |  | 0.49 (-4.3, 5.3) |
|  | -> network |  | 0.38 (-2.1, 2.9) |

Table S3i. Treatment comparisons using node-splitting model on chest press

|  | Comparison | P value | MD(CrI) |
| --- | --- | --- | --- |
| 1 | Placebo vs. RT | 0.7368 |  |
|  | -> direct |  | 3.6 (-5.6, 13) |
|  | -> indirect |  | 7.3 (-14., 28) |
|  | -> network |  | 3.5 (-4.1, 11) |

Table S3j. Treatment comparisons using node-splitting model on leg press

|  | Comparison | P value | MD(CrI) |
| --- | --- | --- | --- |
| 1 | AT+RT vs. RT | 0.8736 |  |
|  | -> direct |  | 2.5 (-13., 18) |
|  | -> indirect |  | 4.1 (-7.9, 16) |
|  | -> network |  | 3.7 (-4.9, 12) |
| 2 | Nutrition vs. RT | 0.445125 |  |
|  | -> direct |  | 11. (1.2, 20) |
|  | -> indirect |  | 5.4 (-6.7, 18) |
|  | -> network |  | 8.2 (1.3, 15) |
| 3 | Nutrition vs. RT+nutrition | 0.43045 |  |
|  | -> direct |  | 14. (4.3, 22) |
|  | -> indirect |  | 6.1 (-10., 24) |
|  | -> network |  | 12. (4.4, 20) |
| 4 | Placebo vs. RT | 0.4225 |  |
|  | -> direct |  | 8.8 (3.6, 14) |
|  | -> indirect |  | -1.1 (-26., 23) |
|  | -> network |  | 8.3 (3.8, 13) |
| 5 | Placebo vs. RT+nutrition | 0.709425 |  |
|  | -> direct |  | 13. (4.6, 21) |
|  | -> indirect |  | 18. (-4.6, 40) |
|  | -> network |  | 12. (5.6, 19) |
| 6 | RT vs. RT+nutrition | 0.241925 |  |
|  | -> direct |  | 2.5 (-3.0, 8.7) |
|  | -> indirect |  | -5.4 (-18., 7.0) |
|  | -> network |  | 4.0 (-3.0, 11) |

Table S4. Egger's test for publication bias

| Egger's test | Intercept | 95% CI | Significance level |
| --- | --- | --- | --- |
| 5 times sit to stand (sec) | -0.92 | -0.80 to 6.25 | P=0.78 |
| 30-second chair stand test | 13.55 | -6.93 to 34.02 | P=0.14 |
| Timed up and go test | -7.84 | -18.86 to 3.17 | P=0.14 |
| Short physical performance battery | 0.38 | -7.81 to 8.57 | P=0.91 |
| Gait speed | 4.70 | -0.33 to 9.73 | P=0.07 |
| Six min walk test | -1.00 | -17.88 to 15.88 | P=0.86 |
| Appendicular skeletal muscle index | 3.41 | -0.92 to 7.73 | P=0.12 |
| Leg muscle mass | 6.64 | 2.17 to 11.11 | P=0.01 |
| Skeletal muscle mass | 2.72 | -0.80 to 6.24 | P=0.12 |
| Handgrip strength | 5.31 | 1.72 to 8.90 | P=0.01 |
| Chest press | 2.28 | -5.03 to 9.59 | P=0.49 |
| Leg press | 5.51 | 2.48 to 8.54 | P=0.01 |
| Quality of life | 10.03 | 4.03 to 16.02 | P=0.04 |

S1. Search terms and search string

**Search terms:**

**sarcopenia:** "sarcopenia"[MeSH Terms] OR "sarcopenia"[All Fields] OR "sarcopenia's"[All Fields]

**sarcopenia:** "sarcopenia"[MeSH Terms] OR "sarcopenia"[All Fields] OR "sarcopenia's"[All Fields]

**exercise:** "exercise"[MeSH Terms] OR "exercise"[All Fields] OR "exercises"[All Fields] OR "exercise therapy"[MeSH Terms] OR ("exercise"[All Fields] AND "therapy"[All Fields]) OR "exercise therapy"[All Fields] OR "exercising"[All Fields] OR "exercise's"[All Fields] OR "exercised"[All Fields] OR "exerciser"[All Fields] OR "exercisers"[All Fields]

**diet:** "diet"[MeSH Terms] OR "diet"[All Fields]

**food:** "food"[MeSH Terms] OR "food"[All Fields]

**nutrition:** "nutrition's"[All Fields] OR "nutritional status"[MeSH Terms] OR ("nutritional"[All Fields] AND "status"[All Fields]) OR "nutritional status"[All Fields] OR "nutrition"[All Fields] OR "nutritional sciences"[MeSH Terms] OR ("nutritional"[All Fields] AND "sciences"[All Fields]) OR "nutritional sciences"[All Fields] OR "nutritional"[All Fields] OR "nutritionals"[All Fields] OR "nutritions"[All Fields] OR "nutritive"[All Fields]

**nutrition therapy:** "nutritional support"[MeSH Terms] OR ("nutritional"[All Fields] AND "support"[All Fields]) OR "nutritional support"[All Fields] OR ("nutrition"[All Fields] AND "therapy"[All Fields]) OR "nutrition therapy"[All Fields] OR "nutrition therapy"[MeSH Terms]

**drug therapy[MeSH Terms]:** "drug therapy"[MeSH Terms]

**Search string:**

(("sarcopenia"[MeSH Terms] OR "sarcopenia"[All Fields] OR "sarcopenia s"[All Fields] OR "sarcopeni*"[All Fields]) AND ("train*"[All Fields] OR "physical activity"[All Fields] OR "physical activity"[All Fields] OR "exercise"[All Fields] OR "diet"[All Fields] OR "nutr*"[All Fields] OR "drug therapy"[All Fields]) AND ("sarcopenia"[MeSH Terms] OR "sarcopenia"[All Fields] OR "sarcopenia s"[All Fields] OR ("exercise"[MeSH Terms] OR "exercise"[All Fields] OR "exercises"[All Fields] OR "exercise therapy"[MeSH Terms] OR ("exercise"[All Fields] AND "therapy"[All Fields]) OR "exercise therapy"[All Fields] OR "exercising"[All Fields] OR "exercise s"[All Fields] OR "exercised"[All Fields] OR "exerciser"[All Fields] OR "exercisers"[All Fields]) OR ("diet"[MeSH Terms] OR "diet"[All Fields]) OR ("food"[MeSH Terms] OR "food"[All Fields]) OR ("nutrition s"[All Fields] OR "nutritional status"[MeSH Terms] OR ("nutritional"[All Fields] AND "status"[All Fields]) OR "nutritional status"[All Fields] OR "nutrition"[All Fields] OR "nutritional sciences"[MeSH Terms] OR ("nutritional"[All Fields] AND "sciences"[All Fields]) OR "nutritional sciences"[All Fields] OR "nutritional"[All Fields] OR "nutritionals"[All Fields] OR "nutritions"[All Fields] OR "nutritive"[All Fields]) OR ("nutritional support"[MeSH Terms] OR ("nutritional"[All Fields] AND "support"[All Fields]) OR "nutritional support"[All Fields] OR ("nutrition"[All Fields] AND "therapy"[All Fields]) OR "nutrition therapy"[All Fields] OR "nutrition therapy"[MeSH Terms]) OR "drug therapy"[MeSH Terms])) AND (randomizedcontrolledtrial[Filter])
